# The NAT10/c-Myc positive feedback loop orchestrates tRNA ac^4^C modification and OTUB1-mediated protein stabilization to drive anaplastic thyroid carcinoma progression

**DOI:** 10.1186/s11658-026-00977-x

**Published:** 2026-06-12

**Authors:** Bo Wei, Haixi Zhao, Shi Chang, Wenlong Wang

**Affiliations:** 1 Division of Thyroid Surgery, Department of General Surgery, Xiangya Hospital, Central South University, No.87 Xiangya Road, Changsha, 410008 Hunan China; 2 College of Life Science and Technology, Huazhong University of Science and Technology, Wuhan, 430074 China; 3 Hunan Provincial Clinical Medical Research Centre for Thyroid Diseases, No.87 Xiangya Road, Changsha, 410008 Hunan China; 4 Hunan Engineering Research Center for Thyroid and Related Diseases Diagnosis and Treatment Technology, No.87 Xiangya Road, Changsha, 410008 Hunan China; 5 National Clinical Research Center for Geriatric Disorders, Xiangya Hospital, Central South University, No.87 Xiangya Road, Changsha, 410008 Hunan China; 6 Furong Laboratory, Changsha, 410008 Hunan China; 7 Department of Breast Surgery, Xiangya Hospital, Central South University, Changsha, 410008 Hunan China; 8 Clinical Research Center for Breast Cancer in Hunan Province, Changsha, Hunan China

**Keywords:** Anaplastic thyroid carcinoma, NAT10, c-Myc, Ac^4^C modification, Doxorubicin

## Abstract

**Background:**

Epitranscriptomic regulation of tRNA modifications has emerged as an important mechanism in cancer progression by influencing oncogenic translation. Anaplastic thyroid carcinoma (ATC) is a highly aggressive malignancy with limited therapeutic options. Although *N*-acetyltransferase 10 (NAT10) is frequently overexpressed in multiple cancers, its functional role and therapeutic potential in ATC remain unclear.

**Methods:**

We employed integrated approaches including bioinformatics analyses,in vitro and in vivo assays, multi-omics profiling (mRNA-seq, Ribo-seq, tRNA RedaC-seq), and mechanistic studies (ChIP, LC–MS and ubiquitination assays) in ATC cell lines and xenograft models.

**Results:**

NAT10 is significantly upregulated in ATC and correlates with poor prognosis. Functional assay demonstrates that NAT10 enhances ATC cell proliferation and invasion in vitro and in vivo. The targeted inhibition of NAT10 using the small molecule inhibitor remodelin effectively suppresses ATC cell growth. Mechanistically, NAT10 forms a positive feedback loop with the transcription factor c-Myc: c-Myc transcriptionally activates NAT10, whereas NAT10 is associated with enhanced translation of c-Myc in conjunction with tRNA ac^4^C modification. NAT10 depletion reduces global translation efficiency, accompanied by decreased tRNA ac^4^C levels, and also affects c-Myc protein stability via the deubiquitinase OTUB1. Moreover, combined treatment with remodelin and doxorubicin exhibits synergistic antitumor effects in both in vitro and in vivo models.

**Conclusions:**

These findings identify a NAT10/c-Myc positive feedback loop associated with tRNA ac^4^C modification and protein stabilization in ATC. Targeting this regulatory axis with remodelin in combination with doxorubicin may represent a promising therapeutic strategy.

**Graphical Abstract:**

**Figure:**
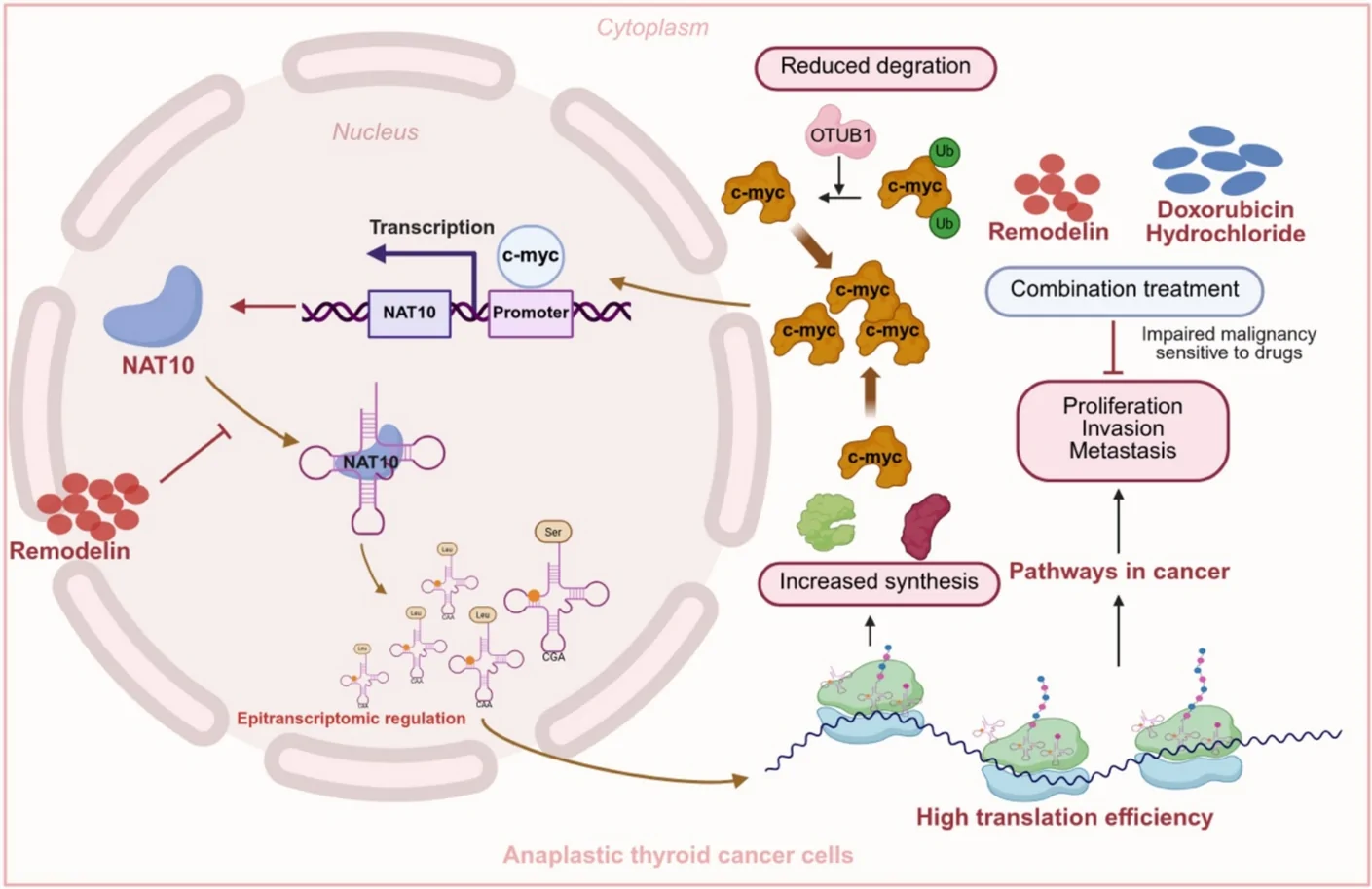

NAT10 promotes a c-Myc positive feedback loop via tRNA ac⁴C modification and OTUB1-mediated stabilization, driving ATC progression.

**Supplementary Information:**

The online version contains supplementary material available at https://doi.org/10.1186/s11658-026-00977-x.

## Introduction

Anaplastic thyroid carcinoma (ATC), a highly refractory subtype of thyroid malignancy, is characterized by aggressive invasion, rapid progression, and an extremely poor prognosis, with a median survival of less than 6 months [1]. ATC typically presents insidiously, and most patients are diagnosed with local invasion, cervical lymph node metastasis, and distant metastasis [2, 3]. Once diagnosed, complete surgical resection is often unachievable [4]. Moreover, ATC demonstrates resistance to both radiotherapy and chemotherapy, resulting in poor overall treatment outcomes [5]. Currently, no effective therapies exist for ATC. Therefore, elucidating the molecular mechanisms driving ATC’s rapid progression and developing targeted therapeutics are urgently needed to improve patient outcomes.

Current cancer research intensively investigates posttranscriptional modifications as pivotal regulators of the transcriptome. Among these, transfer RNA (tRNA) modifications have emerged as master orchestrators of translational reprogramming, empowering cancer cells to rapidly adapt to oncogenic and environmental stressors [6, 7]. This paradigm fundamentally redefines tRNA’s role beyond that of a mere genetic code adaptor. However, tRNA’s core function in translation remains undisputed, and its genes were historically regarded as ubiquitous “housekeeping” elements [8]. Recent findings reveal tissue-specific expression patterns and critical regulatory roles mediated by dynamic tRNA modifications [9]. Diverse chemical alterations-including m^1^A, m^5^C, and m^7^G-precisely modulate tRNA stability, structural integrity, decoding fidelity, and functional capacity [10, 11]. Critically, tRNA modification-driven reprogramming of mRNA translation operates through a codon-dependent mechanism, directly driving tumorigenesis, metastasis, and therapy resistance [12, 13].

Among the key tRNA posttranscriptional modifications, *N*^4^-acetylcytidine (ac^4^C) modification is garnering significant attention. This modification occurs primarily at specific cytidine residues on tRNA and is catalyzed by the enzyme *N*-acetyltransferase 10 (NAT10) [14, 15]. Research indicates that NAT10, by mediating ac^4^C modification of tRNA, influences tRNA structural stability and nucleocytoplasmic transport efficiency, thereby regulating the translation rates of oncoproteins [16]. Importantly, dysregulated NAT10 expression has been linked to poor patient prognosis in solid tumors such as colorectal cancer [17] and bladder cancer [18]. However, the molecular mechanisms by which NAT10 drives ATC progression remain to be elucidated.

Strikingly, the proteomic landscape driving cancer progression is not solely determined by de novo translation; it is equally governed by targeted protein degradation mediated by the ubiquitin–proteasome system (UPS) [19]. While tRNA ac^4^C modifications fine-tune oncoprotein synthesis, ubiquitination orchestrates their precise degradation. Beyond its well-established role in catalyzing ac^4^C tRNA modifications to promote oncogenic translation [20], NAT10 also emerges as a regulator of ubiquitin-dependent pathways [21, 22]. This functional duality positions NAT10 as a critical balancer of protein production and destruction-a dynamic equilibrium essential for cellular homeostasis.

Herein, we delineate the convergence of NAT10-driven ac^4^C epitranscriptomics and ubiquitin-proteolytic regulation in amplifying the proto-oncogene c-Myc in ATC. We demonstrate that NAT10 promotes c-Myc synthesis through a mechanism associated with tRNA ac4C-dependent codon usage while simultaneously stabilizing c-Myc through OTUB1-mediated deubiquitination, thereby establishing a self-reinforcing oncogenic circuit. The pharmacological inhibition of NAT10 abrogates this dual regulatory axis, suppressing malignant phenotypes and restoring chemosensitivity in ATC. These findings establish NAT10 as a central regulator of proteostatic control in ATC and identify its targeting as a promising synergistic therapeutic vulnerability.

## Method and materials

### Patient samples

All ATC and normal thyroid tissue specimens were obtained from Xiangya Hospital, Central South University. Paraffin-embedded ATC samples were utilized in accordance with institutional review board (IRB)-approved protocols. The study was approved by the Xiangya Hospital Protection of Human Subjects Committee and written informed consent was obtained from all patients.

### Bioinformatics analysis

Bioinformatics analyses were performed using R (version 4.2.1). Four gene expression datasets were obtained from the GEO database via the ‘GEOquery’ package: GSE76039 (17 poorly-differentiated thyroid cancer (PDTC) and 20 ATC), GSE65144 (13 normal thyroid tissues and 12 ATC), GSE29265 (20 normal thyroid tissues, 20 papillary thyroid cancer (PTC), and 9 ATC), and GSE33630 (45 normal thyroid tissues, 49 PTC, and 11 ATC). Batch effects were corrected using the ComBat function from the ‘sva’ package. All datasets were subsequently renormalized with the normalize Between Arrays function from the ‘limma’ package. Differential expression analysis between experimental groups was conducted using the ‘limma’ package. Data visualizations were implemented in R.

### Cell culture

Human thyroid cell lines (Nthy-ori-3-1, FRO, 8305C, KHM-5 M) from ATCC were authenticated by STR profiling. Cells were maintained at 37 °C/5% CO₂ in a humidified incubator (Thermo Scientific), cultured in DMEM or RPMI-1640 (Gibco) supplemented with 10% FBS and 1% penicillin–streptomycin (both Gibco), with media type matched to cell line requirements.

### Plasmid construction, lentivirus production, and cell transduction

Lentiviral shRNA plasmids targeting NAT10 were designed, synthesized, annealed, and cloned by General Biol (Anhui, China). Corresponding shRNA sequences are provided in Supplementary Table S1. Expression plasmids for human NAT10, NAT10 G641E mutant, and OTUB1 were synthesized by YouBio (Shanghai, China, Supplementary Table S1). c-Myc-targeting siRNAs were commercially sourced (General Biol). All oligonucleotide sequences are detailed in Supplementary Table S1.

For lentivirus conduction, psPAX2 and pMD2.G plasmid (IGE biotechnology) were transfected into HEK-293 T using Lipofectamine 3000 (Invitrogen). Viral supernatants were harvested 48–72 h post transfection. Target tumor cells were seeded in six-well plates (5 × 10^4^ cells/well) 24 h prior to transduction. Lentiviral transduction was performed at an MOI of 10 in medium containing 8 μg/mL polybrene (Genechem, Shanghai, China).

### Immunohistochemical (IHC) and immunofluorescence (IF) staining

Tissue specimens were fixed in 10% neutral-buffered formalin, paraffin-embedded, and sectioned at 4 μm. After deparaffinization and hydration, antigen retrieval was performed in citrate buffer. For IHC, sections were incubated overnight at 4 °C with primary antibodies followed by PV-6000 secondary antibodies (ZSGB-BIO, 1 h, RT), developed with DAB and counterstained with hematoxylin. IHC images were quantified via average optical density (AOD) analysis using ImageJ (v1.53e) within standardized IHS ranges. For IF, cells on coverslips were fixed with 4% PFA (15 min), permeabilized with 0.25% Triton X-100 (10 min), and blocked with 3% BSA (30 min); tissue sections underwent analogous processing with optimized blocking. All samples were incubated overnight at 4 °C with primary antibodies (anti-NAT10: 13365–1-AP, Proteintech, 1:200; anti-MYC: 67447–1-Ig, Proteintech, 1:200. Supplementary Table S2), then detected using Alexa Fluor^®^ 596-conjugated secondary antibodies (no. 8889, CST). Images were acquired on a DMi8 fluorescence microscope (Leica).

### Quantitative reverse-transcription PCR (qRT–PCR)

Total RNA was extracted using TransZol Up Reagent (ET111, TransGen) and reverse-transcribed with Evo M-MLV RT Mix (AG11728, Accurate Biology). cDNA was diluted 1:5 in nuclease-free water and amplified using SYBR Green Premix Pro Taq HS (AG11718, Accurate) on a QuantStudio™ 5 system (Applied Biosystems). Reactions (20 μL) contained 2 μL cDNA template with cycling conditions: 95 °C for 30 s; 40 cycles of 95 °C for 5 s and 60 °C for 30 s. Samples were run in triplicate with GAPDH normalization; primer sequences are in Supplementary Table S1.

### Western blotting

Cells were lysed in radioimmunoprecipitation assay (RIPA) buffer (WB3100, NCM Biotech) containing protease inhibitors. Protein concentrations were determined by BCA assay (WellBio). Lysates (20 μg/lane) were separated by 10% sodium dodecyl sulfate–polyacrylamide gel electrophoresis (SDS–PAGE) and transferred to polyvinylidene fluoride (PVDF) membranes (Millipore). Membranes were blocked for 20 min at RT and incubated overnight at 4 °C with primary antibodies (Supplementary Table S2). After incubation with horseradish peroxidase (HRP)-conjugated secondary antibodies, proteins were detected using NcmECL substrate (NCM Biotech) and quantified with an ImageQuant™ 800 system (Cytiva). GAPDH and β-actin served as loading controls.

### Cell proliferation, migration and invasion assays

Cell proliferation was assessed via CCK-8 assay (Beyotime, China) and colony formation. For CCK-8, cells (2–3 × 10^3^/well) in 96-well plates were measured daily over 5 days. Colony assays used 300–500 cells/well in six-well plates cultured for 2 weeks; colonies were fixed with 4% PFA, stained with 0.5% crystal violet, and quantified using ImageJ (v1.53e). Migration and invasion were evaluated using Transwell chambers. For invasion assays, chambers were precoated with Matrigel. Cells (1–3 × 10^4^/well) in serum-free medium were seeded in the upper chamber, with 600 μl of 10% fetal bovine serum (FBS)-medium in the lower chamber. After 24–48 h incubation, traversed cells on the membrane underside were fixed, stained, and imaged.

### Animal studies

All animal procedures were approved by the Institutional Animal Care and Use Committee of Xiangya Hospital, Central South University. Female BALB/c nude mice (6–8 weeks old) were purchased from SJA Laboratory Animal Co. and housed under specific pathogen-free (SPF) conditions.

Subcutaneous Xenograft Model. KHM-5 M cells (5 × 10^6^ in 100 μL PBS) were injected subcutaneously into the right dorsal flank. Tumor dimensions were measured every 3 days using calipers, and volume was calculated as *V* = (*W*^2^ × *L*)/2 (*W* = width; *L* = length). Mice were euthanized at the experimental endpoint (tumor volume ≥ 1500 mm^3^ or day 21 post injection).

Lung metastasis model: NAT10-knockdown or control KHM-5 M cells (1 × 10^6^ in 200 μL PBS) were injected intravenously via the tail vein. Mice were monitored for 6–8 weeks before sacrifice and lung collection for metastasis analysis.

Pharmacological intervention in xenografts: when established KHM-5 M xenografts reached ~100 mm^3^, mice received intraperitoneal injections every 72 h of either 4 mg/kg doxorubicin (HY-15142; MedChemExpress, USA), 20 mg/kg remodelin (HY-16706; MedChemExpress, USA), or vehicle control (0.9% saline). Tumors were harvested 15 days after treatment initiation.

### Anti-cancer drugs inhibition assay

The half-maximal inhibitory concentration (IC_50_) of doxorubicin was assessed using a CCK-8 assay. Cells (3 × 10^3^ cells/well) were seeded in 96-well plates in 100 μL Dulbecco’s modified Eagle medium (DMEM) supplemented with 10% FBS. After 24 h of treatment with serial dilutions of doxorubicin (eight concentrations), 10 μL of CCK-8 solution was added per well. Following a 2 h incubation at 37 °C, absorbance at 450 nm was measured (NanoQuant Infinite M200 PRO). Cell viability percentages were calculated, and IC_50_ values were determined using nonlinear regression in GraphPad Prism (v9.4.1). Time-dependent cytotoxicity was assessed at concentrations bracketing the IC_50_.

### Puromycin intake assay

Nascent protein synthesis was monitored using puromycin immunodetection. Following NAT10 knockdown (small interfering RNAS (siRNAs); Supplementary Table S1), cells were pulsed with 10 μg/mL puromycin for 20 min (37 °C, 5% CO_2_). For the cycloheximide group, cells were pretreated with 100 μg/mL cycloheximide (CHX; HY-12320, MCE) for 20 min prior to puromycin exposure. Cells were immediately lysed for immunoblotting using anti-puromycin antibody (A23031, Abclonal) to detect nascent polypeptide chains.

### Dot blotting

Total RNA from per sample was denatured at 95 °C for 3 min, snap-cooled, and spotted onto nylon membranes (Millipore INYC00010, 0.45 μm). After ultraviolet (UV) crosslinking, membranes were blocked and probed with anti-ac^4^C antibody (A18806, Abclonal) overnight at 4 °C. HRP-conjugated secondary antibody was applied (1 h, 25 °C), and signals were developed using chemiluminescence (NcmECL Ultra Reagent; P10300) on an ImageQuant 800 system. RNA loading was verified by methylene blue staining and visualized using Biorad ChemiDoc system.

### Dual-luciferase reporter assay

All plasmids were designed and constructed by YouBio (Shanghai, China). The plasmids and renilla luciferase reporter vector were cotransfected into cells with Lipofectamine 3000 (Thermo Fisher) for 36 h. Luciferase activities were measured using the dual-luciferase reporter assay system (Promega), with both firefly and renilla luciferase activities assessed in each well. The final results were expressed as the ratio of firefly luciferase activity to renilla luciferase activity.

### Chromatin immunoprecipitation-PCR assay

Chromatin immunoprecipitation (ChIP) assay was performed using the ChIP assay kit (ab500, Abcam, USA) according to the manufacturer’s instructions. In brief, cells were cross-linked with 1.1% formaldehyde for 10 min at 37 °C and the chromatin was sonicated to generate DNA fragments. Antibodies of c-Myc (IgG as the control) were utilized to precipitate the cross-linked protein–DNA complexes. The chromatin DNA was extracted and the samples were subjected to quantitative PCR analysis with specific primers. The primer sequences are listed in Supplementary Supplementary Table S1.

### RNA sequencing

RNA sequencing (RNA-seq) was performed on NAT10-knockdown cells to identify NAT10-regulated mRNAs, following standard protocols. Sequencing services were provided by Aksomics Inc. (Shanghai, China).

$${Translation \,efficiency}=\frac{\text{Normalised translation level}}{\text{Normalised mRNA level}}$$

### Ribosome sequencing

Ribosome profiling was performed as follows: cells were pretreated with 100 µg/mL CHX for 10 min prior to harvesting. Cell pellets were flash-frozen in liquid nitrogen and stored at −80 °C. Ribosome-protected fragments (RPFs) were prepared according to a modified published protocol. Ribosome profiling sequencing services were provided by Aksomics Inc. (Shanghai, China).

### RedaC:T-seq (ac^4^C)-tRNA sequencing

Ac^4^C RedaC:T-seq was performed by Cloud-Seq Biotech (Shanghai, China). Total RNA was extracted, and small RNA (< 200 nt) was isolated using the miRNeasy Mini Kit (Qiagen). The purified small RNA was split: one portion was reduced with NaBH4 (converting ac^4^C to tetrahydroac^4^C), while the other served as an untreated control. Libraries for both groups were constructed using the GenSeq^®^ Small RNA Library Prep Kit (GenSeq, Inc.) following the manufacturer’s protocol. Library quality was assessed using the BioAnalyzer 2100 system (Agilent Technologies), followed by high-throughput sequencing.

### tRNA sequencing

tRNA sequencing was performed by CloudSeq Inc. (Shanghai, China). Total RNA was fractionated using the mirVana™ miRNA Isolation Kit (Thermo Fisher) to enrich small RNAs (< 200 nt), followed by deacylation in 100 mM Tris–HCl (pH 9.0) containing 1 mM EDTA (37 °C, 30 min) to remove amino acid moieties. tRNA libraries were constructed using the GenSeq^®^ Small RNA Library Prep Kit (GenSeq Inc.) according to manufacturer specifications, incorporating adapter ligation and complementary DNA (cDNA) synthesis steps. Size selection targeting mature tRNAs (70–90 nt) was performed prior to amplification. Final libraries were quantified by Qubit and quality-controlled via BioAnalyzer, then subjected to 150-bp paired-end sequencing on an Illumina NovaSeq 6000 platform (San Diego, CA, USA) with a minimum depth of 20 million reads per sample.

### LC–MS analysis

Liquid chromatography-mass spectrometry (LC–MS), the gold-standard technique for the quantitative profiling of tRNA ac^4^C modification levels, was utilized to validate the results obtained from ac^4^C-RedaC:T-seq and dot blot analyses. tRNA isolation and LC–MS analysis were performed by Aksomics Inc. (Shanghai, China). Total RNA was processed using the NEBNext Poly(A) tRNA Magnetic Isolation Module (NEB, E7490), with purified tRNA quantified via Qubit RNA HS Assay Kit (Thermo Fisher, Q32855). Isolated tRNA underwent: hydrolysis to nucleosides; dephosphorylation with enzyme cocktail; and deproteinization using 10-kDa MWCO filters (Sartorius). Nucleoside mixtures were separated on an Agilent 1260 HPLC system coupled to a 6460 QQQ mass spectrometer. Data acquisition used Agilent Qualitative Analysis software, with modified nucleosides quantified via multireaction monitoring (MRM). Peak intensities were normalized to input tRNA quantities.

### Molecular docking prediction

To investigate the interaction between OTUB1 and c-Myc proteins, we downloaded the corresponding protein crystal diffraction structures in PDB format from the PDB BANK. Using Python, we performed sequence reorganization and removed non-standard residues from the PDB files. Initial docking conformations were generated using ClusPro and HADDOCK, based on hotspot formation. Under the constraints of these initial conformations, a config file was created for batch docking using AutoDock Vina. Finally, molecular docking visualization was carried out using ChimeraX.

### Codon usage bias analysis

Codon usage bias analysis was systematically performed to investigate translational regulation mediated by NAT10-dependent tRNA ac^4^C modifications in ATC. Protein-coding sequences (CDS) of translationally dysregulated genes were extracted and processed using CodonW 1.4.2 (http://codonw.sourceforge.net/). Key metrics—including relative synonymous codon usage (RSCU), effective number of codons (ENC), and GC content at the third synonymous position (GC3s)—were calculated to quantify codon preference heterogeneity. RSCU values > 1 indicated codon overrepresentation, while ENC (range: 20–61) measured bias intensity, with lower values signifying stronger deviation from random usage. ENC-GC3s scatter plots further assessed evolutionary pressures, wherein genes below the theoretical neutral curve [ENC = 2 + GC3s + 29/(GC3s^2^ + (1 − GC3s)^2^)] exhibited selection for translational optimization.

### Statistical analysis

All statistical analyses were performed using GraphPad Prism (version 9.4.1), R (version 4.2.1) and Python (version 3.9.1). Band intensities were quantified by densitometric analysis using ImageJ software. Pearson’s correlation test analyzed linear relationships between continuous variables. Unpaired two-tailed Student’s *t*-tests determined the significance of in vitro (proliferation, migration, colony formation) and in vivo (tumor volume/weight, metastasis) experimental data. Survival analysis was conducted using the Kaplan–Meier method with log-rank tests. The synergistic effect of drug combinations was evaluated by the Chou-Talalay method, and the combination index (CI) was calculated using CompuSyn software. *p*-values < 0.05 were considered statistically significant for all analyses.

## Result

### NAT10 is upregulated in ATC and associated with poor prognosis

**Fig. 1:**
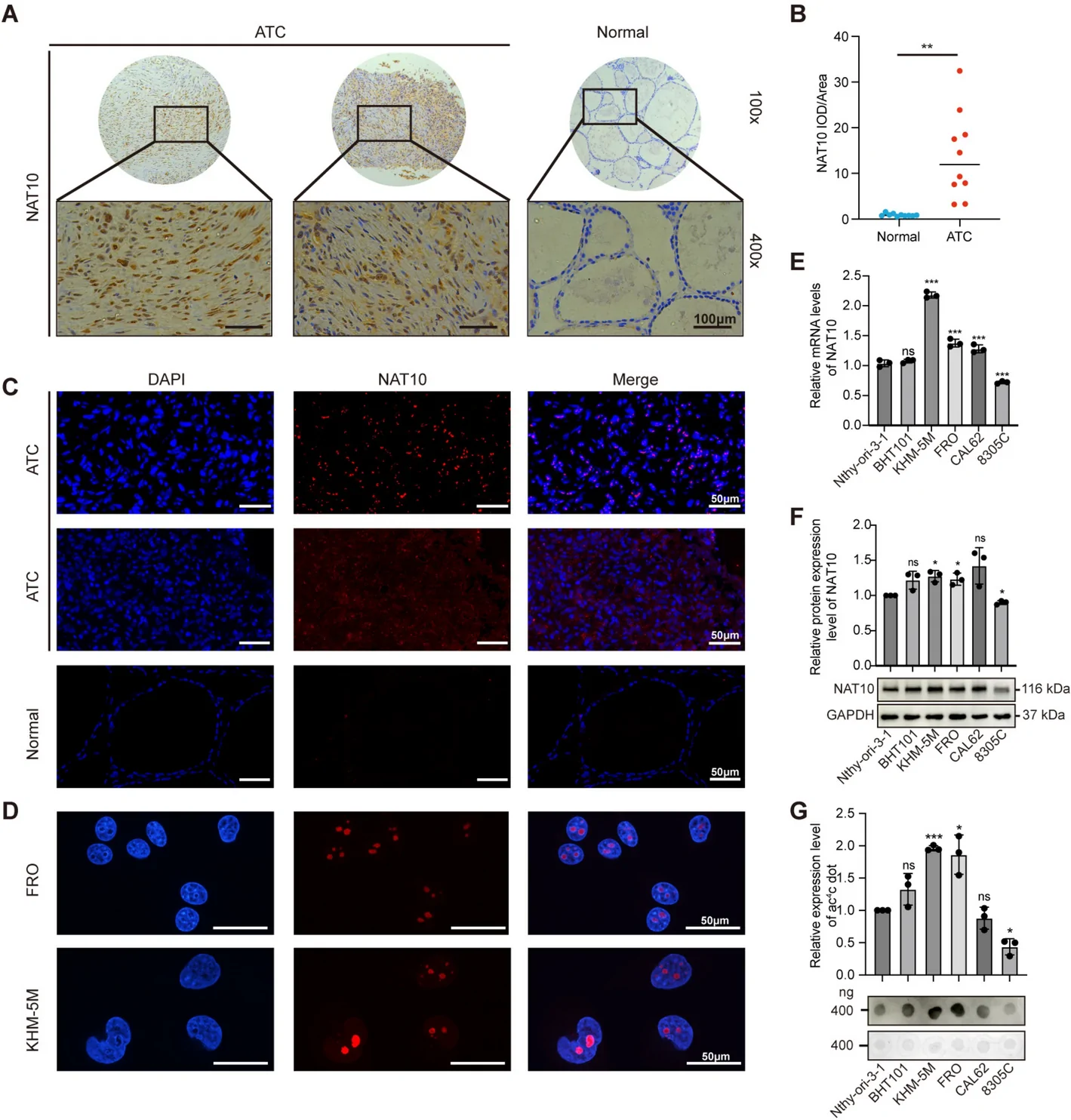
NAT10 plays a potential oncogenic role in ATC. **A** Representative images and **B** quantitative analysis of IHC staining showing elevated NAT10 expression in tumor tissues (*n* = 10). **C**–**D** Representative images IF staining of NAT10 showed NAT10 located in nuclear. qRT–PCR **E** and western blot **F** showing that NAT10 mRNA and protein levels are higher in ATC cell lines than in the normal thyroid Nthy-ori-3-1 cell line (*n* = 3). **G** Dot blotting show that the total ac^4^C levels in acetylated RNA in ATC cell lines were higher than normal thyroid Nthy-ori-3-1 cell line. *p < 0.05, **p < 0.01, and ***p < 0.001. Abbreviations: kDa, kilodalton

To investigate the potential role of NAT10 in ATC, we analyzed NAT10 expression in GEO database using bioinformatics methods. Results showed that NAT10 expression was significantly upregulated in ATC tissues compared with normal thyroid tissues (Supplementary Fig. S1A). Notably, NAT10 expression was also significantly higher in ATC tissues than in other types of thyroid cancer (such as papillary thyroid cancer and poorly differentiated thyroid carcinoma) (Supplementary Fig. S1A). IHC analysis confirmed higher NAT10 expression in ATC tissues compared with adjacent tissues (Fig. 1A, B). Immunofluorescence further revealed significant NAT10 overexpression in ATC tissues **(**Fig. 1C). As anticipated, high NAT10 expression correlated with poorer prognosis in patients with thyroid cancer (Supplementary Fig. S1B). Immunofluorescence also indicated that NAT10 was primarily localized to the nuclei of ATC cells (Fig. 1D). Quantitative reverse-transcription polymerase chain reaction (RT–qPCR) and western blot (WB) experiments confirmed that both mRNA and protein expression levels of NAT10 were higher in ATC cells than in normal thyroid cells (Fig. 1E, F). The dot blot also demonstrated that the ac^4^C modification level was significantly higher in ATC cells than in normal thyroid cells (Fig. 1G). These data suggest that NAT10 may play a potential oncogenic role in ATC.

### NAT10 promotes ATC cell proliferation and metastasis in vitro and in vivo

**Fig. 2:**
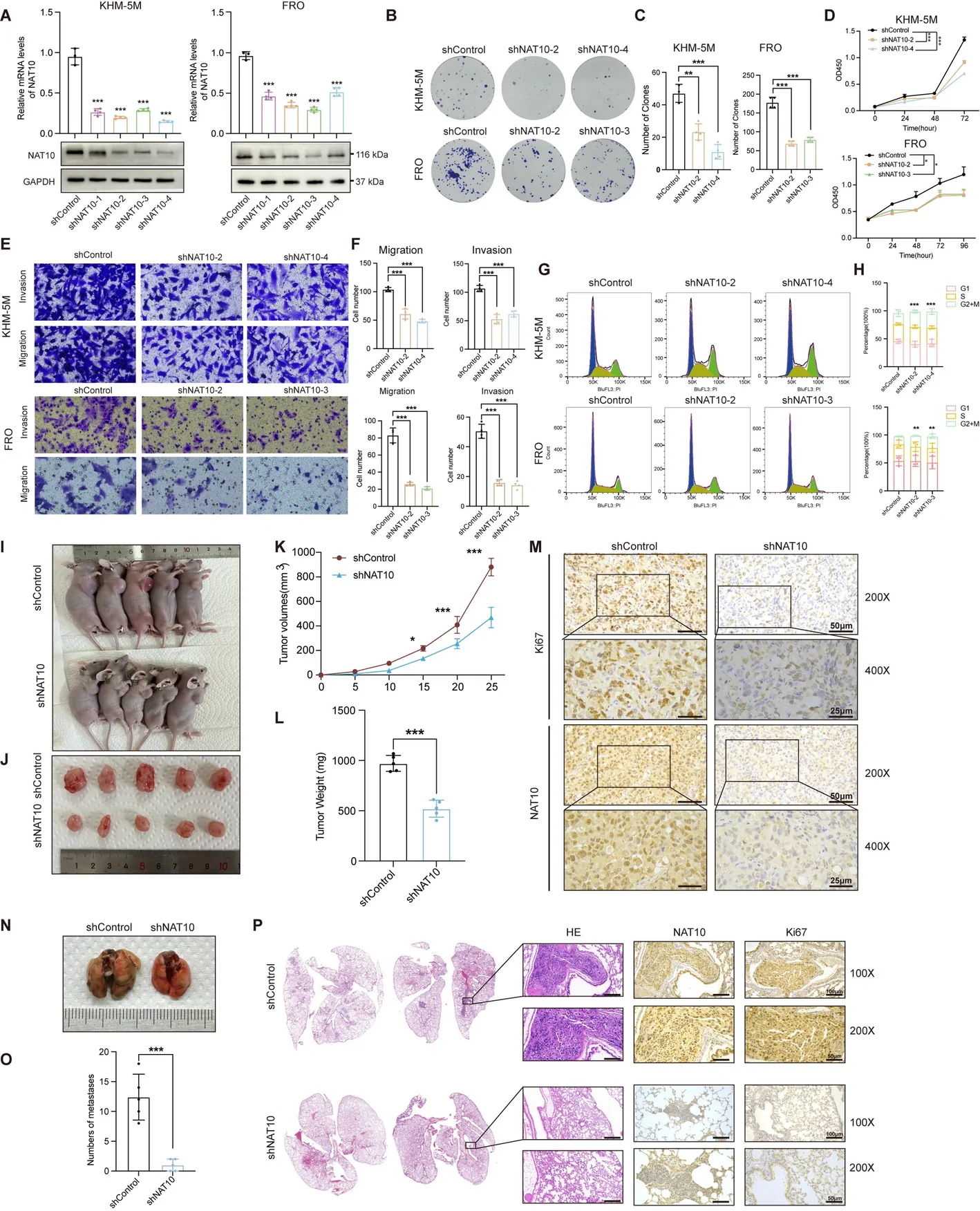
NAT10 knockdown inhibits ATC cell proliferation, invasion and metastasis in vitro and in vivo. **A** Knockdown of NAT10 at both the mRNA and protein levels was confirmed by qRT–PCR and western blotting. **B**–**D** NAT10 knockdown suppressed cell proliferation, as assessed by colony formation assay (**B**, quantified in **C**) and CCK-8 assay **D**. **E**–**F** NAT10 knockdown impaired cell invasion and migration by Transwell assay (quantified in F). **G**–**H** Flow cytometry showed that knockdown of NAT10 induced G2/M phase arrest in ATC cells (quantified in** H**). **I**–**J** Overview of tumors in xenograft mice model subcutaneously implanted with shControl and shNAT10 cells. **K** The growth curves showing ATC cell proliferation in vivo. **L** The bar graph shows the tumor weights of different groups on the 25th day after cell inoculation. **M** HE and IHC staining of NAT10 and Ki67 in tumor samples in xenograft mice model subcutaneously implanted with shNAT10 and shControl cells. **N** Representative images of tumors in the mice lung metastasis model via tail vein injection of shControl and shNAT10 cells. **O** The bar graph shows the number of pulmonary metastatic nodules in mice from the shControl and shNAT10 groups. **P** HE and IHC staining of lung-metastatic nodules in model mice injected via tail vein with shControl and shNAT10 cells. *p < 0.05, **p < 0.01, and ***p < 0.001

To assess the functional impact of NAT10 on ATC progression, we performed loss-of-function assays using two NAT10-high-expressing ATC cell lines, KHM-5 M and FRO (Fig. 2A, S1C. The NAT10 knockdown significantly impaired the proliferation and colony-formation capacities of both KHM-5 M and FRO cells (Fig. 2B, D). Similarly, Transwell assays demonstrated that inhibiting NAT10 significantly reduced the migration and invasion ability of these cells (Fig. 2E, F). Flow cytometric cell cycle analysis revealed that the proportion of cells in G2/M phase was significantly increased following NAT10 knockdown, suggesting that the downregulation of NAT10 induces G2/M phase arrest and thereby inhibits cell proliferation (Fig. 2G, H).

To determine whether NAT10-promoted malignant phenotypes depend on its ac^4^C acetyltransferase activity, we used a low-NAT10-expressing 8305C cell model and reintroduced either wild-type NAT10 or a catalytically inactive mutant (NAT10-G641E) lacking acetyltransferase activity (Supplementary Fig. S1D, S1E). Overexpression of WT-NAT10 significantly enhanced proliferation, migration, and colony formation capacity, whereas the G641E mutant had minimal effects (Supplementary Fig. S1F–S1H). These results establish that these results suggest that NAT10 drives ATC progression in a manner dependent on its acetyltransferase activity and associated with ac4C modification.

To ascertain whether NAT10-induced proliferation of ATC cells in vitro confers tumor growth capacity in vivo, we established subcutaneous xenograft and lung metastasis models in nude mice using KHM-5 M cells with stable NAT10 silencing. NAT10 knockdown effectively inhibited ATC tumor growth (Fig. 2I, J), evidenced by significantly reduced tumor volume (Fig. 2K) and weight (Fig. 2L) compared with the control group. Similarly, NAT10 knockdown significantly reduced the number of lung metastatic foci (Fig. 2M–O). IHC staining revealed lower expression of NAT10 and decreased levels of the proliferation marker Ki67 in tumors from shNAT10 mice (Fig. 2M, P), suggesting that NAT10 knockdown reduces the proliferation capacity of ATC. Overall, these data indicate that NAT10 plays a vital role in promoting the growth and metastasis of ATC cells.

### Remodelin induces growth arrest in ATC cells

Given that NAT10 represents a promising therapeutic target for ATC, we explored therapeutic strategies targeting NAT10. Remodelin, an effective small-molecule inhibitor of NAT10, was used to assess its inhibitory effect on ATC. Remodelin treatment downregulated inhibited ATC cell proliferation in a dose-dependent manner (Supplementary Fig. S2A) and significantly reduced the colony-formation capacity of KHM-5 M and FRO cells (Supplementary Fig. S2B, S2C). Remodelin treatment also significantly decreased the migration and invasion capacities of these cells (Supplementary Fig. S2D, S2E). These results demonstrate that the NAT10 inhibitor remodelin can effectively block ATC progression.

### The transcription factor c-Myc upregulates NAT10 in ATC

**Fig. 3:**
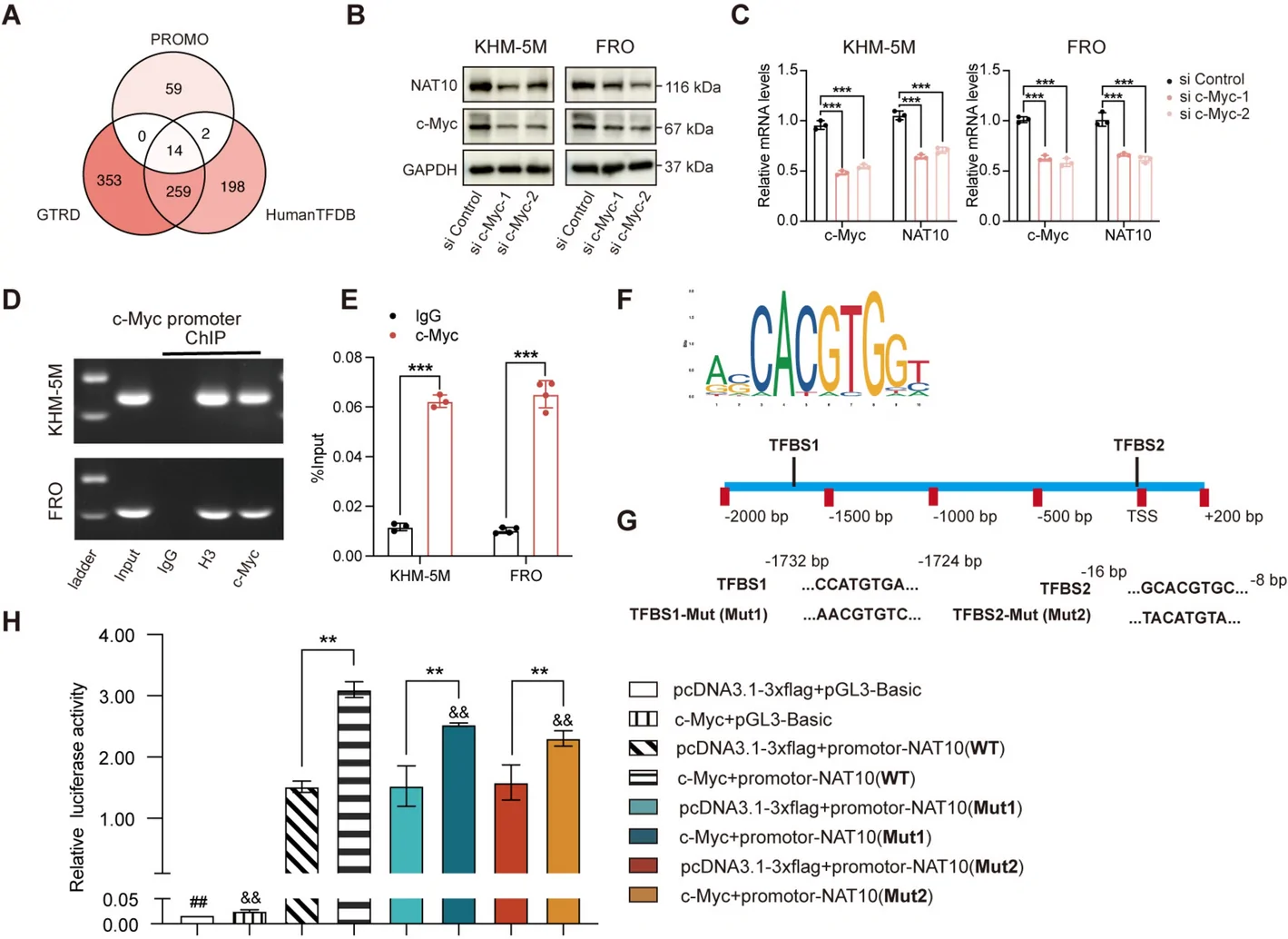
NAT10 is directly regulated by the transcription factor c-Myc. **A** Bioinformatic analysis identifies 14 genes as potential transcriptional drivers of NAT10 expression. **B**–**C** Knockdown of c-Myc significantly decreased both the mRNA **B** and protein **C** levels of NAT10. **D**–**E** Chromatin immunoprecipitation coupled with qRT–PCR confirms c-Myc binding to the NAT10 promoter region. **F** JASPAR-predicted c-Myc binding sites within the NAT10 promoter region. **G** Schematic of potential c-Myc binding sites and corresponding mutations in the NAT10 promoter. **H** Dual-luciferase reporter assays of NAT10 promoter activity (WT, Mut1, Mut2). ## *p* < 0.01 compare with NAT10(WT) + pcDNA3.1–3xflag; &&P < 0.01 compare with NAT10(WT) + c-Myc; ** *p* < 0.01 compare with NAT10(WT) + pcDNA3.1–3xflag or NAT10(Mut1) + pcDNA3.1–3xflag, or NAT10(Mut2) + pcDNA3.1–3xflag. Data: mean ± standard deviation (SD); *n* = 3, *p < 0.05, **p < 0.01, and ***p < 0.001

To investigate the mechanism underlying high NAT10 expression in ATC, we systematically screened potential NAT10-regulating transcription factors (TFs) using PROMO, Human TFDB, and GTRD databases. Venn diagram analysis identified fourteen candidate TFs (Fig. 3A). Correlation analysis across four independent ATC datasets (GSE65144, GSE33630, GSE29265, GSE76039; Supplementary Fig. S3A) revealed significant positive associations (r_pearson_ > 0.5, *p* < 0.05) only between NAT10 and c-MYC/RELA. Subsequent qRT–PCR and western blotting demonstrated that c-Myc knockdown substantially reduced NAT10 expression at both mRNA and protein levels (Fig. 3B, C, Supplementary Fig. S3D), whereas RELA knockdown showed no significant effect (Supplementary Fig. S3B, S3C). Furthermore, c-Myc depletion significantly attenuated NAT10-mediated ac^4^C modification (Supplementary Fig. S3E, S3F), indicating c-Myc acts as an upstream regulator of NAT10.

ChIP assays confirmed that c-Myc directly binds to the NAT10 promoter region (Fig. 3D, E). JASPAR predictions identified two potential c-Myc binding motifs within the NAT10 promoter (Fig. 3F). We constructed wild-type and mutant luciferase vectors on the basis of these predicted binding sites (BS) (Fig. 3G). Mutation of either TFBS1 or TFBS2 significantly reduced luciferase activity (Fig. 3H). These data indicate that c-Myc serves as a transcription factor regulating NAT10 expression levels.

### NAT10 depletion reduces tRNA ac^4^C modification and overall mRNA translation

**Fig. 4:**
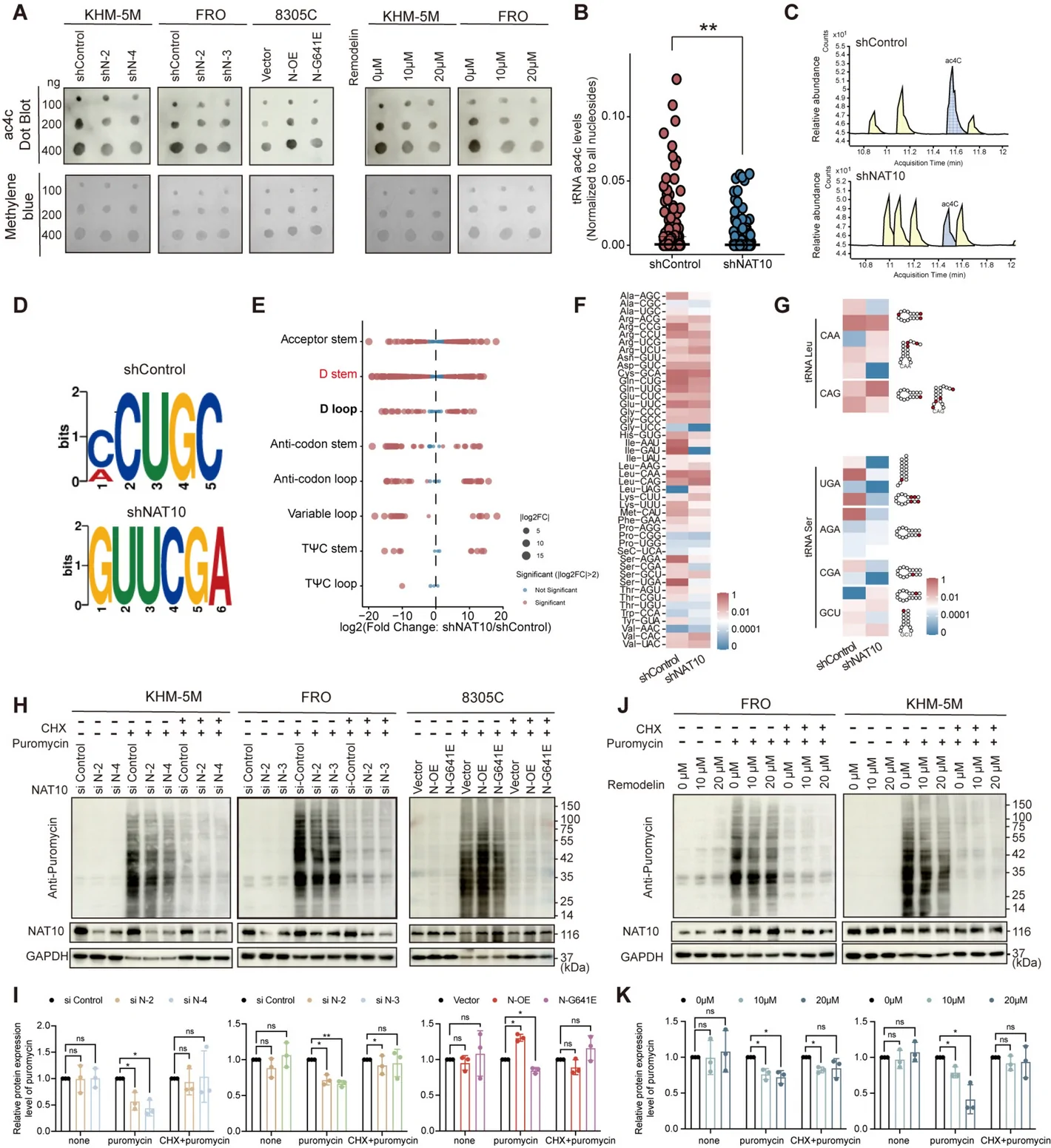
NAT10 remodels cancer-relevant pathways through translatomic alterations. **A** Dot blotting show a positive correlation between NAT10 levels and ac^4^C acetylation modification of RNA in ATC cells and remodelin can suppress the ac^4^C modification levels of RNA in ATC cells. **B**–**C** Total tRNA ac^4^C quantification in shControl versus shNAT10 cells by RedaC:T-seq **B** and LC–MS **C**. **D** Motif enrichment analysis of ac^4^C-modified sites. **E** ac^4^C rate density mapping on tRNA secondary structures. Each point represents site-specific modification. **F** Heatmap of ac^4^C levels across cytoplasmic tRNA decodons. Color intensity: relative modification change. **G** Site-specific ac^4^C modification rates (RedaC:T-seq). **H**–**I** Positive correlation between NAT10 expression and global protein synthesis rates in ATC cells. **J**–**K** Remodelin suppressed protein synthesis in ATC cells by puromycin intake assay. si N: si NAT10; shN: shNAT10, N-OE: wild-type NAT10 overexpression, N-G641E: NAT10-G641E overexpression. Data: mean ± SD; *n* = 3, *p < 0.05, **p < 0.01, and ***p < 0.001

To investigate the molecular mechanisms by which NAT10 regulates ATC progression, we initially performed dot blot analysis to assess ac^4^C modification levels. Strikingly, NAT10 knockdown in both KHM-5 M and FRO cell lines resulted in a marked reduction of ac^4^C modification levels. Consistently, ectopic overexpression of wild-type NAT10 substantially elevated global ac^4^C modification, whereas overexpression of NAT10-G641E exhibited no effect on ac^4^C levels (Fig. 4A, S4A). Next, we performed RedaC:T-seq (ac^4^C) tRNA sequencing to profile the ac^4^C modification landscape of tRNAs following NAT10 knockdown. As shown in Fig. 4B, NAT10 depletion led to a marked reduction in ac^4^C levels across the majority of tRNA species. Importantly, LC–MS, the gold-standard method for quantifying tRNA ac^4^C modification levels, further validated that NAT10 knockdown significantly decreased the global ac^4^C abundance in tRNAs (Fig. 4C). Motif analysis further identified ac^4^C-enriched sites within tRNA sequences (Fig. 4D). These modifications mainly occurred on the D stem (Fig. 4E). We further verified the changes in tRNA expression levels after NAT10 knockdown and found that the ac^4^C levels of 46 tRNAs were found to be significantly downregulated (Fig. 4F). Meanwhile, the ac^4^C modification level of tRNA-Leu and tRNA-Ser, which we focused on, showed a significant decrease in the NAT10 knockdown ATC cells (Fig. 4G). Collectively, these data demonstrate that NAT10 knockdown leads to a widespread reduction in ac^4^C modification levels across multiple tRNA species in ATC cells. Among the affected tRNAs, tRNA-Leu and tRNA-Ser were identified as significantly downregulated, prompting further investigation into their potential functional involvement in ATC progression.

As tRNAs are essential for mRNA translation, we next investigated whether tRNA ac^4^C modification regulates mRNA translation in ATC cells. Puromycin uptake assays indicated that knockdown of NAT10 significantly reduced overall protein synthesis in the KHM-5 M and FRO cells (Fig. 4H, I, S4C). In contrast, overexpression of wild-type NAT10, but not the catalytically inactive NAT10-G641E mutant, significantly increased puromycin incorporation efficiency in the 8305C cell (Fig. 4H, I, S4C), as well as in KHM-5 M and FRO cells (Supplementary Fig. S4E, S4F). Moreover, the NAT10 inhibitor remodelin also suppressed global protein synthesis in ATC cells (Fig. 4J, K, S4D). Collectively, these results demonstrate that NAT10 is a critical regulator of global protein synthesis, in association with tRNA ac4C modification in ATC.

### NAT10-mediated ac^4^C modification drives ATC progression via the NAT10/tRNA/ac^4^C-c-Myc positive feedback loop

**Fig. 5:**
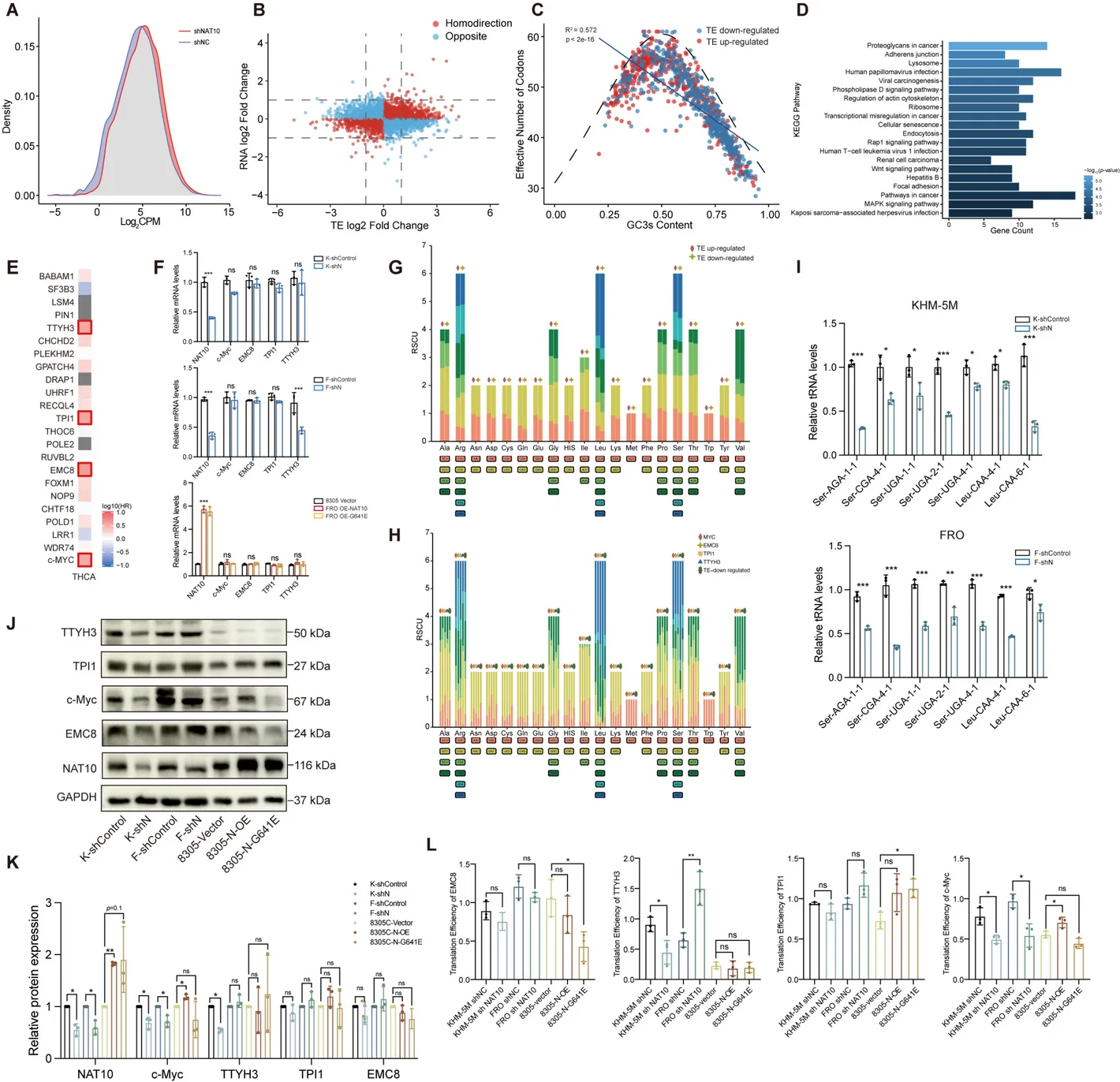
NAT10 regulates the translation of c-Myc by influencing the ac.^4^C modification of ser-tRNA and leu-tRNA. **A** Density plot of translational changes (Ribo-seq) following NAT10 knockdown. **B** Scatter plot of translation efficiency versus mRNA abundance fold-change (log₂FC) in NAT10- knockdown KHM-5 M cells. “Homodirection” and “Opposite” denote coordinated or inverse trends between mRNA and translation levels. **C** ENC-plot analysis of top 500 TE-changed genes. The black curve was the expected ENC-values versus GC3s. **D** Pathway enrichment analysis of genes with significantly downregulated TE. **E** Survival association analysis of key downregulated genes in thyroid cancer, with red boxes indicating genes significantly correlated with ATC prognosis. **F** qRT–PCR validation of representative TE-downregulated genes. **G** RSCU values of top 500 TE-downregulated and TE-upregulated genes. **H** Comparison of RSCU values of four representative TE-downregulated genes. **I** Quantitative analysis of tRNA expression in ATC cells from shControl and shN groups by stem-loop qRT–PCR. **J**–**K** Western blot analysis and band intensity quantification of TE-downregulated proteins. **L** Translational efficiency quantification (western blot/qRT–PCR ratio) of representative genes. shN: shNAT10, Data: mean ± SD; *n* = 3, *p < 0.05, **p < 0.01, and ***p < 0.001

To deeply explore the translational regulatory mechanism of NAT10-mediated tRNA ac^4^C modification in ATC progression, we conducted mRNA-seq and Ribo-seq in NAT10 knockdown cells and their control cells. The Ribo-seq results indicated that NAT10 knockdown significantly reduced the overall gene translation level in ATC cells (Fig. 5A). The translation efficiency (TE) analysis identified 1062 genes with significantly decreased translation efficiency (FDR < 0.05, *p* < 0.05; Fig. 5B). Subsequently, we performed a codon usage feature analysis on the CDS of genes with changed TE. The results showed that the ENC of both TE-upregulated and TE-downregulated genes ranged from 30 to 60, and most data points were located below the theoretical neutral curve (Fig. 5C, S5A), suggesting that their codon usage preferences might be driven by natural selection or translational optimization.

Functional pathway enrichment analysis of TE-downregulated genes revealed that they were mainly enriched in ribosome biogenesis, transcriptional dysregulation in cancer, and cancer-related pathways (Fig. 5D), further linking the function of NAT10 to key biological processes promoting tumorigenesis. Correlation analysis initially screened out 23 TE-downregulated genes significantly associated with NAT10 expression. Subsequent survival analysis further confirmed that only c-Myc, TTYH3, TPI1, and EMC8 were significantly associated with the prognosis of patients with thyroid cancer (Fig. 5E). Crucially, qRT–PCR experiments verified that the mRNA levels of these target genes did not change significantly after NAT10 knockdown (*p* > 0.05; Fig. 5F), suggesting that the regulatory mechanism occurred at the posttranscriptional level. Synonymous codon usage analysis revealed significant differences in the RSCU values of serine (Ser) and leucine (Leu) codons between TE-upregulated and TE-downregulated genes (Fig. 5G). Among them, c-Myc, TTYH3, TPI1, and EMC8 showed significantly increased RSCU values for specific codons such as AGC (Ser), CUG (Leu), GCC (Ala), GCC (Ala), AGG (Arg), CAG (Gln), and AUC (Ile) (Fig. 5H). Furthermore, the preferred codons of C‑MYC were GCC, AGG, AAC, UGC, CAG, GGC, AUC, UUG, CUG, CCC, AGC, UCC, UCG, ACC, and GUC (Supplementary Fig. S5B). Given that the c-Myc protein is rich in leucine/serine domains, and its mRNA level did not change owing to TE reduction, we further evaluated the availability of the corresponding tRNAs. The results indicated that NAT10 inhibition significantly reduced the abundance of serine and leucine tRNA (Fig. 5I), thereby affecting the TE of c-Myc. Consistent with this mechanism, only c-Myc protein expression was significantly downregulated in KHM-5 M and FRO cells after NAT10 knockdown. In contrast, overexpression of wild-typr NAT10, but not NAT10-G641E, significantly upregulated c-Myc expression **(**Fig. 5J, K). TE analysis also confirmed that only the TE of c-Myc was significantly reduced after NAT10 knockdown; overexpression of wild-type NAT10 significantly upregulated c-Myc translation efficiency, while the catalytically inactive mutant had no such effect (Fig. 5L). In addition, we constructed a c-Myc rescue model (Supplementary Fig. S5C, S5D). Rescue experiments demonstrated that c-Myc overexpression could restore the decreased proliferation (Supplementary Fig. S5E), colony formation (Supplementary Fig. S5F, S5G), and migration abilities (Supplementary Fig. S5H, S5I) of KHM-5 M cells caused by NAT10 knockdown. To verify that the reduction in c-Myc protein levels induced by NAT10 knockdown impairs its function as an oncogenic transcription factor, we examined the expression levels of the classic c-Myc target genes HK2, GLUT1, CDK4, and CCND2. We found that the mRNA levels of these four genes were significantly decreased, accompanied by markedly reduced protein expression (Supplementary Fig. S5J–5L). These results indicate that the alteration of c-Myc protein levels caused by NAT10 knockdown also affects its transcriptional activity, thereby leading to a series of changes in cell proliferation and metabolism. In summary, this study revealed that NAT10 promotes ATC development, at least in part, by enhancing the translation efficiency of the oncogene c-Myc, while c-Myc reciprocally activates NAT10 transcription, forming a positive feedback regulatory loop that jointly drives tumor progression.

### NAT10 regulates c-Myc stability through modulation of the deubiquitinase OTUB1

**Fig. 6:**
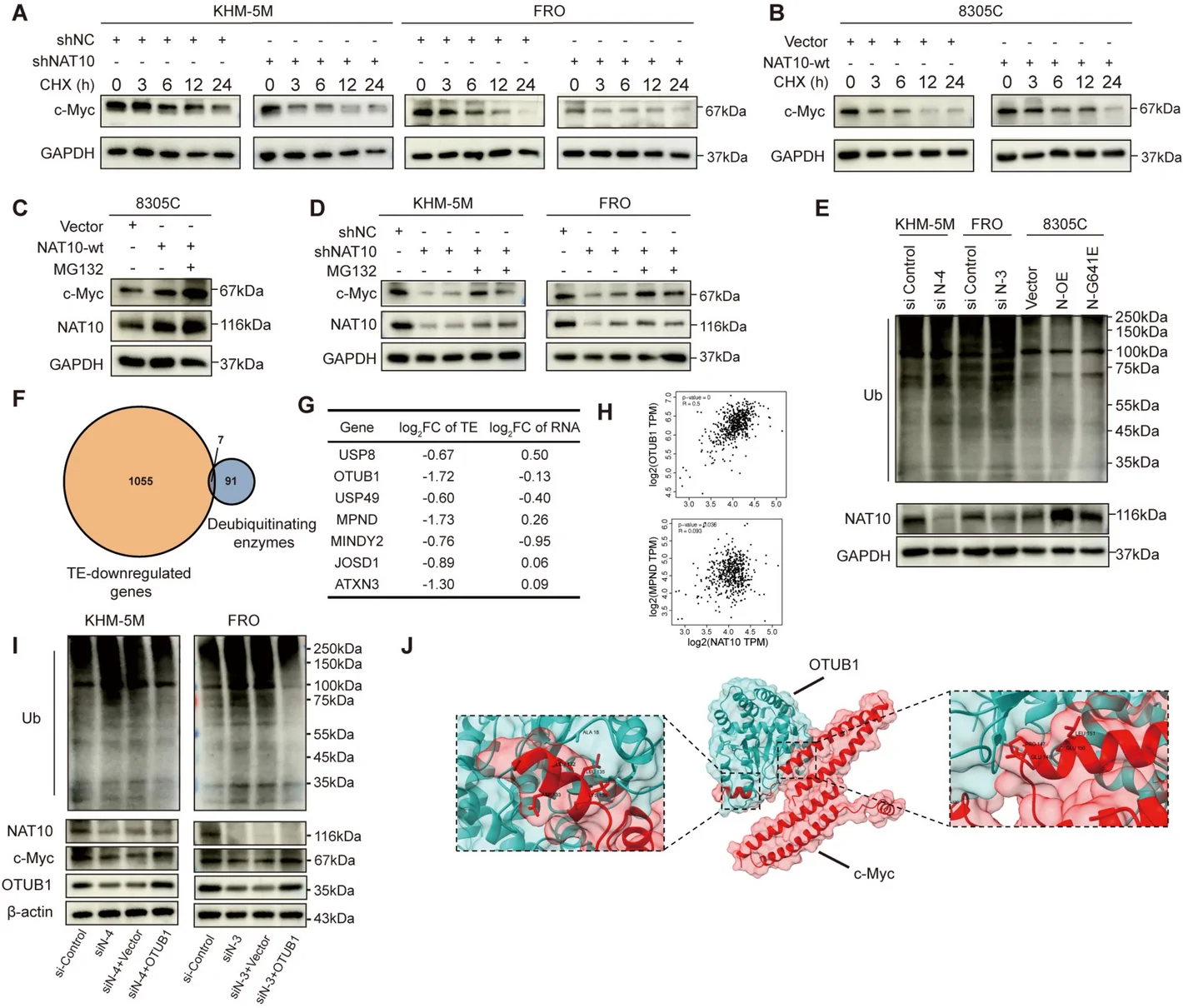
NAT10 regulates c-Myc stability through modulation of the deubiquitinase OTUB1. **A** CHX chase assay demonstrates significantly shortened c-Myc half-life following NAT10 knockdown in ATC cells. **B** CHX chase assay reveals prolonged c-Myc half-life upon NAT10 overexpression in ATC cells. **C**–**D** Restoration of c-Myc protein levels after MG132 treatment indicates ubiquitin–proteasome system-mediated degradation. **E** NAT10 modulates global ubiquitination levels in ATC cells. **F** Screening of potential functional deubiquitinating enzymes. **G** Changes in translation efficiency and RNA levels (log_2_FC) of potential functional deubiquitinating enzymes following NAT10 knockdown. **H** Correlation analysis of deubiquitinating enzymes OTUB1 and MPND with NAT10 at the RNA level. **I** NAT10 influences ubiquitination status specifically through OTUB1. **H** Schematic representation of the molecular docking between c-Myc and OTUB1.Data: mean ± SD; *n* = 3, *p < 0.05, **p < 0.01, and ***p < 0.001

Cancer progression depends not only on de novo protein synthesis but also critically on the UPS, which plays a central role in orchestrating targeted protein degradation to dynamically remodel the proteomic landscape. We therefore investigated whether NAT10 regulates c-Myc stability through protein degradation pathways. CHX chase assays revealed that NAT10 knockdown significantly shortened c-Myc protein half-life in ATC cells (Fig. 6A, S6A), while wild-type NAT10 overexpression extended it (Fig. 6B, S6B), indicating that NAT10 stabilizes c-Myc. To determine if this regulation occurs via the UPS, we treated ATC cells with the proteasome inhibitor MG132. MG132 treatment reversed the reduction in c-Myc levels caused by NAT10 knockdown (Fig. 6C-D, S6C, S6D), confirming UPS-mediated c-Myc degradation. Furthermore, NAT10 knockdown leads to elevated ubiquitination levels in KHM-5 M and FRO cells. In contrast, overexpression of wild-type NAT10, but not NAT10-G641E, significantly suppressed global ubiquitination in 8305C cells (Fig. 6E, S6E). By integrating Ribo-seq data and using Venn diagrams to analyze the intersection of TE-downregulated genes and deubiquitinating enzyme (DUB) genes, a total of seven overlapping genes were identified (Fig. 6F). Further, on the basis of the screening criteria (TE |log_2_FC|> 1.5), two candidate genes (OTUB1 and MPND) were ultimately obtained (Fig. 6G). Correlation analysis revealed significantly stronger association between OTUB1 and NAT10 expression (*p* < 0.001; R = 0.5) **(**Fig. 6H), Therefore, OTUB1 was selected as the key UPS-related candidate for functional validation. Functional verification experiments demonstrated that in KHM-5 M and FRO cells with NAT10 knockdown, the expression level of OTUB1 was significantly downregulated, accompanied by a marked reduction in c-Myc protein expression. Exogenous overexpression of OTUB1 could effectively restore the c-Myc protein level (Fig. 6I, Supplementary Fig. S6F). Ubiquitination analysis results indicated that overexpression of OTUB1 could specifically reverse the elevated c-Myc ubiquitination level caused by NAT10 knockdown, confirming the existence of the NAT10–OTUB1–c-Myc regulatory axis. Molecular docking reveals a direct interaction between OTUB1 and c-MYC, where the interference face acts as a small hook of c-MYC, encapsulated by a chamber-like structure of OTUB1. The interference face primarily consists of residues 1–15 of the c-MYC D-chain, corresponding to amino acids 129–133 in the entire PDB file (Fig. 6J). Collectively, these data uncover a previously unrecognized posttranslational regulatory arm of the NAT10/c-Myc axis, wherein NAT10 stabilizes the c-Myc oncoprotein by orchestrating OTUB1-mediated deubiquitination, thereby expanding the mechanistic scope of the NAT10-driven oncogenic circuit beyond transcriptional–translational control.

### NAT10 inhibition and doxorubicin treatment synergistically inhibit ATC progression

**Fig. 7:**
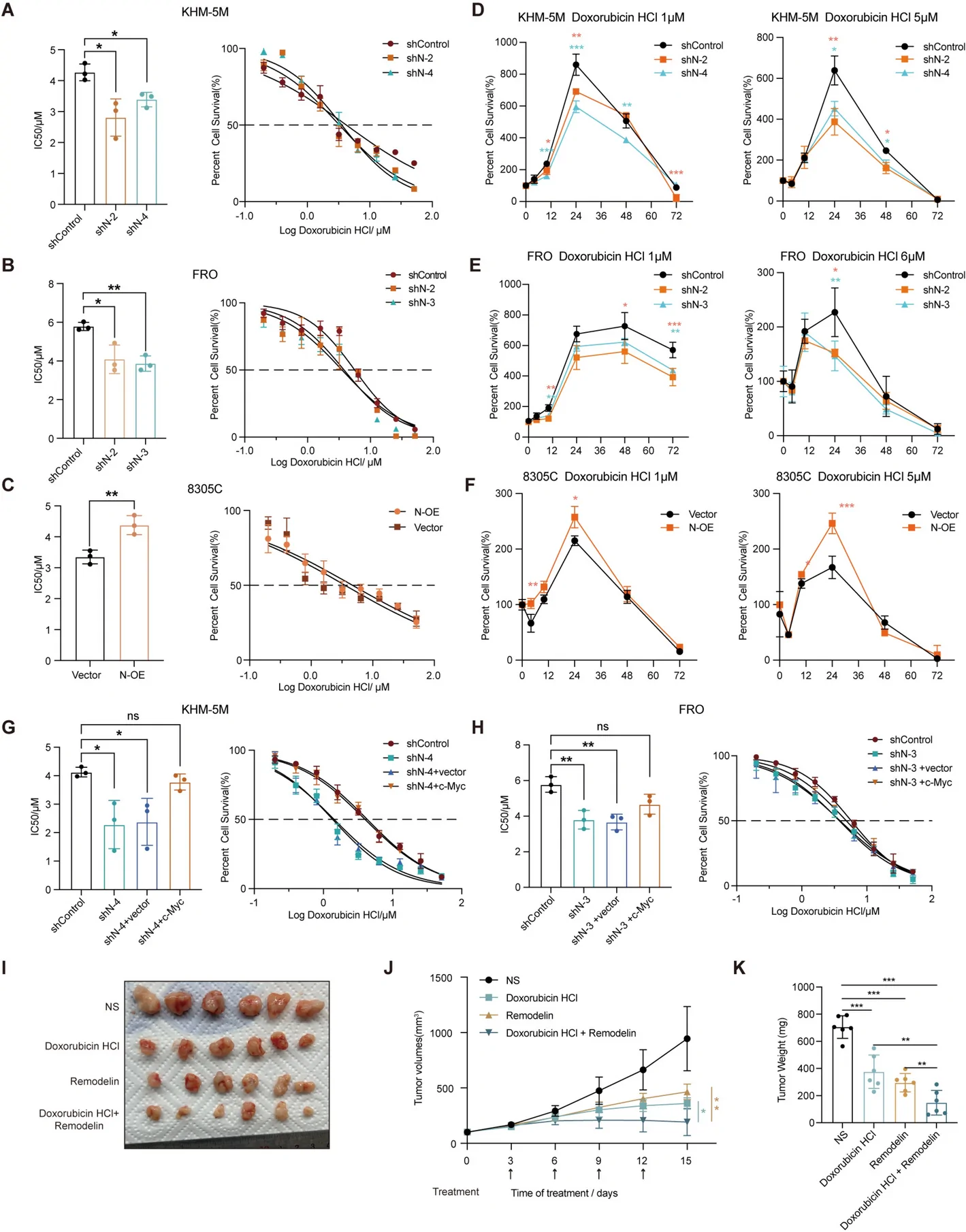
NAT10 inhibition and doxorubicin treatment synergistically inhibit ATC progression. **A**–**C** Cell growth inhibition assays and statistical analyses evaluating the effect of NAT10 knockdown on doxorubicin HCl sensitivity in KHM-5 M **A** and FRO **B** cells, and NAT10 overexpression in 8305C cells **C**. IC_50_, half-maximal inhibitory concentration. Tukey HSD test. **D**–**F** Time-dependent response curves of vector, shN, and N-OE cells to high/low concentrations of doxorubicin HCl. Pairwise comparisons of estimated marginal means. **G**–**H** Overexpression of c-Myc can rescue the reduction in IC_50_ induced by NAT10 knockdown in ATC cells. **I** Overview of tumors in xenograft models treated with remodelin or doxorubicin HCl in ATC cells. **J**–**K** Tumor volume growth curves and weight of xenograft models treated with remodelin or doxorubicin HCl in ATC cells. Data: mean ± SD; *n* = 3, *p < 0.05, **p < 0.01, and ***p < 0.001

To translate the oncogenic dependency on NAT10 into therapeutic opportunity, we assessed the combinatorial efficacy of NAT10-targeted agents with standard-of-care doxorubicin in ATC models. NAT10 knockdown substantially lowered doxorubicin IC_50_ in KHM-5 M and FRO cells (Fig. 7A, B), whereas overexpression elevated IC_50_ in 8305C cells (Fig. 7C). Furthermore, time-course analysis demonstrated that NAT10 knockdown shortened the plateau phase of cell growth and potentiated the therapeutic efficacy of doxorubicin (Fig. 7D, E). Specifically, under low-dose doxorubicin treatment, NAT10 knockdown significantly sensitized ATC cells to the drug, reducing their resistance (Fig. 7D, E). Conversely, NAT10 overexpression in 8305C cells markedly enhanced their tolerance to doxorubicin (Fig. 7F). Subsequent CCK-8 assay results showed that the reduction in the IC_50_ value of doxorubicin caused by NAT10 knockdown in KHM-5 M cells could be reversed by c-Myc overexpression (Fig. 7G, H). To further evaluate the combined antitumor effect of remodelin and doxorubicin HCl, we assessed cell viability in KHM-5 M and FRO cells treated with increasing concentrations of remodelin in combination with doxorubicin HCl. As shown in Supplementary Fig. S7A, the combination treatment resulted in a dose-dependent increase in the inhibition rate of cell viability. The interaction between the two agents was analyzed using the combination index (CI). Notably, CI values were consistently less than 1 across a range of inhibitory effects in both cell lines (Supplementary Fig. S7B), indicating that remodelin and doxorubicin HCl act synergistically to inhibit the viability of ATC cells in vitro. These data unequivocally demonstrate that the NAT10–c-Myc regulatory loop mediates doxorubicin resistance in ATC cells.

In vivo experiments demonstrated that the combination of remodelin and doxorubicin significantly inhibited tumor growth, showing statistically significant differences compared with single-drug treatments or the blank control group (Fig. 7I). Tumor growth kinetics were markedly slowed (Fig. 7J), and the final tumor weight was also significantly reduced (Fig. 7K). To evaluate the in vivo safety of the treatments, we monitored body weight in mice from different groups and performed HE staining of vital organs including the liver, kidney and spleen. No significant differences in body weight were observed among groups (Supplementary Fig. S7C). The hepatic lobules and hepatic cords were structurally intact, and hepatocytes exhibited normal morphology. The spleens were normal in size without obvious inflammatory infiltration. The kidneys showed no signs of inflammation, glomerular fibrosis, or tubular dilation (Supplementary Fig. S7D). Furthermore, histological analysis of xenograft tumors by H&E staining revealed a significant increase in tissue necrosis foci within the combination group compared with single-drug groups (Supplementary Fig. S7E). IHC analysis further showed that Ki67 staining intensity was most significantly decreased in the combination group, indicating effective suppression of ATC cell proliferation. Notably, increased caspase-3 staining intensity was observed in all drug-treated groups, consistent with chemotherapy-induced apoptosis (Supplementary Fig. S7E). These results suggest that remodelin and doxorubicin treatment synergistically inhibit ATC progression.

## Discussion

Our study reveals significant NAT10 overexpression in ATC, correlating with poor patient prognosis. We demonstrate that NAT10, leveraging its acetyltransferase activity, promotes ATC cell proliferation and metastasis, with evidence supporting a role for tRNA ac^4^C modification in this process. This epitranscriptomic modification functions as a key mediator of translational reprogramming, specifically enhancing the expression of oncogenes such as c-Myc. Crucially, we uncover a positive feedback loop between NAT10 and c-Myc: c-Myc transcriptionally activates NAT10, while NAT10 posttranslationally stabilizes the c-Myc protein via regulation of the deubiquitinase OTUB1. This dual-layered regulatory mechanism significantly amplifies c-Myc’s oncogenic potential and reveals a novel posttranslational pathway underpinning the aggressive ATC phenotype. Consistent findings from both in vitro and in vivo models indicate that targeting NAT10 represents a promising therapeutic strategy for ATC, demonstrating synergistic antitumor efficacy when combined with doxorubicin. Thus, our work not only elucidates key molecular mechanisms driving ATC progression but also nominates NAT10 as a promising therapeutic target for this aggressive malignancy.

NAT10, a conserved RNA acetyltransferase, catalyzes ac^4^C modification on tRNAs and mRNAs, regulating RNA stability and translational efficiency [16]. In ATC, we demonstrate significant NAT10 overexpression correlating with poor patient survival, aligning with its emerging oncogenic activity in diverse tumors [23]. Although large-scale data associate high NAT10 expression with poor prognosis across multiple cancer types, and functional studies in cervical [24], bladder [18] and gastric malignancies [25] have attributed this to NAT10-mediated stabilization of oncogenic mRNAs (FOXP1, AHNAK, CXCL2 and KLF5), the contribution of tRNA ac^4^C modifications to cancer pathogenesis remains largely unexplored.

Crucially, our study unveils a tRNA-centric mechanism for NAT10 in ATC: NAT10 inhibition markedly reduced ac^4^C modifications specifically in tRNA-Leu (CAA, CAG) and tRNA-Ser (AGA, GCU, UGA, CGA) isoforms, aligning with established tRNA substrate preferences [26, 27]. These modifications confer structural rigidity and flexibility essential for translational fidelity [28], explaining the observed decline in tRNA abundance and global translation efficiency upon NAT10 depletion. We further identified a self-reinforcing c-Myc–NAT10 axis. c-Myc, a master oncogenic driver, transcriptionally upregulates NAT10. Conversely, NAT10 optimizes c-Myc translation through tRNA ac^4^C modification. This dependency arises from c-Myc’s leucine-rich HLH-Zip dimerization domain and Ser-62 phosphorylation site [29, 30]. Reduced Leu/Ser-tRNA availability following NAT10 knockdown selectively impairs decoding of serine/leucine-rich codons in c-MYC mRNA, diminishing its translation efficiency without altering mRNA levels. Thus, NAT10-associated tRNA ac4C modifications may act as a codon-specific translational regulatory mechanism for oncoproteins such as c-Myc.

Beyond translational control, we uncovered a novel layer of c-Myc stabilization. NAT10 depletion shortened c-Myc protein half-life, while overexpression extended it-effects reversed by proteasome inhibition (MG132). Mechanistically, NAT10 sustains OTUB1 levels, enabling OTUB1 to deubiquitinate and stabilize c-Myc. OTUB1 overexpression rescued c-Myc ubiquitination and degradation induced by NAT10 knockdown, establishing the NAT10–OTUB1–c-Myc axis. This aligns with OTUB1’s role in stabilizing oncoproteins [31], and specifically confirms its regulation of c-Myc in ATC. This dual regulation-enhanced synthesis and suppressed degradation-creates a “double-lock” mechanism ensuring sustained oncogenic c-Myc levels. Therapeutically, remodelin—a selective NAT10 inhibitor that binds its acetyl-CoA pocket—holds therapeutic promise [32]. While remodelin exhibits efficacy across multiple disease and synergizes with cisplatin in bladder cancer [32], our study provides the first evidence of its potential in ATC. Remodelin phenocopied genetic NAT10 knockdown, effectively inhibiting ATC cell proliferation, colony formation, global ac^4^C modification, and protein synthesis. Critically, it demonstrated synergistic efficacy with doxorubicin: in vitro, it sensitized ATC cells by lowering the IC_50_ of doxorubicin; in vivo, combination therapy significantly suppressed tumor growth and enhanced necrosis. This chemosensitization stems from remodelin’s disruption of the NAT10–c-Myc–OTUB1 axis, by inhibiting oncoprotein translation and promoting the degradation of stabilized factors such as c-Myc. Although optimization of remodelin’s pharmacokinetic properties is warranted, our findings validate NAT10 as a promising druggable target and support the development of combined epitranscriptomic inhibition and conventional chemotherapy strategies for ATC.

We acknowledge that NAT10 is a multisubstrate enzyme capable of acetylating tRNAs, rRNAs, mRNAs, and proteins. Therefore, although our multi-omics data support a strong association between NAT10 activity, tRNA ac^4^C modification, and translational regulation of c-Myc, we cannot fully exclude contributions from other NAT10 substrates. Importantly, while our findings are consistent with a tRNA-mediated mechanism, definitive dissection of substrate specificity will require additional approaches, such as THUMPD1-based models. These will be important directions for future studies. Nevertheless, the convergence of multiple independent lines of evidence—including catalytic activity dependence, global reduction of tRNA ac^4^C levels, codon usage bias, and translation efficiency changes without mRNA alteration—collectively supports a major role for tRNA-associated mechanisms in NAT10-driven ATC progression.

## Conclusions

Collectively, our study reveals the NAT10/c-Myc positive feedback loop as a pivotal driver of ATC progression. NAT10—transcriptionally activated by c-Myc—promotes tumorigenesis through tRNA ac^4^C modification to enhance oncogenic translation and via OTUB1-mediated deubiquitination to stabilize c-Myc protein. Pharmacological inhibition of NAT10 with remodelin suppresses ATC growth and metastasis, while its combination with doxorubicin exhibits potent synergistic efficacy in preclinical models. Targeting this axis not only overcomes current treatment resistance but also paves the way for novel therapeutic avenues to improve survival in ATC patients.

## Supplementary Information


Supplementary Material 1.
Supplementary Material 2.
Supplementary Material 3.
Supplementary Material 4.
Supplementary Material 5.
Supplementary Material 6.
Supplementary Material 7.
Supplementary Material 8.
Supplementary Material 9.
Supplementary Material 10.


## Data Availability

The datasets generated during and/or analyzed during the current study are available from the corresponding author on reasonable request.

## Abbreviations

ATC: Anaplastic thyroid carcinoma
NAT10: *N*-acetyltransferase 10
tRNA: Transfer RNA
ac^4^C: *N*^4^-acetylcytidine
UPS: Ubiquitin–proteasome system
IHC: Immunohistochemistry
IF: Immunofluorescence
H&E: Hematoxylin–eosin
WB: Western blotting
RT–qPCR: Quantitative reverse-transcription polymerase chain reaction
ATCC: American Type Culture Collection
STR: Short tandem repeat
IC₅₀: Half-maximal inhibitory concentration
ChIP: Chromatin immunoprecipitation
BS: Binding sites
RPFs: Ribosome-protected fragments
CDS: Coding sequences
TE: Translation efficiency
CHX: Cycloheximide
MRM: Multi-reaction monitoring
RSCU: Relative synonymous codon usage
ENC: Effective number of codons
GC3s: GC content at the third position of synonymous codons
DUB: Deubiquitinating enzyme

## References

[1] Pozdeyev N, Rose MM, Bowles DW, Schweppe RE. Molecular therapeutics for anaplastic thyroid cancer. Semin Cancer Biol. 2020;61:23–9. 31991166 10.1016/j.semcancer.2020.01.005 PMC7117889

[2] Califano I, Smulever A, Jerkovich F, Pitoia F. Advances in the management of anaplastic thyroid carcinoma: transforming a life-threatening condition into a potentially treatable disease. Rev Endocr Metab Disord. 2024;25(1):123–47. 37648897 10.1007/s11154-023-09833-1

[3] Wang W, Ouyang Q, Meng C, Jing L, Li X. Treatment optimization and prognostic considerations for primary squamous cell carcinoma of the thyroid. Gland Surg. 2019;8(6):683–90. 32042676 10.21037/gs.2019.11.07 PMC6989903

[4] Yamazaki H, Sugino K, Katoh R, Masudo K, Matsuzu K, Kitagawa W, et al. Impact of local control on clinical course in stage IVC anaplastic thyroid carcinoma. World J Surg. 2022;46(12):3034–42. 36127501 10.1007/s00268-022-06739-y

[5] Lim AM, Solomon BJ. Immunotherapy for anaplastic thyroid carcinoma. J Clin Oncol. 2020;38(23):2603–4. 32552275 10.1200/JCO.20.01437

[6] Wu Z, Zhou R, Li B, Cao M, Wang W, Li X. Methylation modifications in tRNA and associated disorders: current research and potential therapeutic targets. Cell Prolif. 2024;57(9):e13692. 38943267 10.1111/cpr.13692 PMC11503269

[7] Tao EW, Wang Y, Tan J, Chen Y, Sun TY, Hao Y, et al. TRMT6-mediated tRNA m(1)A modification acts as a translational checkpoint of histone synthesis and facilitates colorectal cancer progression. Nat Cancer. 2025. 10.1038/s43018-025-00977-4. 40461825

[8] Akiyama Y, Ivanov P. tRNA-derived RNAs: biogenesis and roles in translational control. Wiley Interdiscip Rev RNA. 2023;14(6):e1805. 37406666 10.1002/wrna.1805 PMC10766869

[9] Cui W, Zhao D, Jiang J, Tang F, Zhang C, Duan C. tRNA modifications and modifying enzymes in disease, the potential therapeutic targets. Int J Biol Sci. 2023;19(4):1146–62. 36923941 10.7150/ijbs.80233 PMC10008702

[10] Wang Y, Tao EW, Tan J, Gao QY, Chen YX, Fang JY. tRNA modifications: insights into their role in human cancers. Trends Cell Biol. 2023;33(12):1035–48. 37179136 10.1016/j.tcb.2023.04.002

[11] Wang W, Ding Y, Zhao H, Wang S, Huang J, Sun L. NSUN2-tRNA(Val-CAC)-axis-regulated codon-biased translation drives triple-negative breast cancer glycolysis and progression. Cell Mol Biol Lett. 2025;30(1):100. 40855521 10.1186/s11658-025-00781-z PMC12376479

[12] Li P, Wang W, Zhou R, Ding Y, Li X. The m(5) C methyltransferase NSUN2 promotes codon-dependent oncogenic translation by stabilising tRNA in anaplastic thyroid cancer. Clin Transl Med. 2023;13(11):e1466. 37983928 10.1002/ctm2.1466 PMC10659772

[13] Masuda I, Yamaki Y, Detroja R, Tagore S, Moore H, Maharjan S, et al. tRNA methylation resolves codon usage bias at the limit of cell viability. Cell Rep. 2022;41(4):111539. 36288695 10.1016/j.celrep.2022.111539 PMC9643105

[14] Liao L, He Y, Li SJ, Yu XM, Liu ZC, Liang YY, et al. Lysine 2-hydroxyisobutyrylation of NAT10 promotes cancer metastasis in an ac4C-dependent manner. Cell Res. 2023;33(5):355–71. 36882514 10.1038/s41422-023-00793-4 PMC10156899

[15] Gu Z, Zou L, Pan X, Yu Y, Liu Y, Zhang Z, et al. The role and mechanism of NAT10-mediated ac4C modification in tumor development and progression. MedComm. 2024;5(12):e70026. 39640362 10.1002/mco2.70026 PMC11617596

[16] Zhang Y, Lei Y, Dong Y, Chen S, Sun S, Zhou F, et al. Emerging roles of RNA ac4C modification and NAT10 in mammalian development and human diseases. Pharmacol Ther. 2024;253:108576. 38065232 10.1016/j.pharmthera.2023.108576

[17] Zheng X, Wang Q, Zhou Y, Zhang D, Geng Y, Hu W, et al. N-acetyltransferase 10 promotes colon cancer progression by inhibiting ferroptosis through N4-acetylation and stabilization of ferroptosis suppressor protein 1 (FSP1) mRNA. Cancer Commun (Lond). 2022;42(12):1347–66. 36209353 10.1002/cac2.12363 PMC9759759

[18] Xie R, Cheng L, Huang M, Huang L, Chen Z, Zhang Q, et al. NAT10 drives cisplatin chemoresistance by enhancing ac4C-associated DNA repair in bladder cancer. Cancer Res. 2023;83(10):1666–83. 36939377 10.1158/0008-5472.CAN-22-2233

[19] Arora S, Yang JM, Hait WN. Identification of the ubiquitin-proteasome pathway in the regulation of the stability of eukaryotic elongation factor-2 kinase. Cancer Res. 2005;65(9):3806–10. 15867377 10.1158/0008-5472.CAN-04-4036

[20] Wang X, Ling R, Peng Y, Qiu W, Chen D. RNPS1 stabilizes NAT10 protein to facilitate translation in cancer via tRNA ac(4)C modification. Int J Oral Sci. 2024;16(1):6. 38246918 10.1038/s41368-023-00276-7 PMC10800354

[21] Sun Z, Li L, Wu Y, Zhang L, Zang G, Qian Y, et al. Acetylation-ubiquitination crosstalk of DJ-1 mediates microcalcification formation in diabetic plaques via collagen-matrix vesicles interaction. Cardiovasc Res. 2025;121(2):296–310. 39786474 10.1093/cvr/cvae263

[22] Liu X, Tan Y, Zhang C, Zhang Y, Zhang L, Ren P, et al. NAT10 regulates p53 activation through acetylating p53 at K120 and ubiquitinating Mdm2. EMBO Rep. 2016;17(3):349–66. 26882543 10.15252/embr.201540505 PMC4772976

[23] Yang C, Wu T, Zhang J, Liu J, Zhao K, Sun W, et al. Prognostic and immunological role of mRNA ac4C regulator NAT10 in Pan-Cancer: new territory for cancer research? Front Oncol. 2021;11:630417. 34094911 10.3389/fonc.2021.630417 PMC8170476

[24] Chen X, Hao Y, Liu Y, Zhong S, You Y, Ao K, et al. NAT10/ac4C/FOXP1 promotes malignant progression and facilitates immunosuppression by reprogramming glycolytic metabolism in cervical cancer. Adv Sci (Weinh). 2023;10(32):e2302705. 37818745 10.1002/advs.202302705 PMC10646273

[25] Chen C, Wang Z, Lin Q, Li M, Xu L, Fu Y, et al. NAT10 promotes gastric cancer liver metastasis by modulation of M2 macrophage polarization and metastatic tumor cell hepatic adhesion. Adv Sci (Weinh). 2025;12(15):e2410263. 39985269 10.1002/advs.202410263 PMC12005778

[26] Sas-Chen A, Thomas JM, Matzov D, Taoka M, Nance KD, Nir R, et al. Dynamic RNA acetylation revealed by quantitative cross-evolutionary mapping. Nature. 2020;583(7817):638–43. 32555463 10.1038/s41586-020-2418-2 PMC8130014

[27] Wang C, Hou X, Guan Q, Zhou H, Zhou L, Liu L, et al. RNA modification in cardiovascular disease: implications for therapeutic interventions. Signal Transduct Target Ther. 2023;8(1):412. 37884527 10.1038/s41392-023-01638-7 PMC10603151

[28] Lorenz C, Lunse CE, Morl M. tRNA modifications: impact on structure and thermal adaptation. Biomolecules. 2017. 10.3390/biom7020035. 28375166 PMC5485724

[29] Hsieh AL, Walton ZE, Altman BJ, Stine ZE, Dang CV. MYC and metabolism on the path to cancer. Semin Cell Dev Biol. 2015;43:11–21. 26277543 10.1016/j.semcdb.2015.08.003 PMC4818970

[30] Amati B, Dalton S, Brooks MW, Littlewood TD, Evan GI, Land H. Transcriptional activation by the human c-Myc oncoprotein in yeast requires interaction with Max. Nature. 1992;359(6394):423–6. 1406955 10.1038/359423a0

[31] Li Y, Yang JY, Xie X, Jie Z, Zhang L, Shi J, et al. Preventing abnormal NF-κB activation and autoimmunity by Otub1-mediated p100 stabilization. Cell Res. 2019;29(6):474–85. 31086255 10.1038/s41422-019-0174-3 PMC6796864

[32] Larrieu D, Britton S, Demir M, Rodriguez R, Jackson SP. Chemical inhibition of NAT10 corrects defects of laminopathic cells. Science. 2014;344(6183):527–32. 24786082 10.1126/science.1252651 PMC4246063

