# Supplementary material for: The NAT10/c-Myc positive feedback loop orchestrates tRNA ac4C modification and OTUB1-mediated protein stabilization to drive anaplastic thyroid carcinoma progression
Source: Cell Mol Biol Lett. 2026 Jun 12;31:139. doi: 10.1186/s11658-026-00977-x (PMC13523248; doi:10.1186/s11658-026-00977-x)
Supplement: Supplementary file 1 [file 11658_2026_977_MOESM8_ESM.pdf]

# Figure 1F

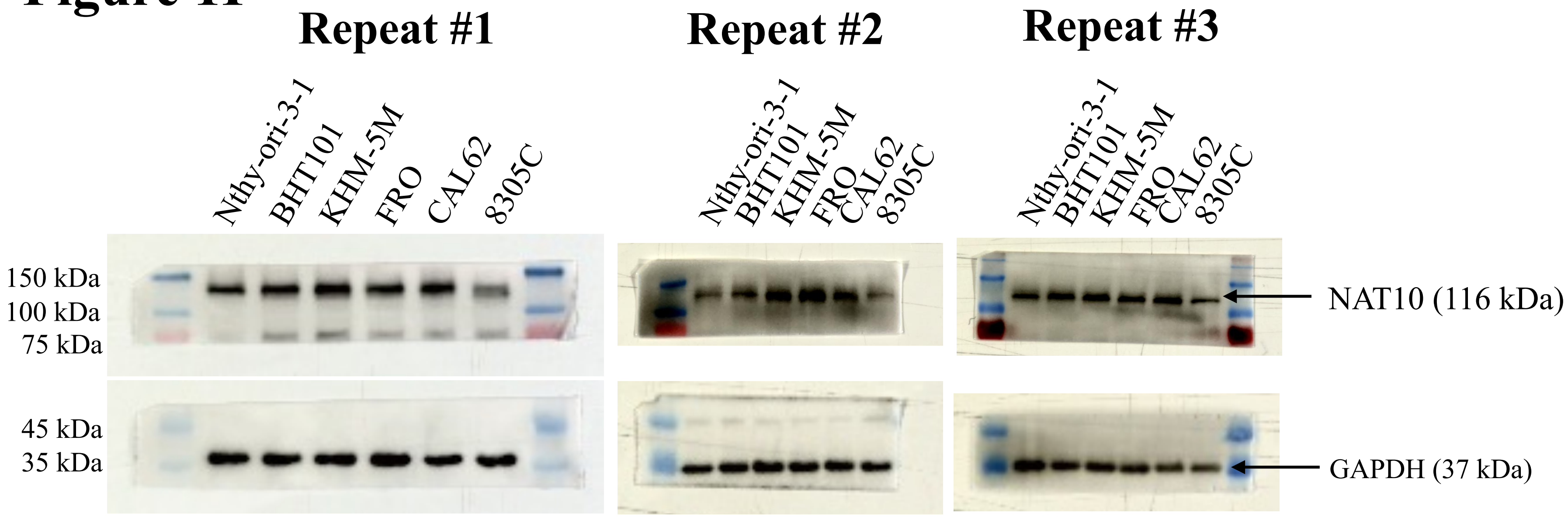


Following transfer, the membrane was cut into strips and probed separately with anti-GAPDH and anti-NAT10 antibodies.

**Western Blot Band Densitometry Measurement**

| Sample | Repeat #1 | | Repeat #2 | | Repeat #3 | |
|---|---|---|---|---|---|---|
| | NAT10 | GAPDH | NAT10 | GAPDH | NAT10 | GAPDH |
| Nthy-ori-3-1 | 19128 | 26164 | 20991 | 14121 | 15037 | 26982 |
| BHT101 | 22023 | 23440 | 22829 | 14331 | 16253 | 22454 |
| KHM-5M | 23436 | 24594 | 26048 | 14790 | 16063 | 21546 |
| FRO | 22544 | 26768 | 26957 | 14838 | 15185 | 20579 |
| CAL62 | 22712 | 20187 | 24159 | 14419 | 16091 | 18027 |
| 8305C | 14704 | 21776 | 16964 | 13107 | 8614 | 17057 |

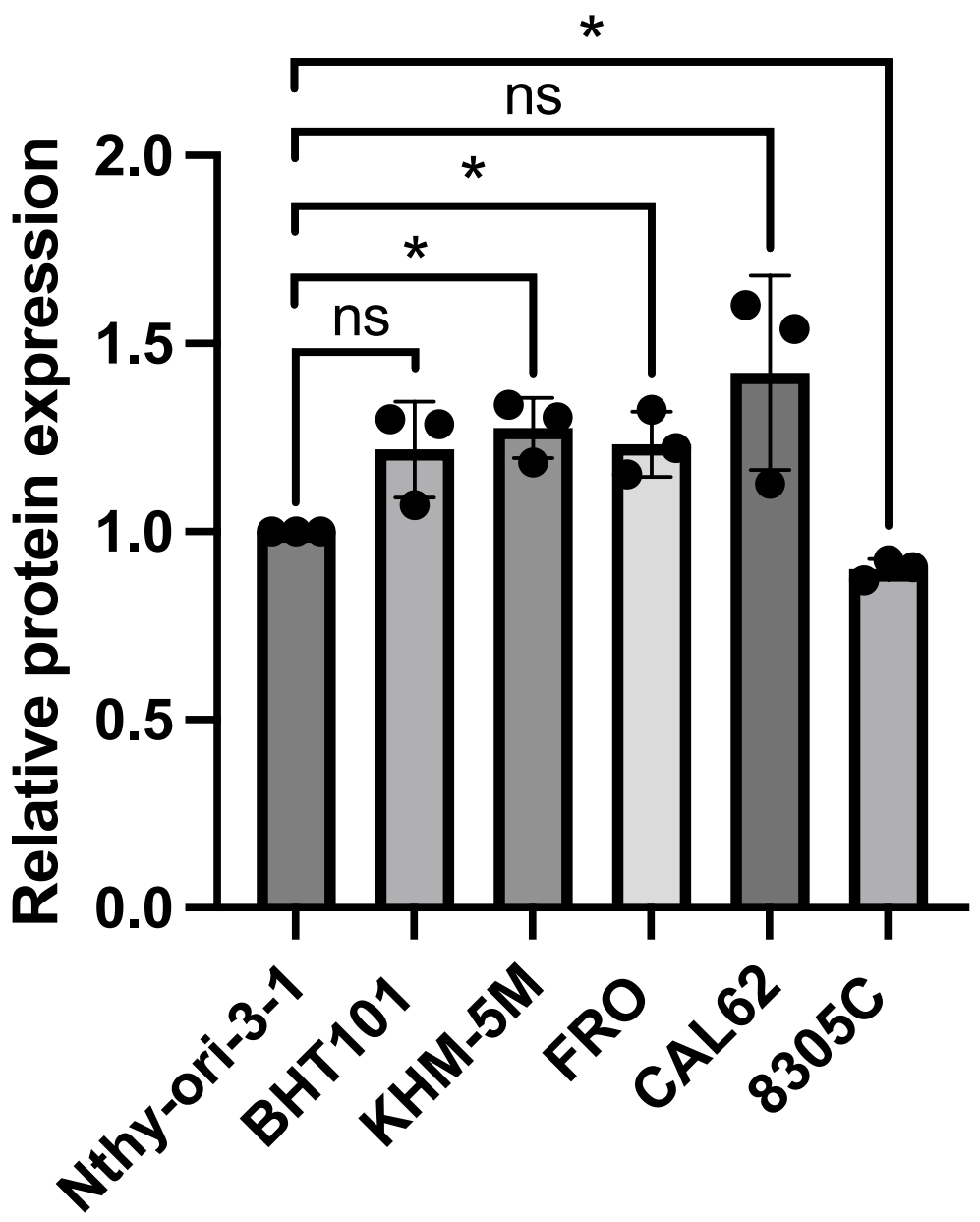

# Figure 1G

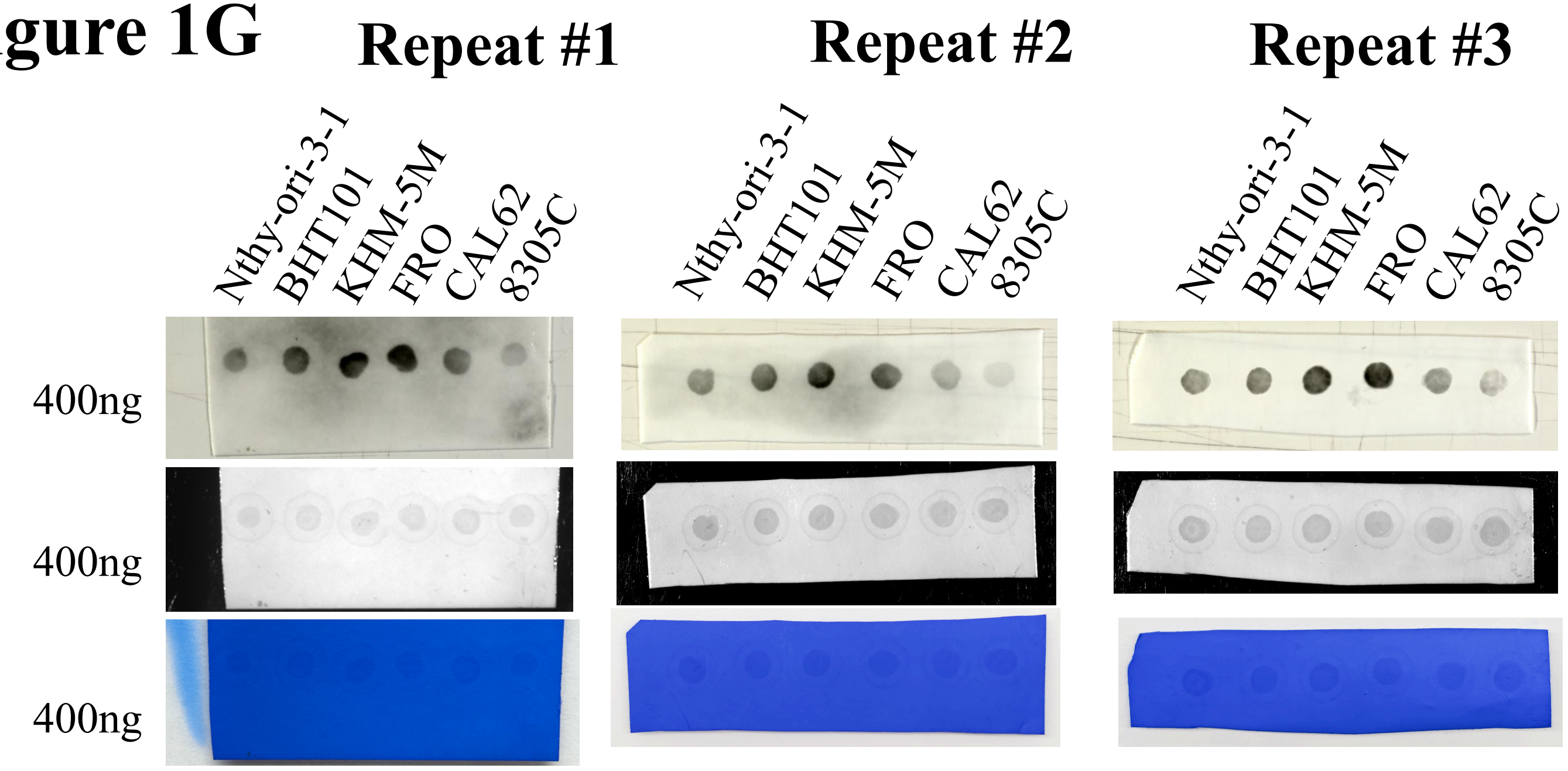


**Dot Blot Band Densitometry Measurement**

| Sample | Repeat #1 | Repeat #2 | Repeat #3 |
|---|---|---|---|
| | ac$^4$c | ac$^4$c | ac$^4$c |
| Nthy-ori-3-1 | 14078 | 21301 | 15930 |
| BHT101 | 21471 | 29865 | 16783 |
| KHM-5M | 28276 | 41195 | 30944 |
| FRO | 26692 | 32887 | 34237 |
| CAL62 | 14522 | 14851 | 14495 |
| 8305C | 7035 | 6222 | 8251 |

The entire membrane was probed with an antibody against N4-acetylcytidine.

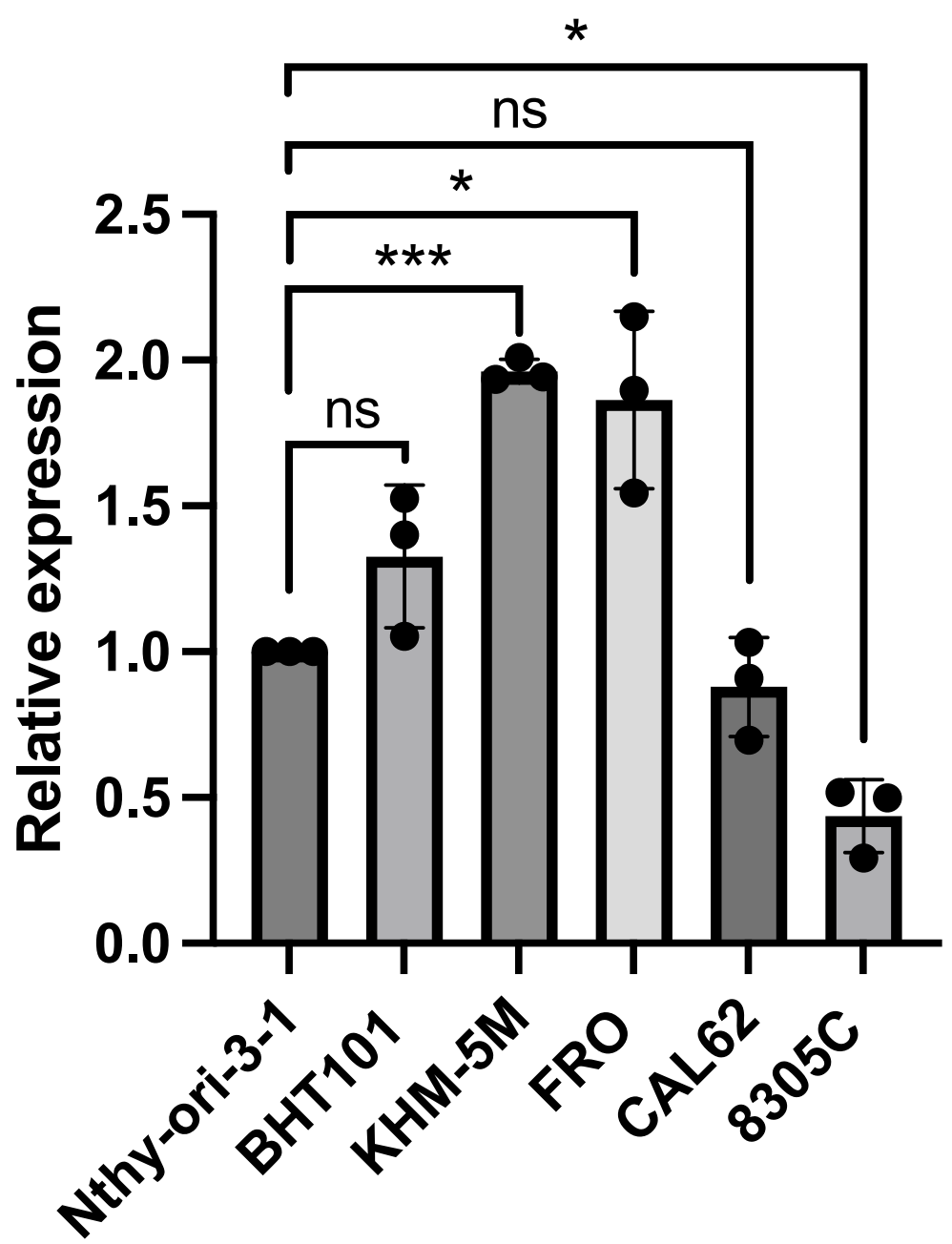

# Figure 2A - KHM-5M

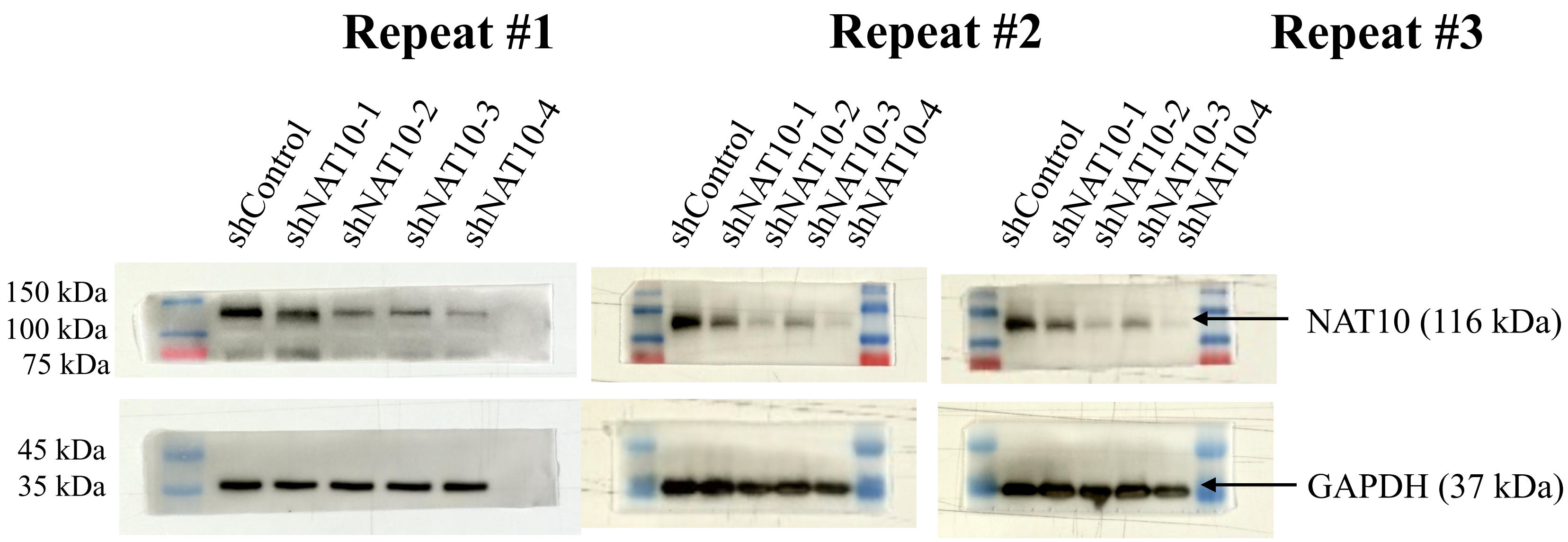


Following transfer, the membrane was cut into strips and probed separately with anti-GAPDH and anti-NAT10 antibodies.

**Western Blot Band Densitometry Measurement**

| Sample | Repeat #1 | | Repeat #2 | | Repeat #3 | |
|---|---|---|---|---|---|---|
| | NAT10 | GAPDH | NAT10 | GAPDH | NAT10 | GAPDH |
| shControl | 22599 | 20963 | 20165 | 20278 | 17489 | 23069 |
| shNAT10-1 | 19835 | 19262 | 12473 | 19841 | 11122 | 22381 |
| shNAT10-2 | 14013 | 19284 | 6477 | 16812 | 5088 | 21668 |
| shNAT10-3 | 13113 | 18795 | 8381 | 17283 | 6673 | 20618 |
| shNAT10-4 | 9716 | 19121 | 4781 | 15426 | 2197 | 16068 |

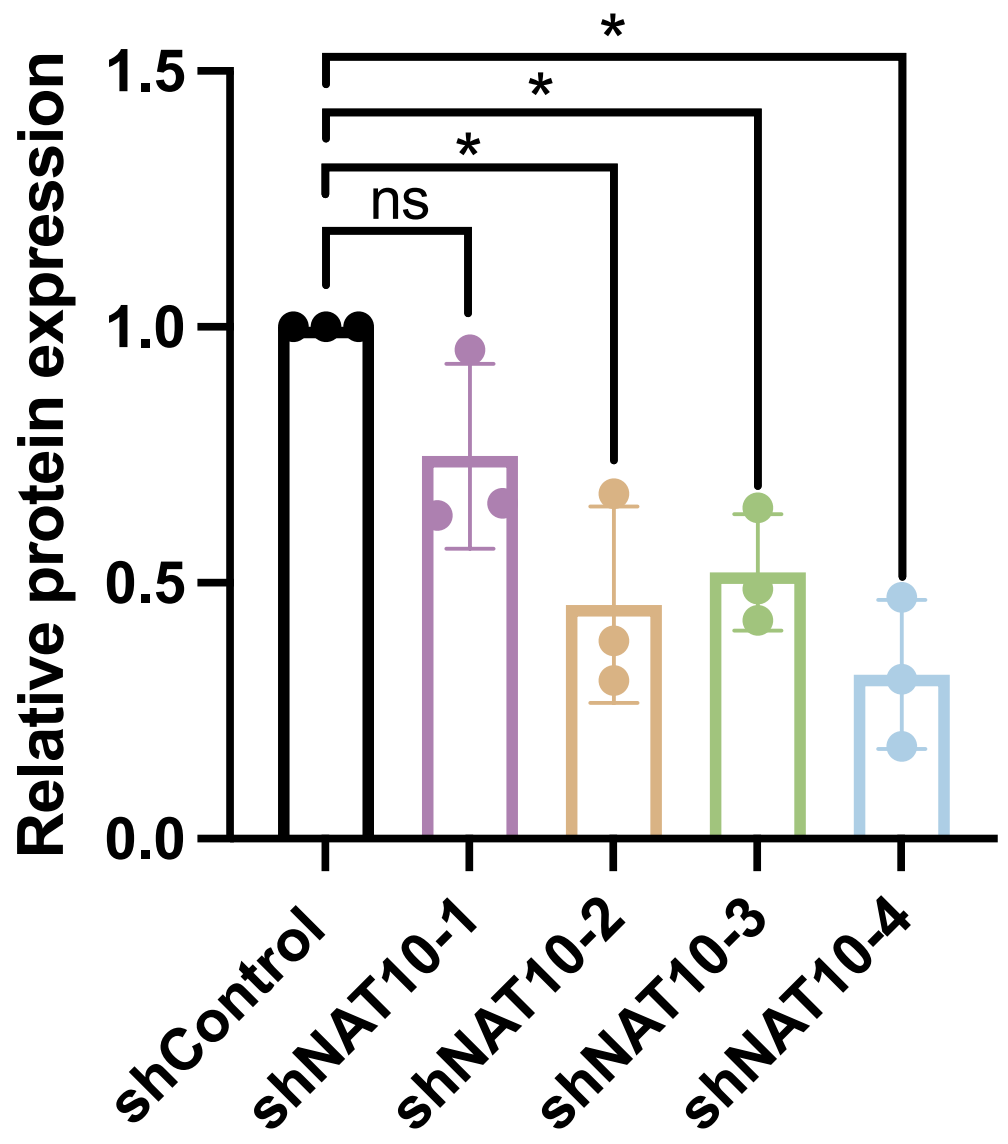

# Figure 2A - FRO

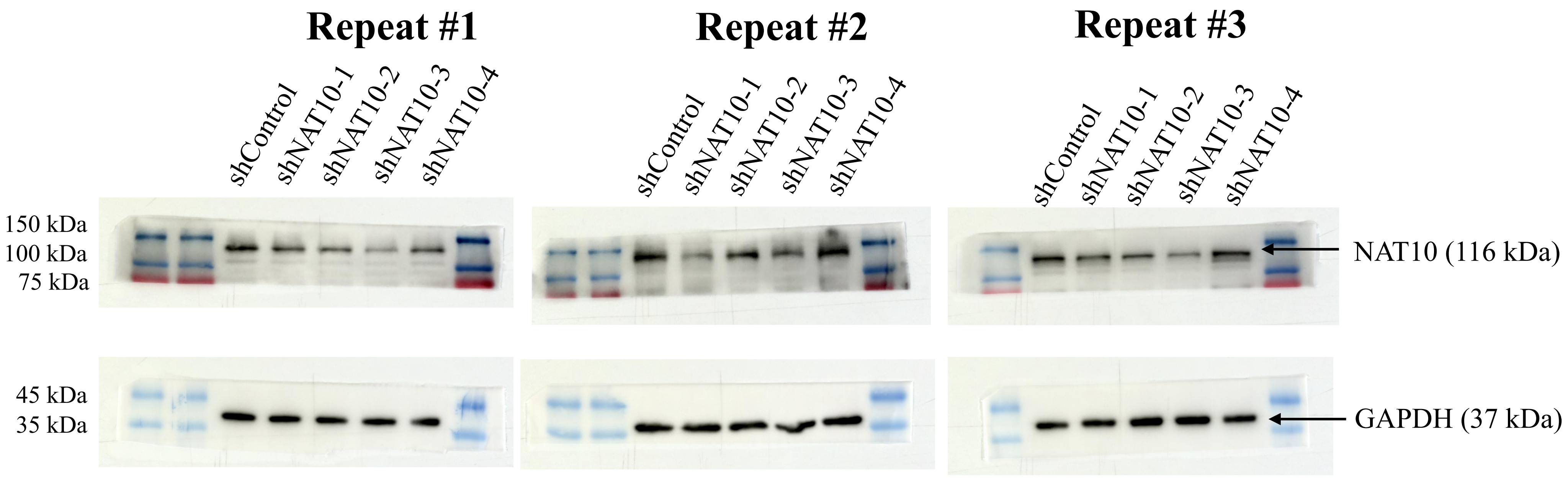


Following transfer, the membrane was cut into strips and probed separately with anti-GAPDH and anti-NAT10 antibodies.

**Western Blot Band Densitometry Measurement**

| Sample | Repeat #1 | | Repeat #2 | | Repeat #3 | |
|---|---|---|---|---|---|---|
| | NAT10 | GAPDH | NAT10 | GAPDH | NAT10 | GAPDH |
| shControl | 17229 | 19808 | 20552 | 20409 | 15772 | 14871 |
| shNAT10-1 | 12822 | 17677 | 11400 | 19932 | 12712 | 16044 |
| shNAT10-2 | 10066 | 16045 | 15965 | 19854 | 11966 | 19696 |
| shNAT10-3 | 8047 | 16652 | 12133 | 17659 | 9431 | 20414 |
| shNAT10-4 | 13140 | 14266 | 22599 | 21848 | 16803 | 16315 |

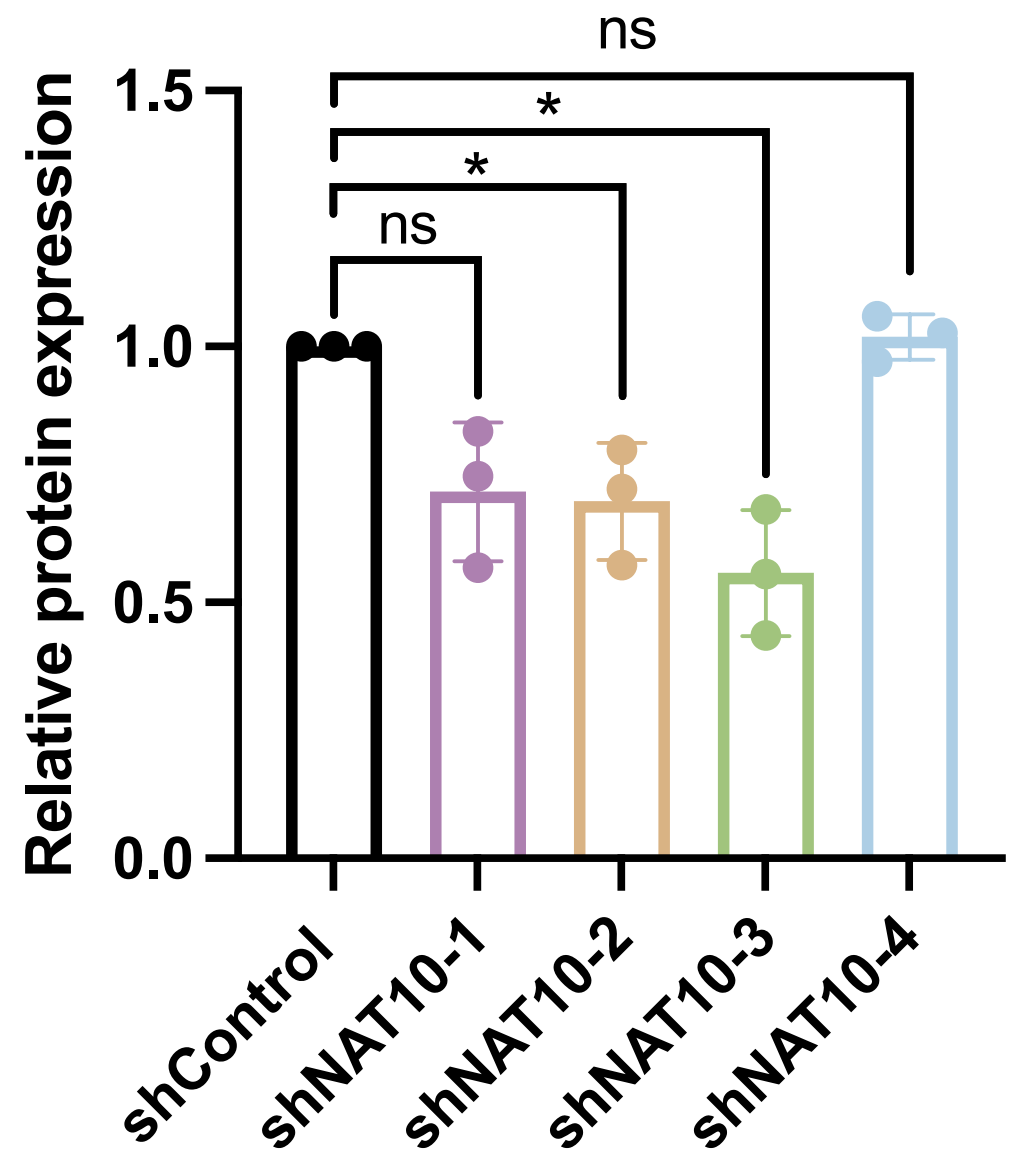

# Figure 3B - KHM-5M

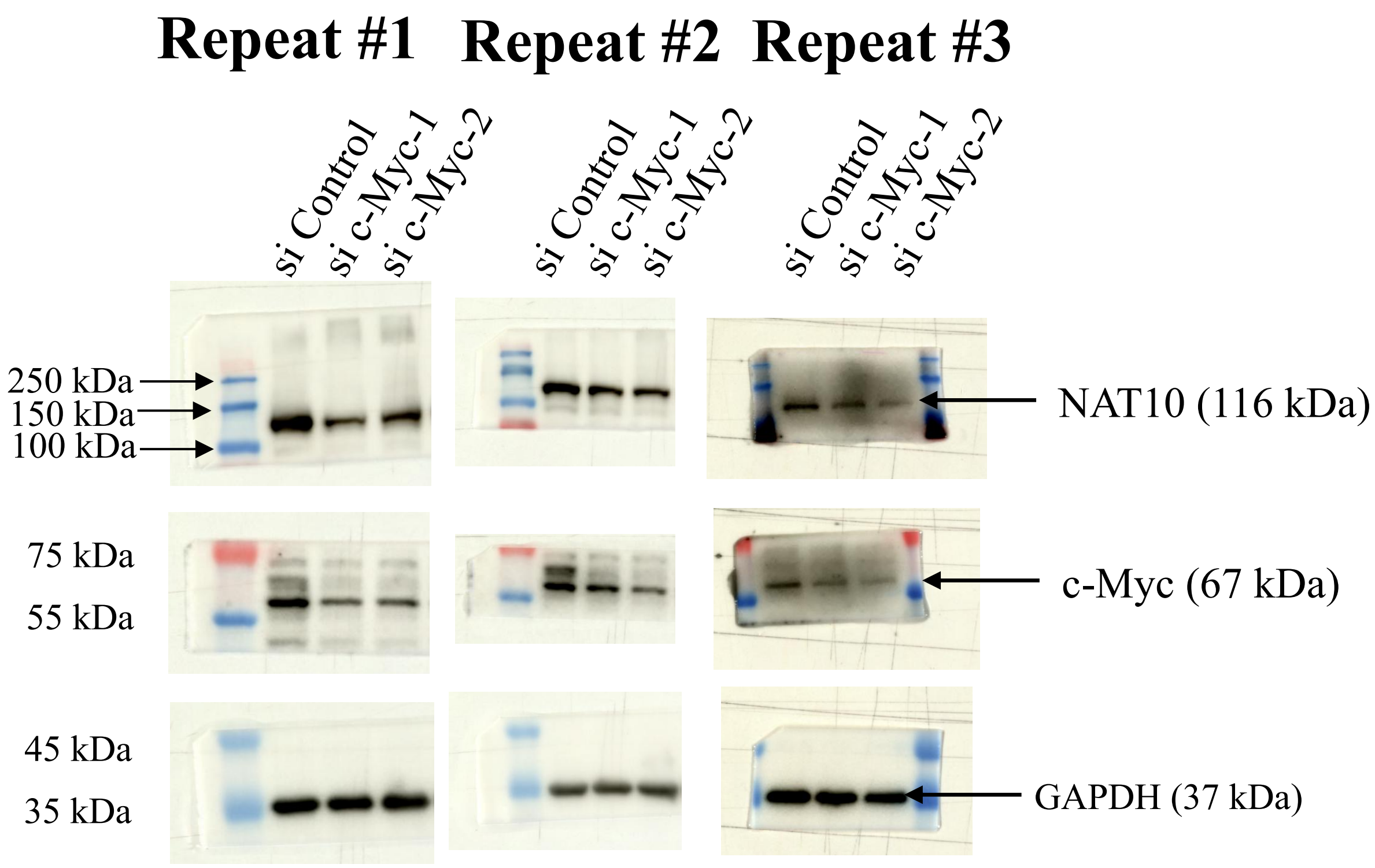


Following transfer, the membrane was cut into strips and probed separately with anti-GAPDH, anti-c-Myc and anti-NAT10 antibodies.

**Western Blot Band Densitometry Measurement**

| Sample | Repeat #1 | | | Repeat #2 | | | Repeat #3 | | |
|---|---|---|---|---|---|---|---|---|---|
| | NAT10 | c-Myc | GAPDH | NAT10 | c-Myc | GAPDH | NAT10 | c-Myc | GAPDH |
| si Control | 23491 | 24946 | 20080 | 14706 | 21393 | 10840 | 12029 | 9260 | 21323 |
| si c-Myc-1 | 12946 | 11837 | 19218 | 10995 | 14964 | 11444 | 9566 | 7203 | 21352 |
| si c-Myc-2 | 16642 | 12198 | 20331 | 9968 | 9924 | 12289 | 6978 | 5561 | 19817 |

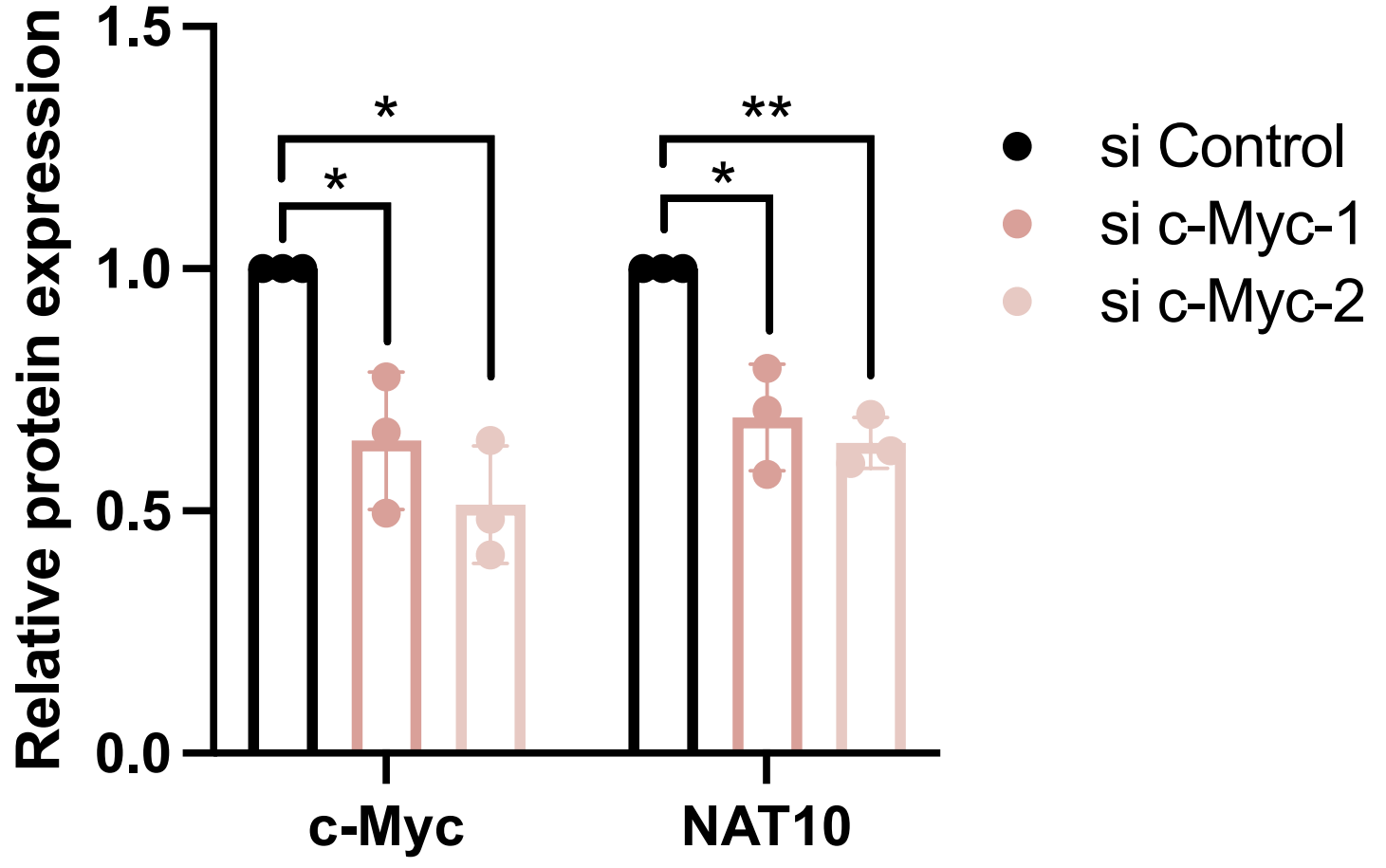

# Figure 3B - FRO

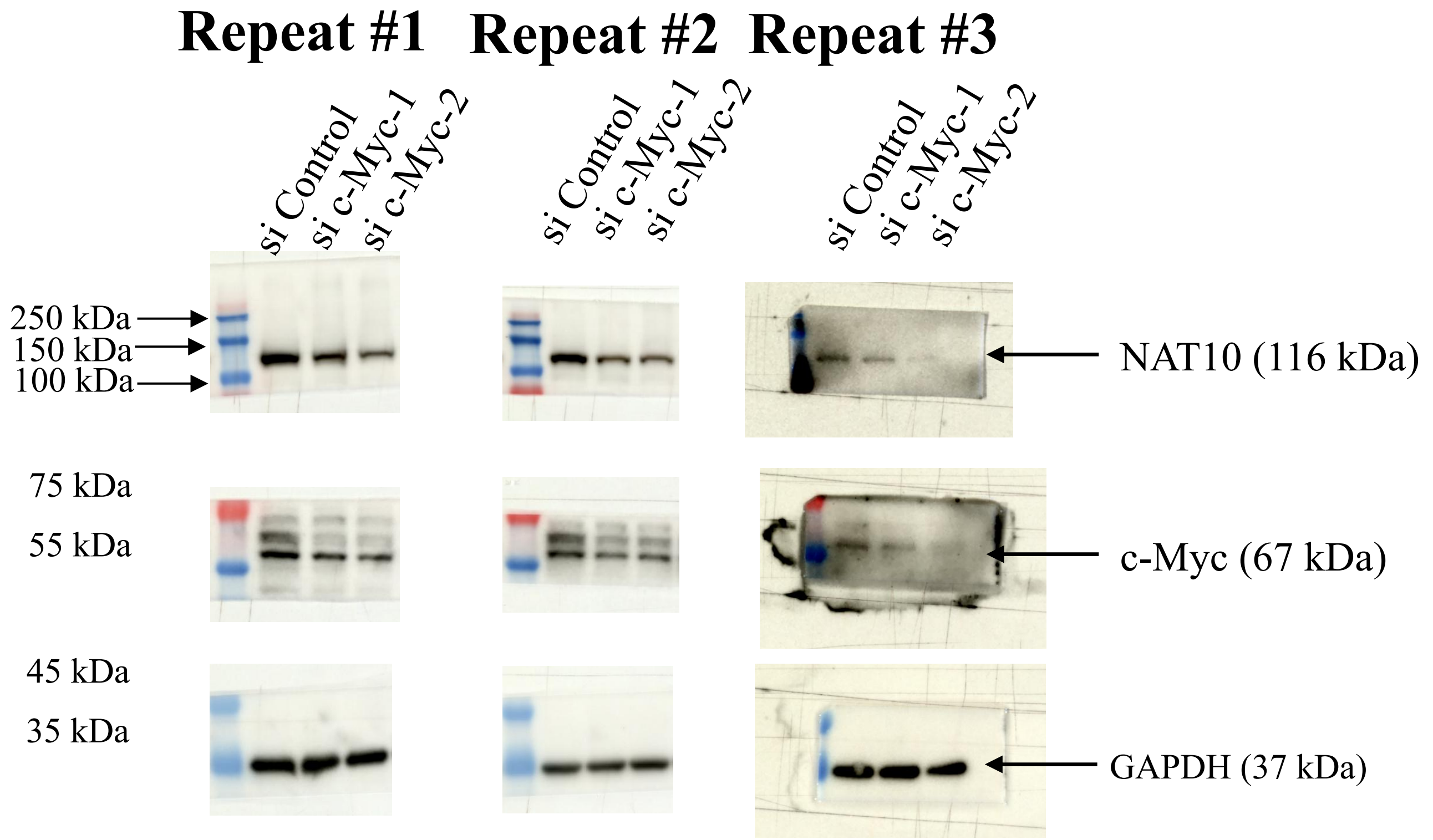


Following transfer, the membrane was cut into strips and probed separately with anti-GAPDH, anti-c-Myc and anti-NAT10 antibodies.

**Western Blot Band Densitometry Measurement**

| Sample | Repeat #1 | | | Repeat #2 | | | Repeat #3 | | |
|---|---|---|---|---|---|---|---|---|---|
| | NAT10 | c-Myc | GAPDH | NAT10 | c-Myc | GAPDH | NAT10 | c-Myc | GAPDH |
| si Control | 15753 | 23004 | 20762 | 11808 | 22264 | 11276 | 9579 | 12898 | 17239 |
| si c-Myc-1 | 11418 | 11983 | 19350 | 7236 | 12864 | 11305 | 4714 | 8014 | 17948 |
| si c-Myc-2 | 7664 | 10879 | 18354 | 6561 | 13360 | 12016 | 2447 | 6679 | 15197 |

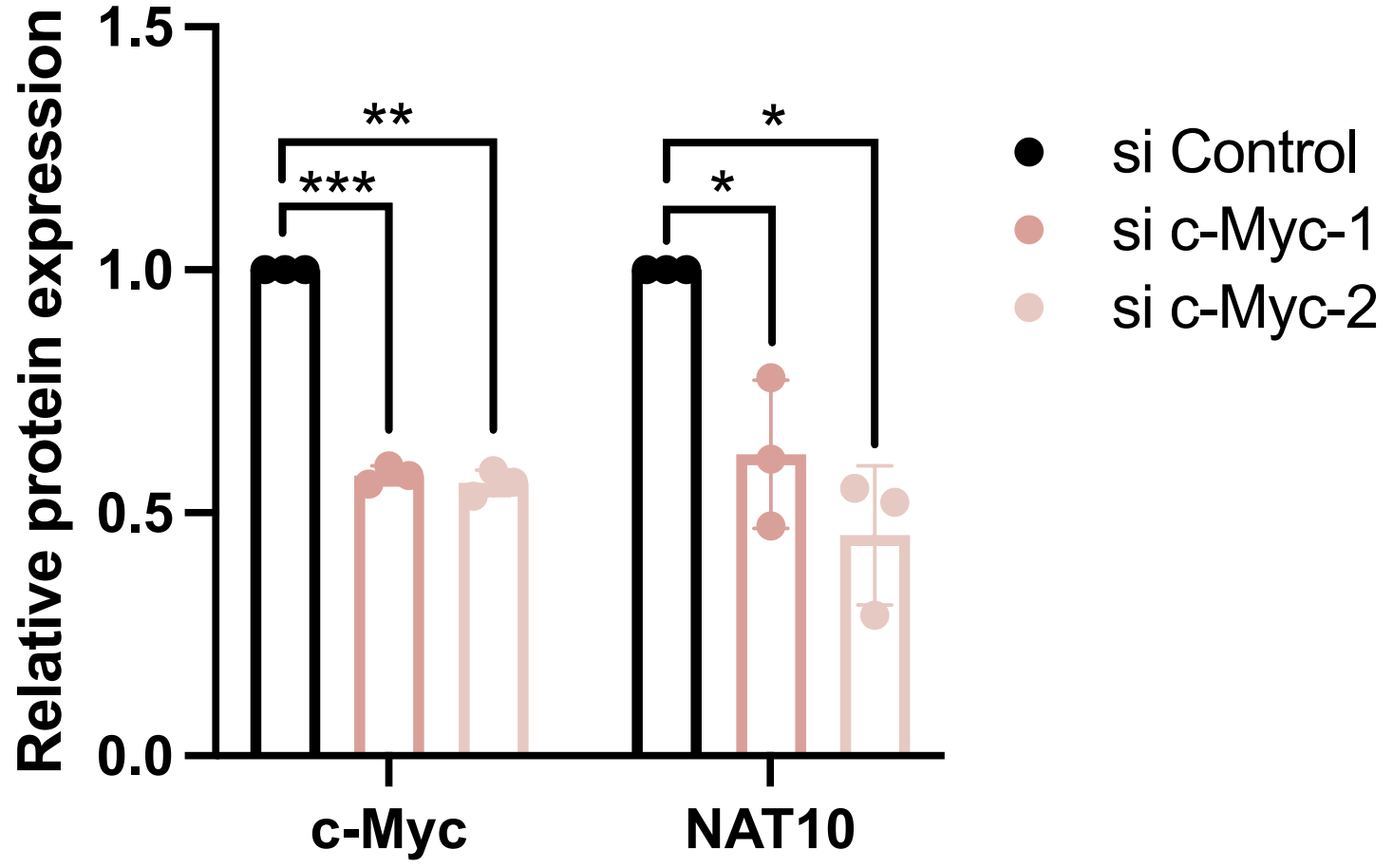

# Figure 4A

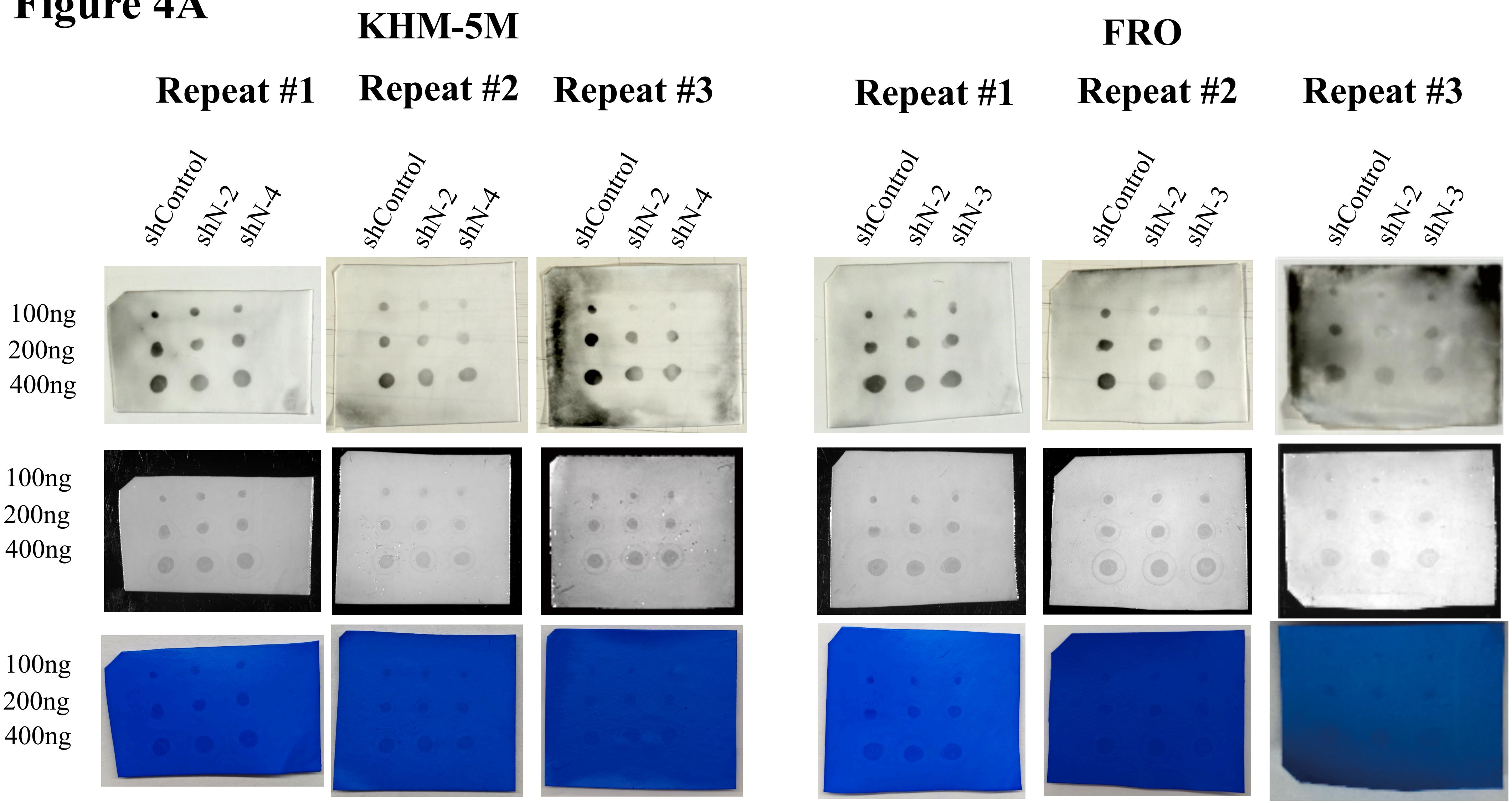


The entire membrane was probed with an antibody against N4-acetylcytidine.

# Figure 4A

## KHM-5M

**Dot Blot Band Densitometry Measurement**

| Sample | Repeat #1 | Repeat #2 | Repeat #3 |
|---|---|---|---|
| | $ac^4c$ | $ac^4c$ | $ac^4c$ |
| shControl | 14976 | 9543 | 15659 |
| shN-2 | 7451 | 4308 | 5700 |
| shN-4 | 6655 | 4201 | 4187 |

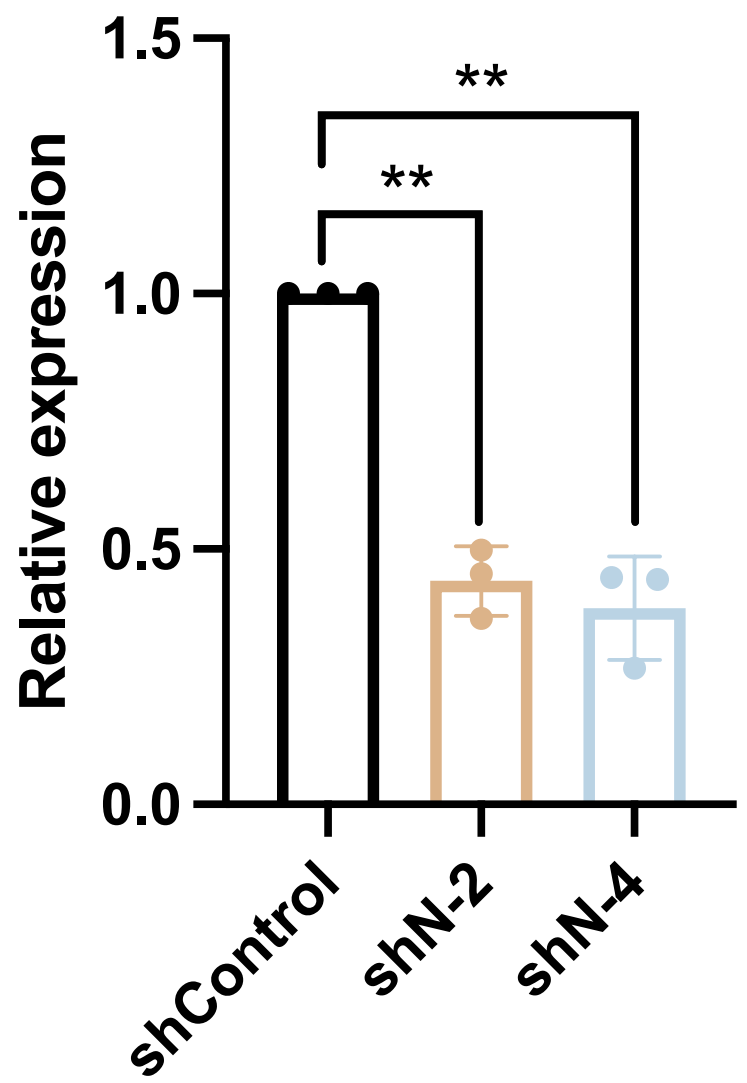


## FRO

**Dot Blot Band Densitometry Measurement**

| Sample | Repeat #1 | Repeat #2 | Repeat #3 |
|---|---|---|---|
| | $ac^4c$ | $ac^4c$ | $ac^4c$ |
| shControl | 17952 | 14703 | 16635 |
| shN-2 | 8332 | 4571 | 5958 |
| shN-3 | 8852 | 5352 | 6345 |

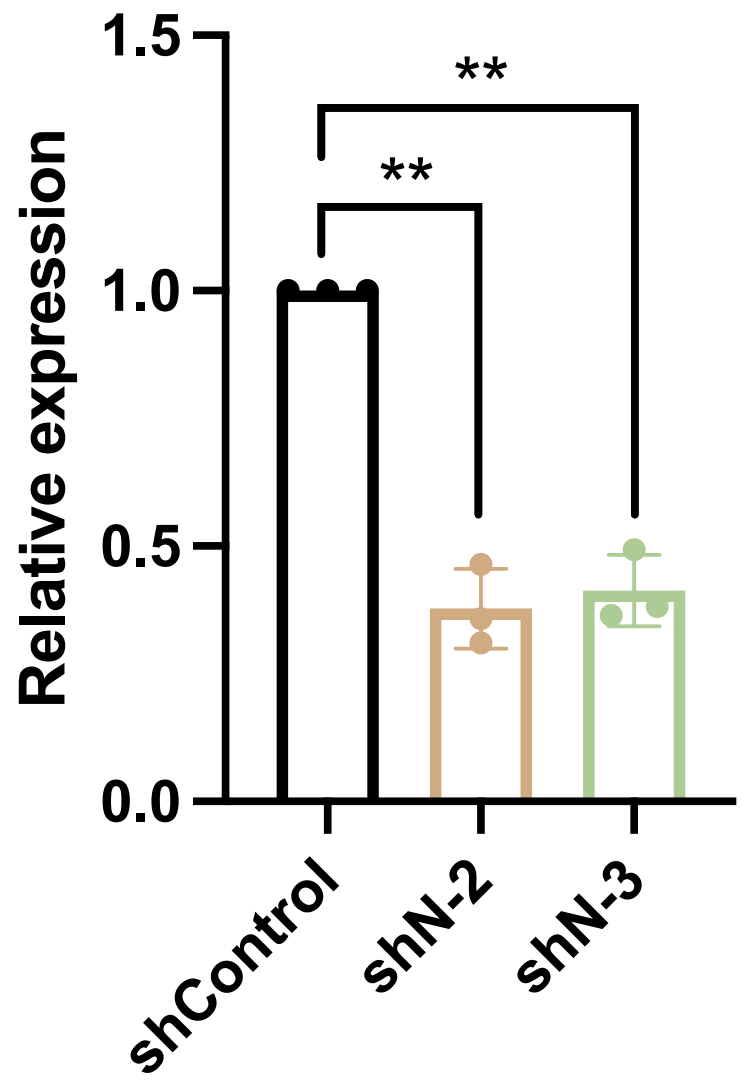

# Figure 4A- 8305C

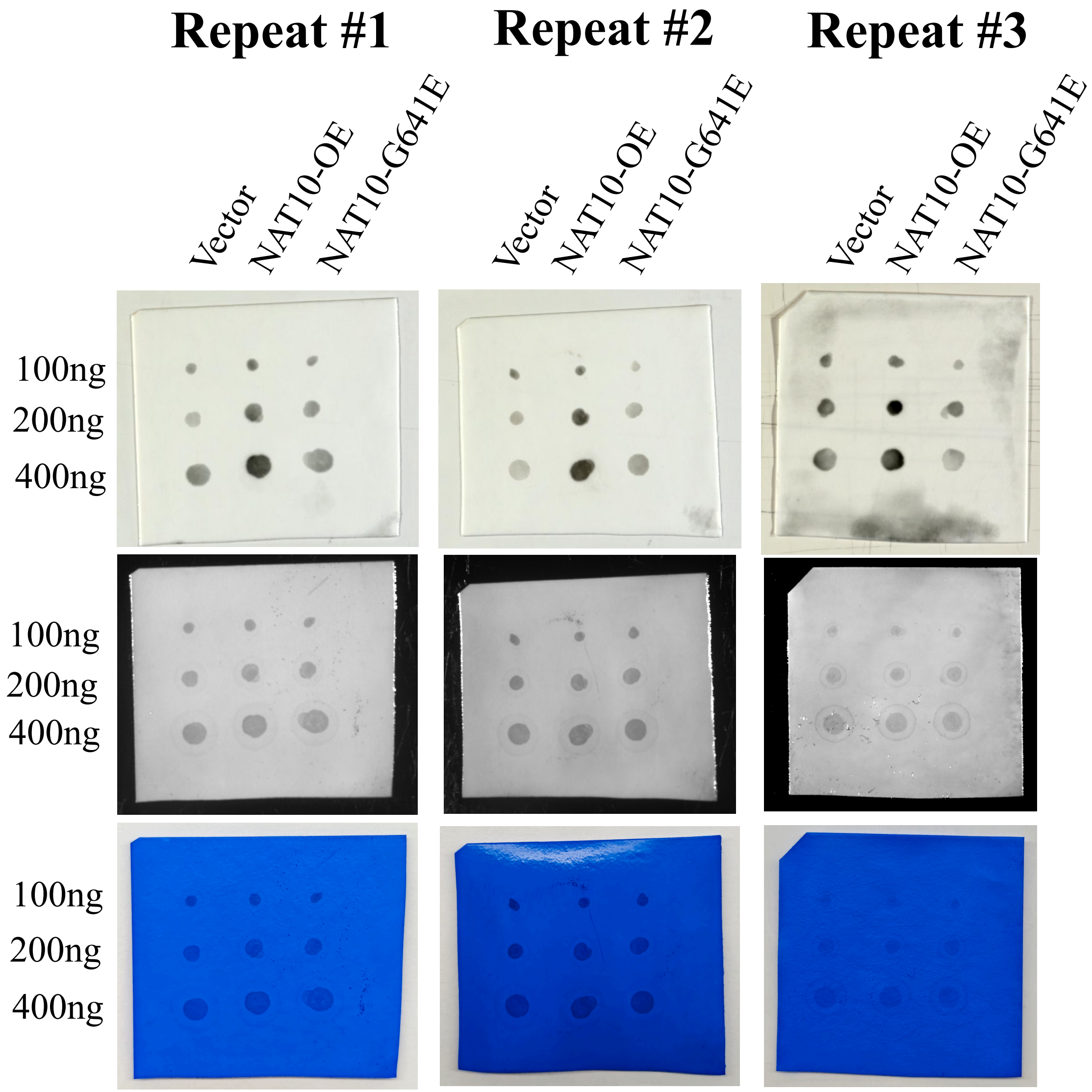


The entire membrane was probed with an antibody against N4-acetylcytidine.

**Dot Blot Band Densitometry Measurement**

| Sample | Repeat #1 | Repeat #2 | Repeat #3 |
|---|---|---|---|
| | $ac^4c$ | $ac^4c$ | $ac^4c$ |
| Vector | 5352 | 2282 | 5172 |
| NAT10-OE | 13195 | 8630 | 12174 |
| NAT10-G641E | 6241 | 3850 | 3297 |

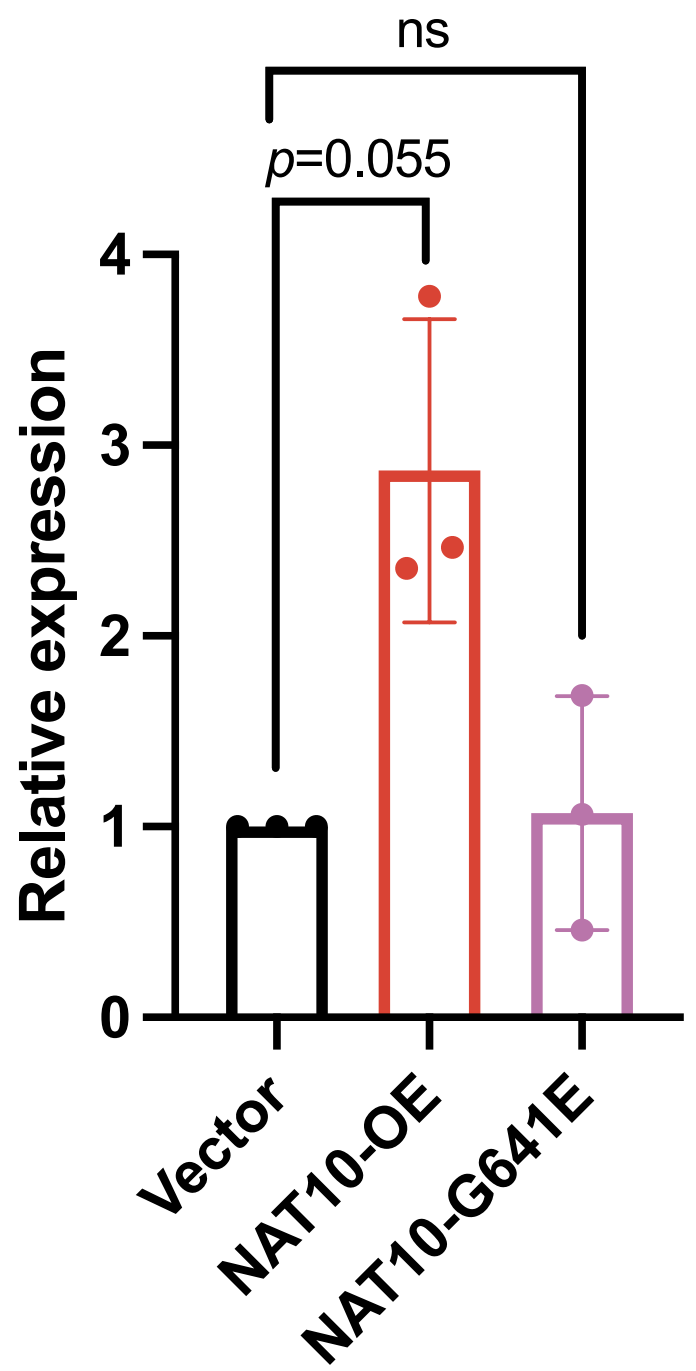

# Figure 4A -remodelin

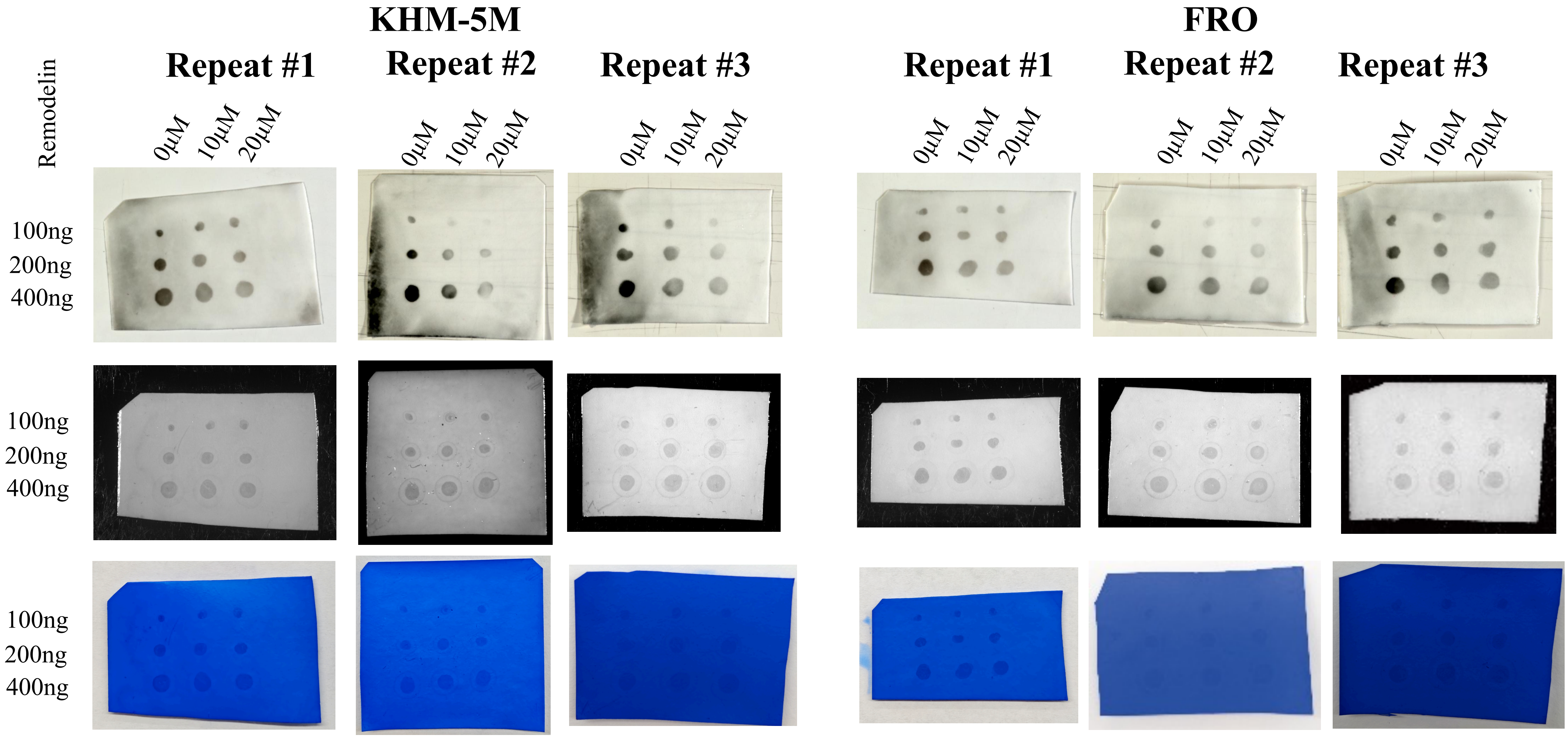


The entire membrane was probed with an antibody against N4-acetylcytidine.

# Figure 4A -remodelin

## KHM-5M

**Dot Blot Band Densitometry Measurement**

| Sample | Repeat #1 | Repeat #2 | Repeat #3 |
| --- | --- | --- | --- |
| | $ac^4c$ | $ac^4c$ | $ac^4c$ |
| 0μM | 17565 | 18002 | 21807 |
| 10μM | 8661 | 8726 | 9177 |
| 20μM | 5242 | 3307 | 4785 |

## FRO

**Dot Blot Band Densitometry Measurement**

| Sample | Repeat #1 | Repeat #2 | Repeat #3 |
| --- | --- | --- | --- |
| | $ac^4c$ | $ac^4c$ | $ac^4c$ |
| 0μM | 17032 | 19002 | 21594 |
| 10μM | 7320 | 10270 | 10783 |
| 20μM | 5826 | 5949 | 7415 |

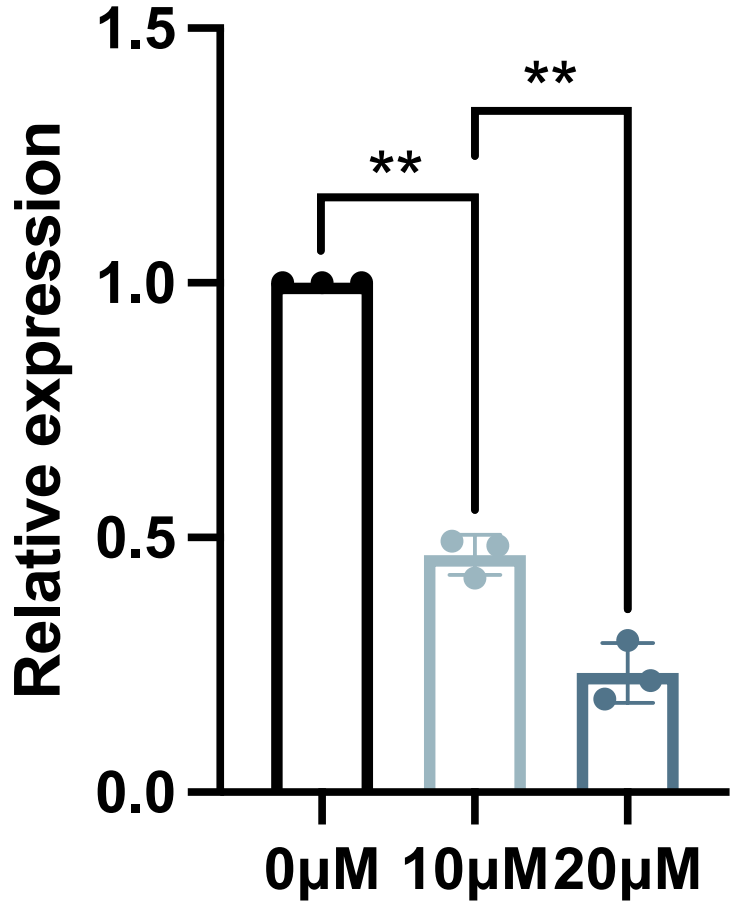


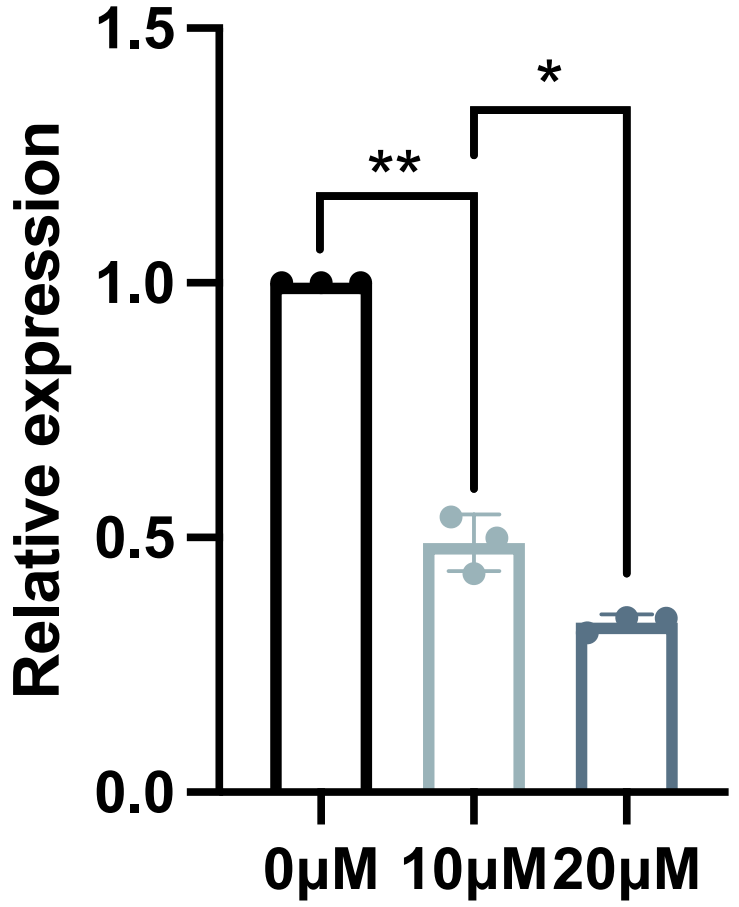

# Figure 4H - KHM-5M

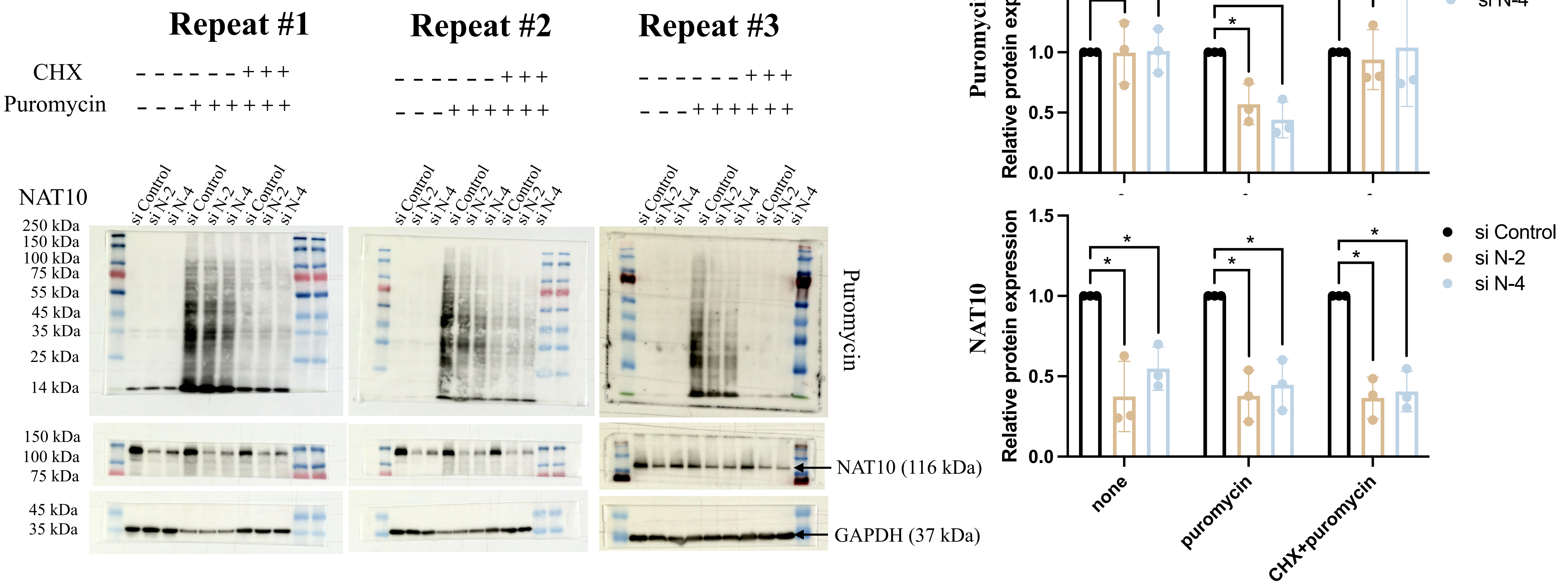


The membrane was entirely transfected and probed with puromycin antibody. After detected, the membrane was washed, cut, and subsequently probed with anti-NAT10 and anti-GAPDH antibodies.

**Western Blot Band Densitometry Measurement**

| Sample | | Repeat #1 | | | Repeat #2 | | | Repeat #3 | | |
|---|---|---|---|---|---|---|---|---|---|---|
| | | Puromycin | NAT10 | GAPDH | Puromycin | NAT10 | GAPDH | Puromycin | NAT10 | GAPDH |
| None | si Control | 7315 | 9965 | 7620 | 3171 | 8573 | 7063 | 10282 | 16426 | 19841 |
| | si N-2 | 5886 | 2820 | 8447 | 2910 | 1850 | 6329 | 11232 | 9073 | 17471 |
| | si N-4 | 6279 | 4541 | 7890 | 2730 | 3120 | 5094 | 8597 | 9495 | 16393 |
| Puromycin | si Control | 96508 | 9620 | 3771 | 76746 | 7198 | 2948 | 151374 | 12148 | 15872 |
| | si N-2 | 62865 | 3131 | 3259 | 46127 | 1784 | 3357 | 70876 | 7184 | 17435 |
| | si N-4 | 49823 | 3676 | 3190 | 33579 | 2699 | 3847 | 60395 | 7791 | 16905 |
| CHX+ Puromycin | si Control | 25249 | 7131 | 6606 | 14534 | 6554 | 5197 | 9002 | 12095 | 18961 |
| | si N-2 | 20084 | 2710 | 6561 | 13773 | 1789 | 6225 | 10894 | 5792 | 18715 |
| | si N-4 | 19093 | 3977 | 6742 | 12687 | 2239 | 5868 | 15682 | 4851 | 20622 |

# Figure 4H - FRO

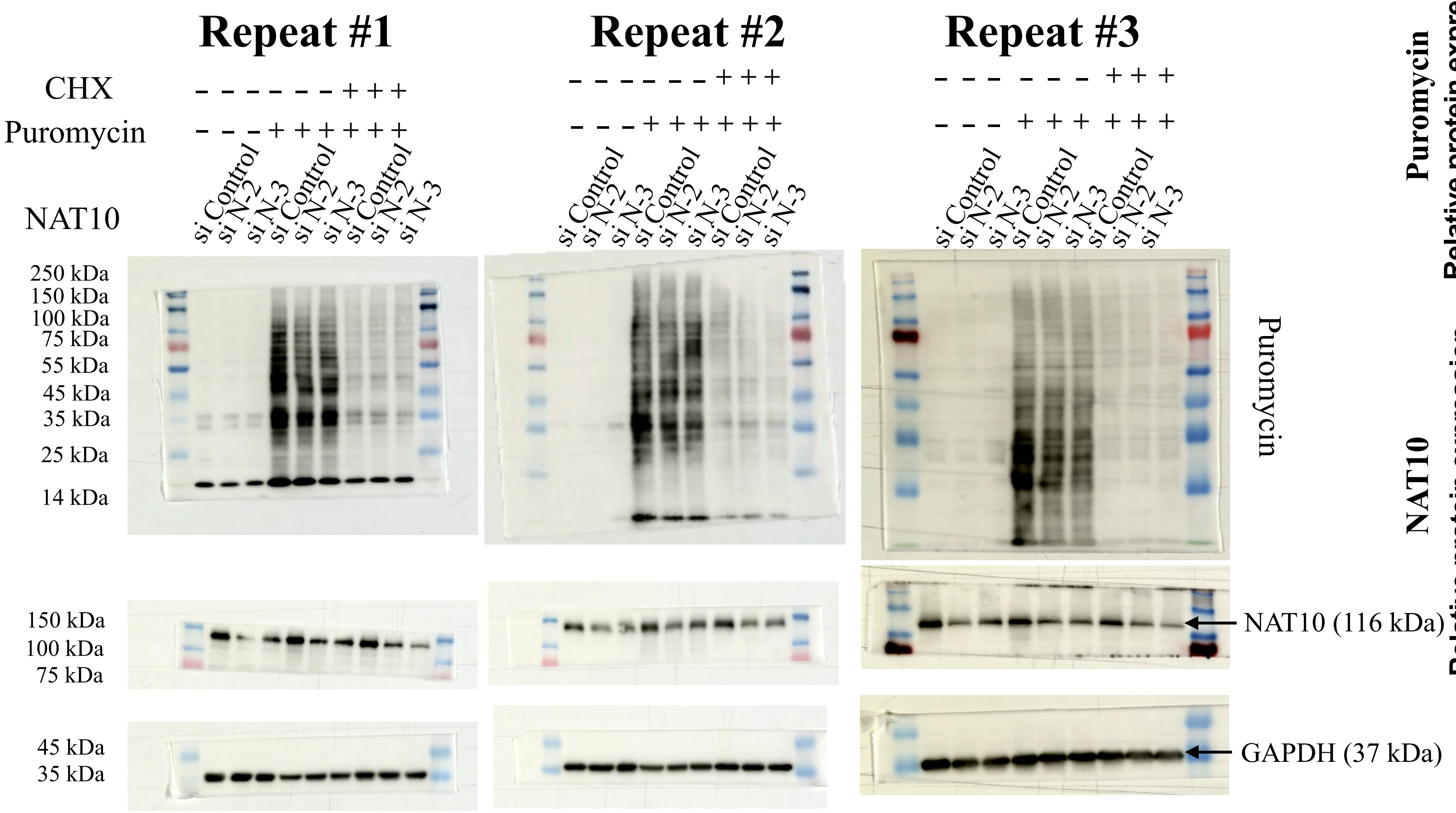


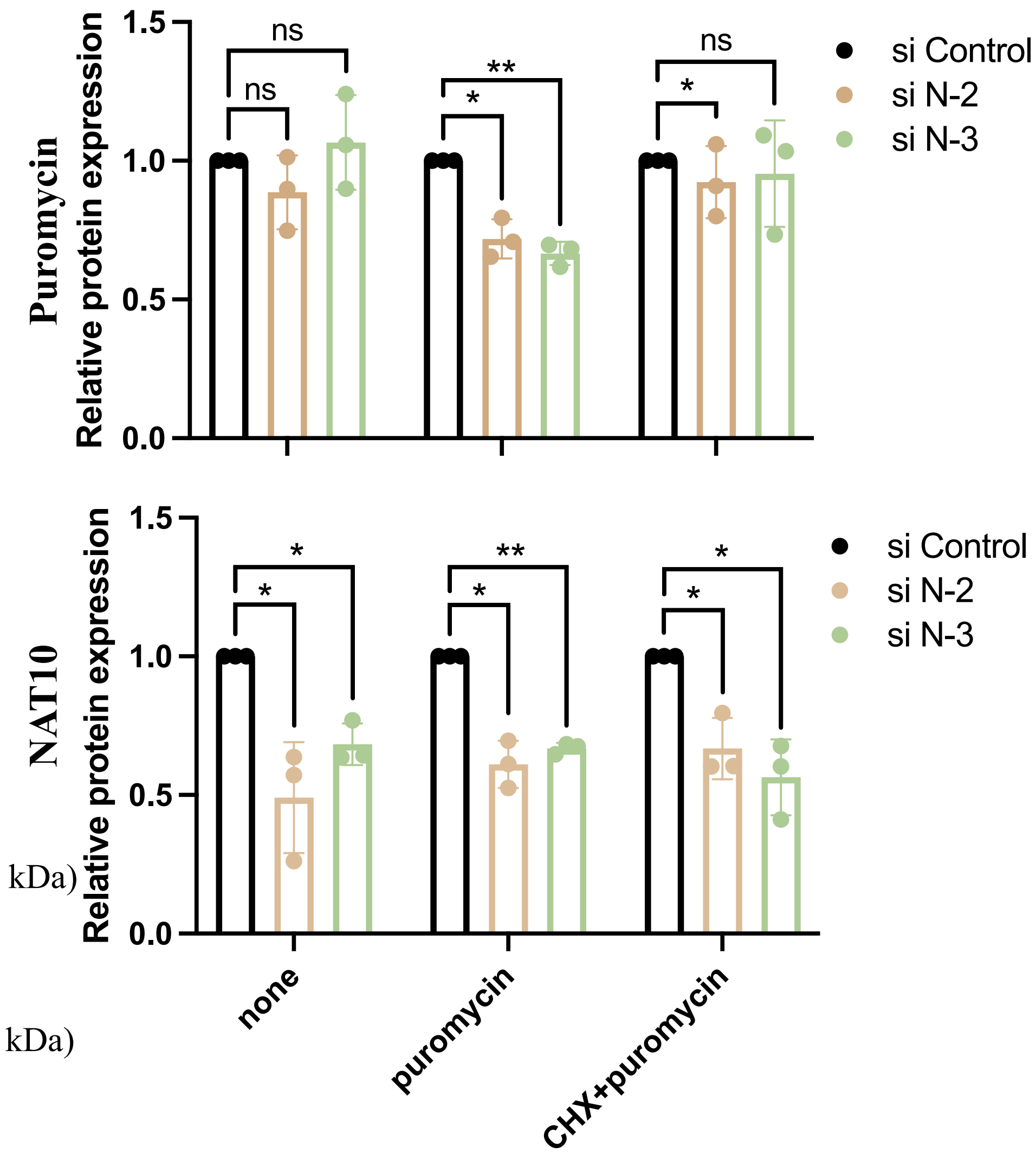


The membrane was entirely transfected and probed with puromycin antibody. After detected, the membrane was washed, cut, and subsequently probed with anti-NAT10 and anti-GAPDH antibodies.

**Western Blot Band Densitometry Measurement**

| Sample | | Repeat #1 | | | Repeat #2 | | | Repeat #3 | | |
|---|---|---|---|---|---|---|---|---|---|---|
| | | Puromycin | NAT10 | GAPDH | Puromycin | NAT10 | GAPDH | Puromycin | NAT10 | GAPDH |
| None | si Control | 10506 | 8115 | 8051 | 4484 | 5953 | 7705 | 15817 | 14650 | 23118 |
| | si N-2 | 7650 | 2340 | 8855 | 3672 | 4023 | 8178 | 10354 | 6984 | 19254 |
| | si N-3 | 9625 | 5310 | 8199 | 5162 | 4123 | 8389 | 16666 | 9569 | 19627 |
| Puromycin | si Control | 101470 | 8621 | 6015 | 189266 | 6837 | 6044 | 182103 | 12975 | 23775 |
| | si N-2 | 77966 | 5323 | 7067 | 141926 | 4432 | 6402 | 139046 | 8675 | 22859 |
| | si N-3 | 76753 | 6173 | 6663 | 136429 | 5451 | 7050 | 118923 | 8213 | 22283 |
| CHX+ Puromycin | si Control | 21527 | 8280 | 7976 | 38990 | 7311 | 8422 | 24728 | 11321 | 21714 |
| | si N-2 | 22750 | 4980 | 7959 | 29724 | 4208 | 8016 | 18643 | 7466 | 17997 |
| | si N-3 | 21493 | 3120 | 7292 | 28229 | 4882 | 8303 | 20597 | 5504 | 17491 |

# Figure 4H – 8305C

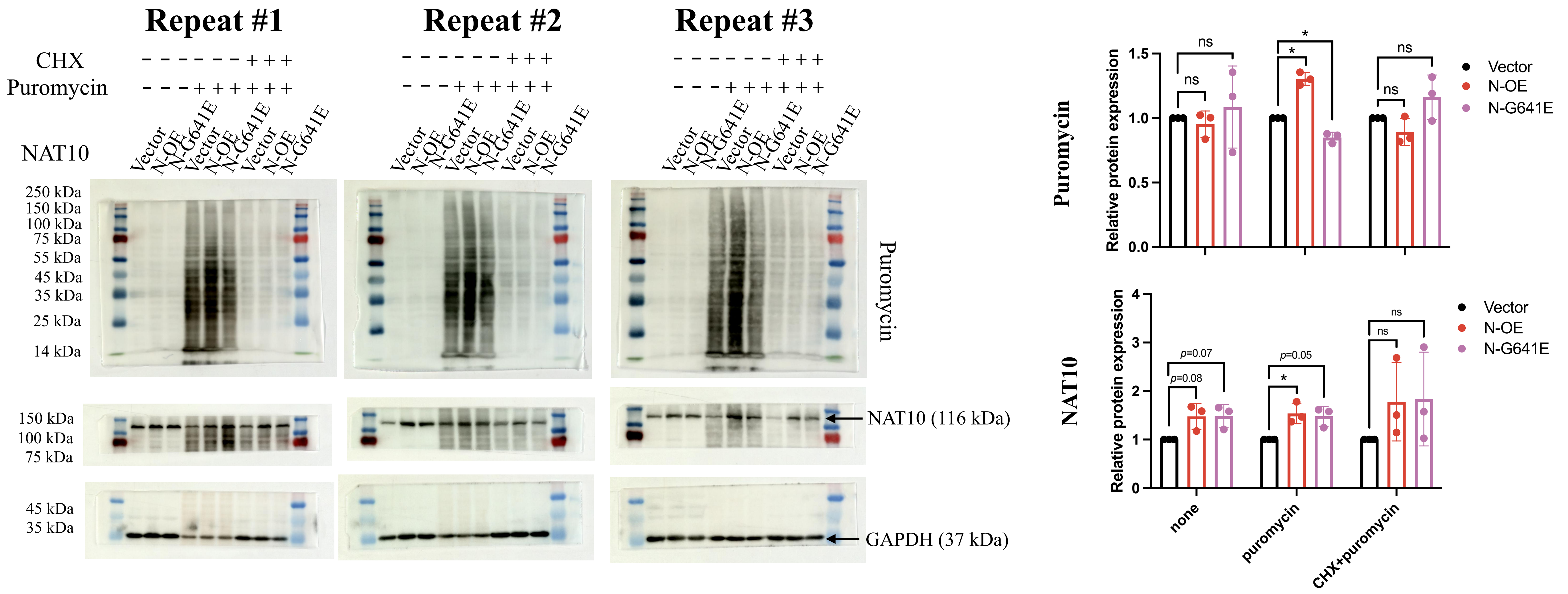


The membrane was entirely transfected and probed with puromycin antibody. After detected, the membrane was washed, cut, and subsequently probed with anti-NAT10 and anti-GAPDH antibodies.

**Western Blot Band Densitometry Measurement**

| Sample | | Repeat #1 | | | Repeat #2 | | | Repeat #3 | | |
|---|---|---|---|---|---|---|---|---|---|---|
| | | Puromycin | NAT10 | GAPDH | Puromycin | NAT10 | GAPDH | Puromycin | NAT10 | GAPDH |
| None | Vector | 10115 | 5244 | 8275 | 12632 | 3222 | 6869 | 8257 | 3808 | 10207 |
| | N-OE | 9261 | 6436 | 8649 | 16039 | 6829 | 8697 | 7442 | 5315 | 8996 |
| | N-G641E | 7435 | 6341 | 8279 | 21042 | 6267 | 8439 | 7120 | 4666 | 7535 |
| Puromycin | Vector | 106669 | 4666 | 5172 | 107811 | 6690 | 6280 | 106530 | 3973 | 8526 |
| | N-OE | 138720 | 6571 | 4961 | 127266 | 8602 | 5899 | 151972 | 7734 | 9339 |
| | N-G641E | 82168 | 7235 | 4963 | 96263 | 8409 | 6336 | 85057 | 5704 | 7749 |
| CHX+ Puromycin | Vector | 16525 | 4259 | 7592 | 6016 | 4812 | 9041 | 22907 | 1822 | 9242 |
| | N-OE | 13201 | 6056 | 7178 | 6182 | 5589 | 9173 | 17828 | 4644 | 8773 |
| | N-G641E | 15125 | 5159 | 5839 | 8198 | 5095 | 9345 | 18405 | 4351 | 7595 |

# Figure 4J – KHM-5M

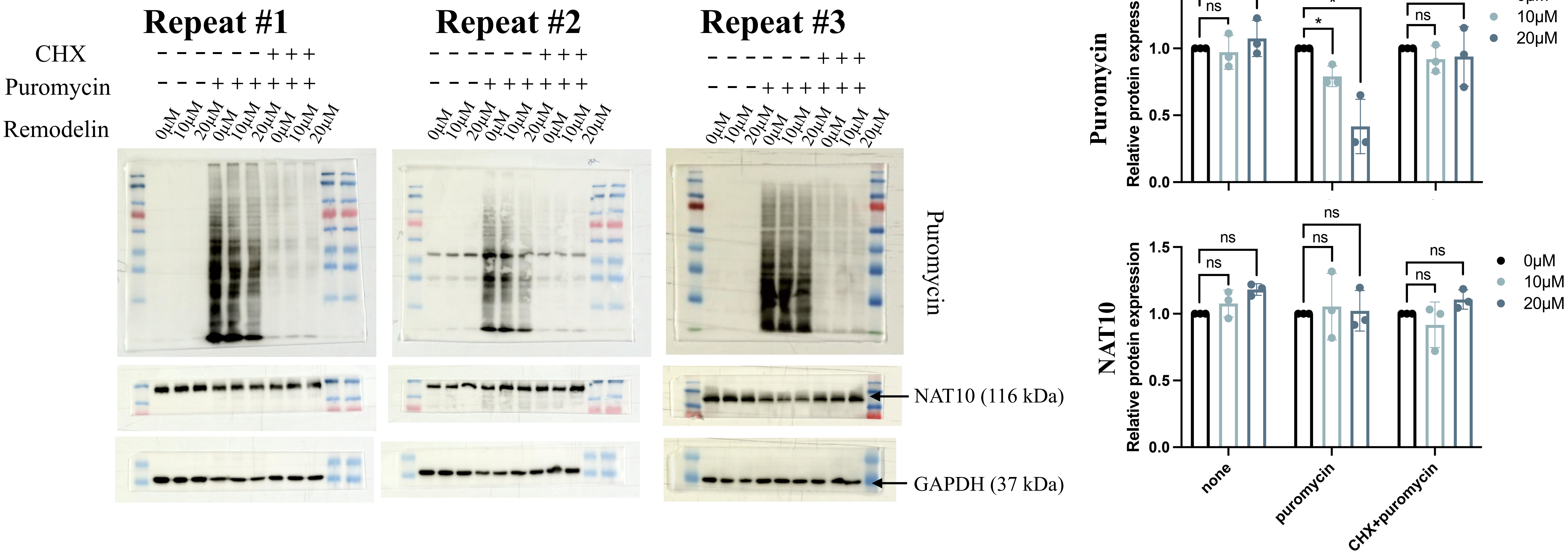


The membrane was entirely transfected and probed with puromycin antibody. After detected, the membrane was washed, cut, and subsequently probed with anti-NAT10 and anti-GAPDH antibodies.

**Western Blot Band Densitometry Measurement**

| Sample | | Repeat #1 | | | Repeat #2 | | | Repeat #3 | | |
|---|---|---|---|---|---|---|---|---|---|---|
| | | Puromycin | NAT10 | GAPDH | Puromycin | NAT10 | GAPDH | Puromycin | NAT10 | GAPDH |
| None | 0μM | 3129 | 7922 | 8915 | 5048 | 4369 | 7383 | 6827 | 20104 | 13372 |
| | 10μM | 2950 | 7807 | 7556 | 4589 | 4094 | 7169 | 4915 | 18430 | 11120 |
| | 20μM | 2846 | 7901 | 7839 | 5393 | 4644 | 6436 | 5733 | 17241 | 9624 |
| Puromycin | 0μM | 119033 | 6411 | 5940 | 63036 | 4777 | 4252 | 212663 | 14828 | 13299 |
| | 10μM | 94398 | 6402 | 5782 | 49016 | 6551 | 4429 | 183653 | 11914 | 13042 |
| | 20μM | 67421 | 6236 | 4831 | 25341 | 6128 | 5721 | 121995 | 11975 | 11715 |
| CHX+ Puromycin | 0μM | 13617 | 5993 | 6138 | 5732 | 5932 | 5765 | 22285 | 15696 | 11853 |
| | 10μM | 12127 | 6642 | 6595 | 6266 | 5190 | 6997 | 23916 | 16392 | 12382 |
| | 20μM | 10099 | 6534 | 6410 | 6128 | 7245 | 6456 | 25012 | 18104 | 11532 |

# Figure 4J – FRO

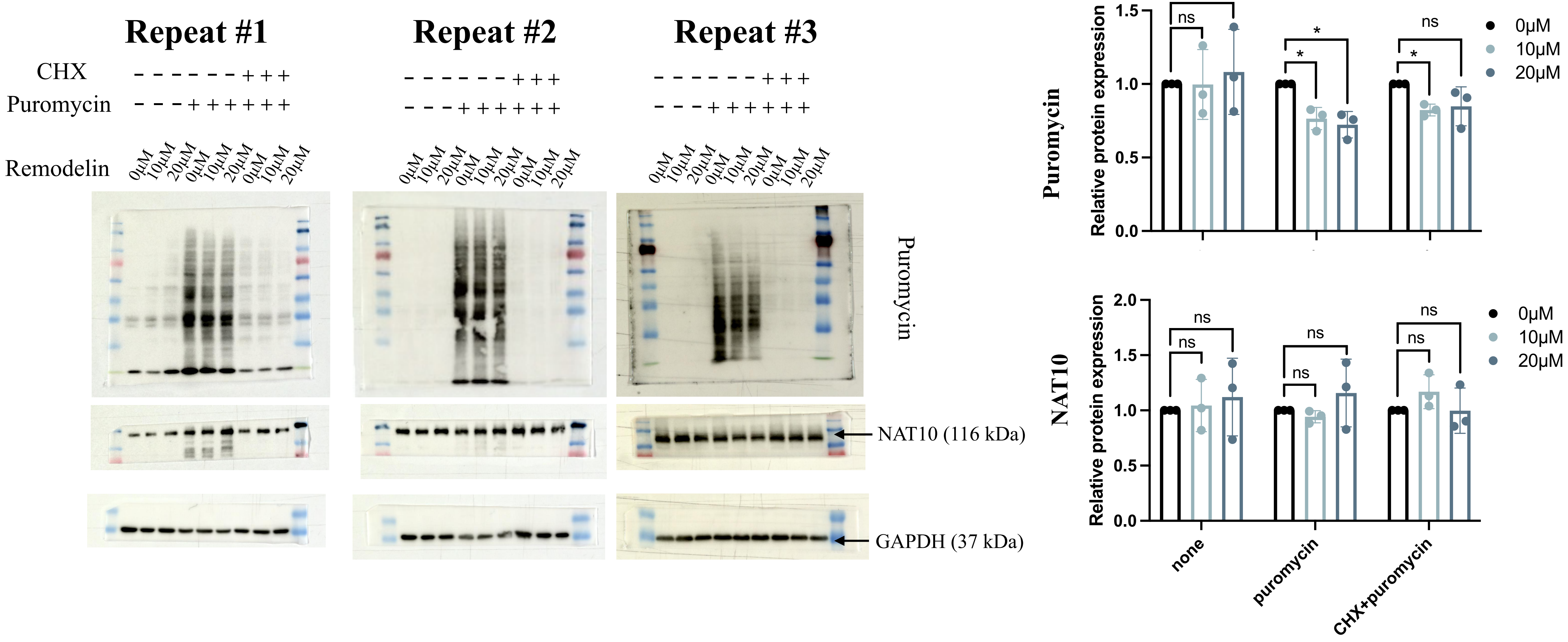


The membrane was entirely transfected and probed with puromycin antibody. After detected, the membrane was washed, cut, and subsequently probed with anti-NAT10 and anti-GAPDH antibodies.

**Western Blot Band Densitometry Measurement**

| Sample | | Repeat #1 | | | Repeat #2 | | | Repeat #3 | | |
|---|---|---|---|---|---|---|---|---|---|---|
| | | Puromycin | NAT10 | GAPDH | Puromycin | NAT10 | GAPDH | Puromycin | NAT10 | GAPDH |
| None | 0μM | 7381 | 2225 | 7994 | 7920 | 5258 | 6223 | 6882 | 19866 | 11865 |
| | 10μM | 7204 | 2225 | 6186 | 6349 | 4636 | 5374 | 6850 | 20274 | 14748 |
| | 20μM | 8763 | 2710 | 6839 | 5595 | 5505 | 5410 | 8993 | 18248 | 14814 |
| Puromycin | 0μM | 75882 | 4456 | 4970 | 177100 | 4771 | 3921 | 166595 | 17296 | 15996 |
| | 10μM | 62989 | 4654 | 5230 | 153488 | 4766 | 4115 | 124057 | 15383 | 16109 |
| | 20μM | 52235 | 5559 | 4326 | 132659 | 5714 | 3873 | 108329 | 14997 | 16756 |
| CHX+ Puromycin | 0μM | 12963 | 3063 | 5279 | 10636 | 5955 | 6376 | 12404 | 18607 | 16551 |
| | 10μM | 12113 | 4442 | 5720 | 8293 | 5848 | 6028 | 9243 | 17884 | 14068 |
| | 20μM | 11157 | 3238 | 6519 | 8429 | 4525 | 5367 | 8450 | 17212 | 12427 |

# Figure 5J

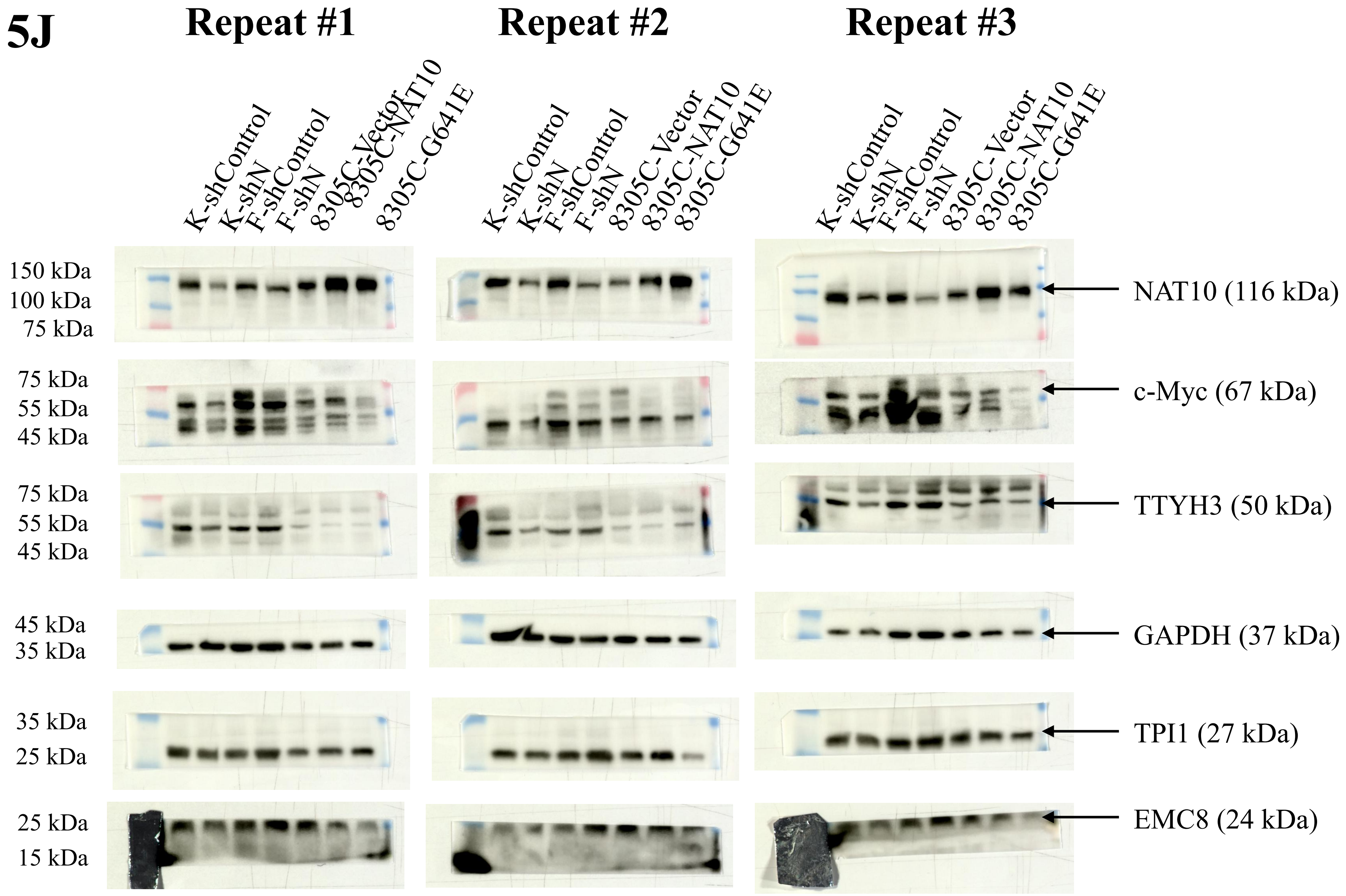


Following transfer, the membrane was cut into strips and probed separately with anti-GAPDH, anti-c-Myc, anti-TPI1, anti-EMC8 and anti-NAT10 antibodies. After detected, the strip corresponding to c-Myc was stripped and re-probed with an anti-TTYH3 antibody.

## Repeat #1

**Western Blot Band Densitometry Measurement**

| Sample | NAT10 | c-Myc | TTYH3 | GAPDH | TPI1 | EMC8 |
|---|---|---|---|---|---|---|
| K-shControl | 23061 | 49377 | 15505 | 22295 | 24677 | 26031 |
| K-shN | 11276 | 31567 | 10257 | 25352 | 20451 | 20636 |
| F-shControl | 21897 | 77691 | 13814 | 25828 | 20921 | 25394 |
| F-shN | 17814 | 60114 | 16154 | 28526 | 26236 | 29244 |
| 8305C-Vector | 23188 | 37497 | 4256 | 18723 | 15631 | 25097 |
| 8305C-NAT10 | 39434 | 40008 | 1956 | 17165 | 18505 | 16877 |
| 8305C-G641E | 35033 | 20064 | 2177 | 17855 | 19522 | 13011 |

## Repeat #2

**Western Blot Band Densitometry Measurement**

| Sample | NAT10 | c-Myc | TTYH3 | GAPDH | TPI1 | EMC8 |
|---|---|---|---|---|---|---|
| K-shControl | 15102 | 13635 | 11692 | 17424 | 11093 | 8448 |
| K-shN | 8245 | 8673 | 4937 | 14668 | 9669 | 7312 |
| F-shControl | 14887 | 17218 | 7042 | 12690 | 11813 | 9092 |
| F-shN | 8643 | 14277 | 8638 | 12601 | 15104 | 12815 |
| 8305C-Vector | 8115 | 9780 | 3090 | 12352 | 10785 | 12862 |
| 8305C-NAT10 | 13216 | 11031 | 2484 | 11232 | 13069 | 11963 |
| 8305C-G641E | 15663 | 8275 | 4581 | 9074 | 4687 | 8210 |

# Repeat #3

**Western Blot Band Densitometry Measurement**

| | NAT10 | c-Myc | TTYH3 | GAPDH | TPI1 | EMC8 |
|---|---|---|---|---|---|---|
| K-shControl | 16970 | 35044 | 8839 | 6260 | 12283 | 11550 |
| K-shN | 10725 | 27834 | 5230 | 6667 | 11381 | 9402 |
| F-shControl | 14559 | 55165 | 10464 | 10664 | 14323 | 11472 |
| F-shN | 7868 | 38883 | 12294 | 12372 | 16438 | 13150 |
| 8305C-Vector | 11901 | 19346 | 6160 | 8612 | 12280 | 10370 |
| 8305C-NAT10 | 20731 | 21032 | 8066 | 8164 | 11424 | 8836 |
| 8305C-G641E | 14124 | 7514 | 5269 | 6220 | 9226 | 6674 |

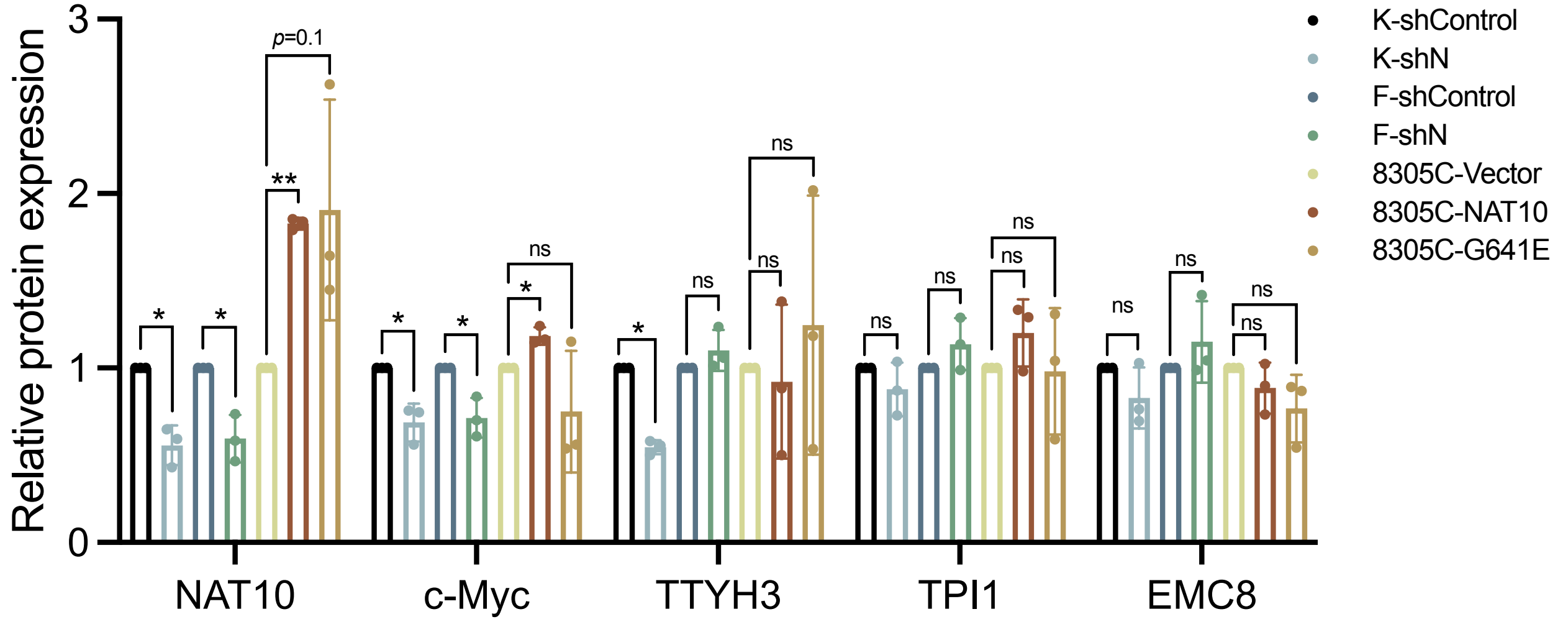

# Figure 6A – KHM-5M

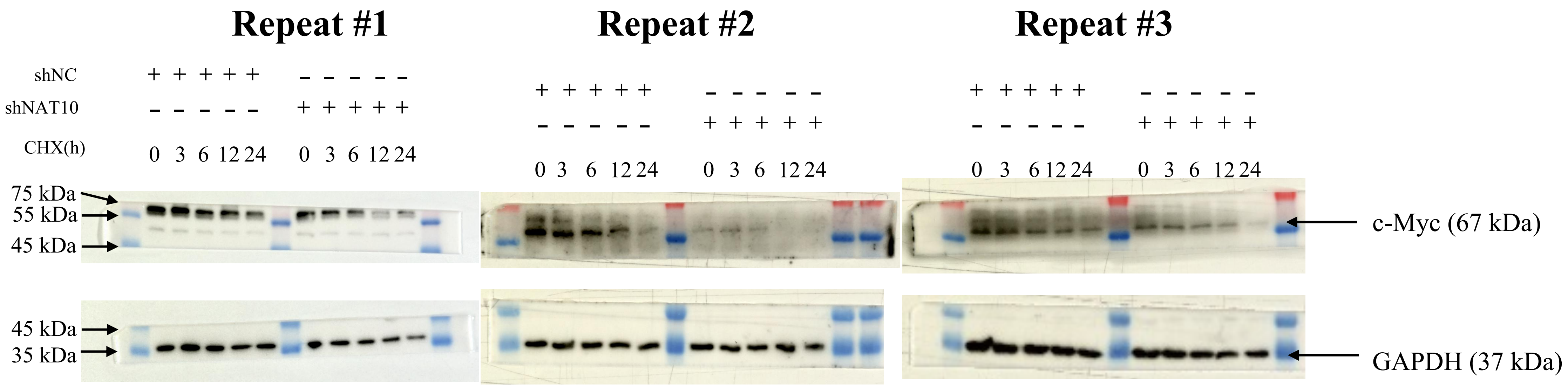


Following transfer, the membrane was cut into strips and probed separately with anti-GAPDH and anti-c-Myc antibodies.

# Figure 6A – KHM-5M

**Western Blot Band Densitometry Measurement**

| Sample | | Repeat #1 | | Repeat #2 | | Repeat #3 | |
|---|---|---|---|---|---|---|---|
| | | c-Myc | GAPDH | c-Myc | GAPDH | c-Myc | GAPDH |
| shNC | 0h | 12933 | 9179 | 29969 | 11907 | 28394 | 21670 |
| | 3h | 13280 | 10870 | 27466 | 11607 | 26366 | 20069 |
| | 6h | 10056 | 9746 | 23941 | 10716 | 23099 | 18396 |
| | 12h | 10298 | 8096 | 21666 | 9488 | 21934 | 17838 |
| | 24h | 6747 | 9053 | 15350 | 9525 | 14296 | 14944 |
| shNAT10 | 0h | 8288 | 7824 | 15110 | 11711 | 18070 | 17976 |
| | 3h | 8052 | 7623 | 15313 | 9519 | 16478 | 16738 |
| | 6h | 6034 | 6329 | 14153 | 9673 | 13037 | 14477 |
| | 12h | 3052 | 6049 | 11762 | 11577 | 10294 | 11939 |
| | 24h | 3239 | 4461 | 10258 | 9350 | 5124 | 12926 |

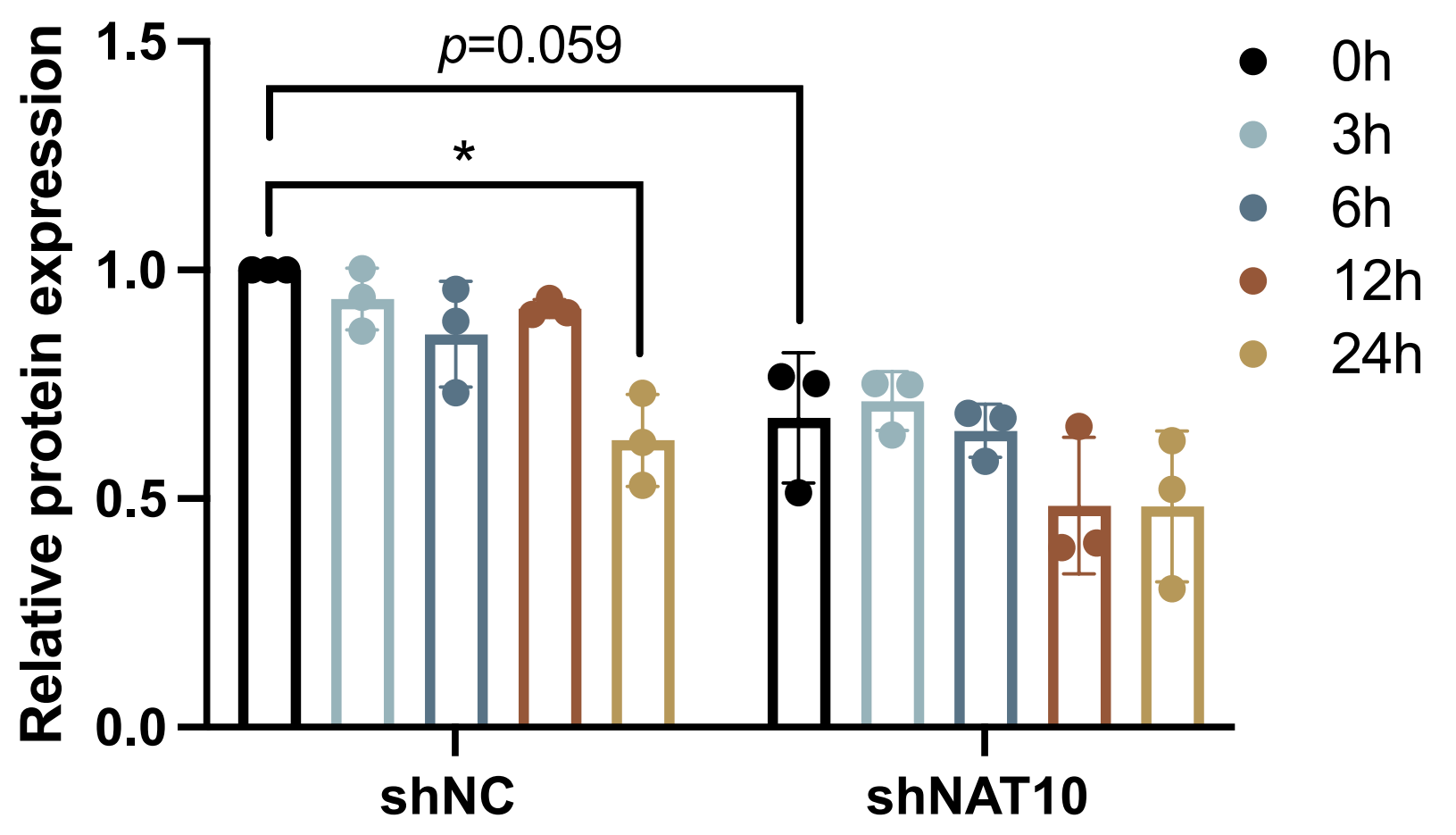

# Figure 6A – FRO

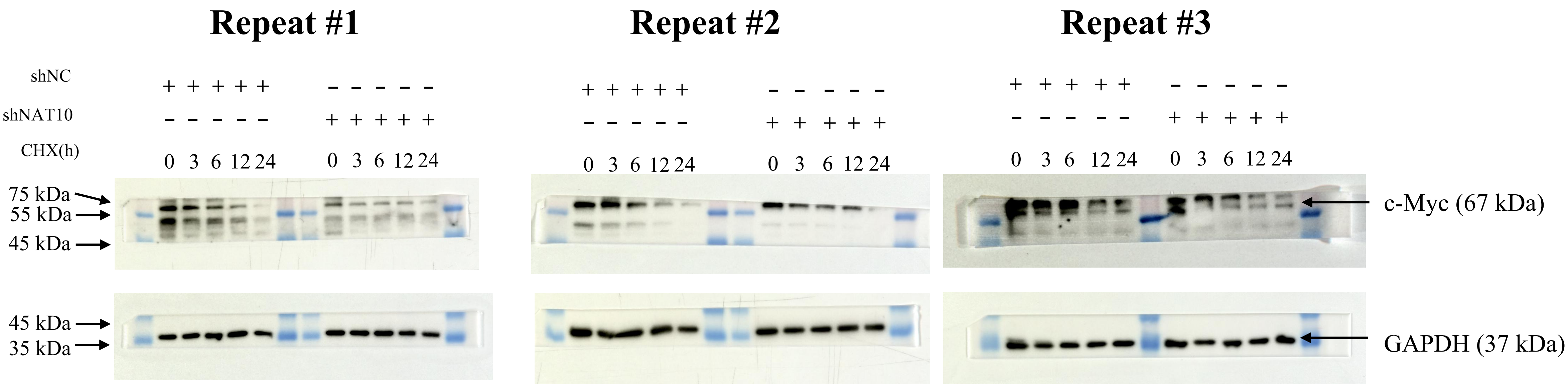


Following transfer, the membrane was cut into strips and probed separately with anti-GAPDH and anti-c-Myc antibodies.

# Figure 6A – FRO

**Western Blot Band Densitometry Measurement**

| Sample | | Repeat #1 | | Repeat #2 | | Repeat #3 | |
|---|---|---|---|---|---|---|---|
| | | c-Myc | GAPDH | c-Myc | GAPDH | c-Myc | GAPDH |
| shNC | 0h | 27445 | 8073 | 19482 | 16496 | 30381 | 12409 |
| | 3h | 18378 | 8087 | 17980 | 14213 | 20858 | 10415 |
| | 6h | 16384 | 9417 | 10583 | 15420 | 21165 | 10639 |
| | 12h | 10012 | 9714 | 5579 | 14534 | 12753 | 10231 |
| | 24h | 4613 | 7323 | 3004 | 11022 | 11899 | 11349 |
| shNAT10 | 0h | 12577 | 9390 | 11770 | 14966 | 20947 | 14359 |
| | 3h | 7478 | 9387 | 6836 | 12522 | 13165 | 9869 |
| | 6h | 8225 | 9736 | 5627 | 12704 | 12577 | 10909 |
| | 12h | 7962 | 7983 | 5924 | 13049 | 9657 | 10460 |
| | 24h | 7332 | 7419 | 1980 | 12016 | 10259 | 12902 |

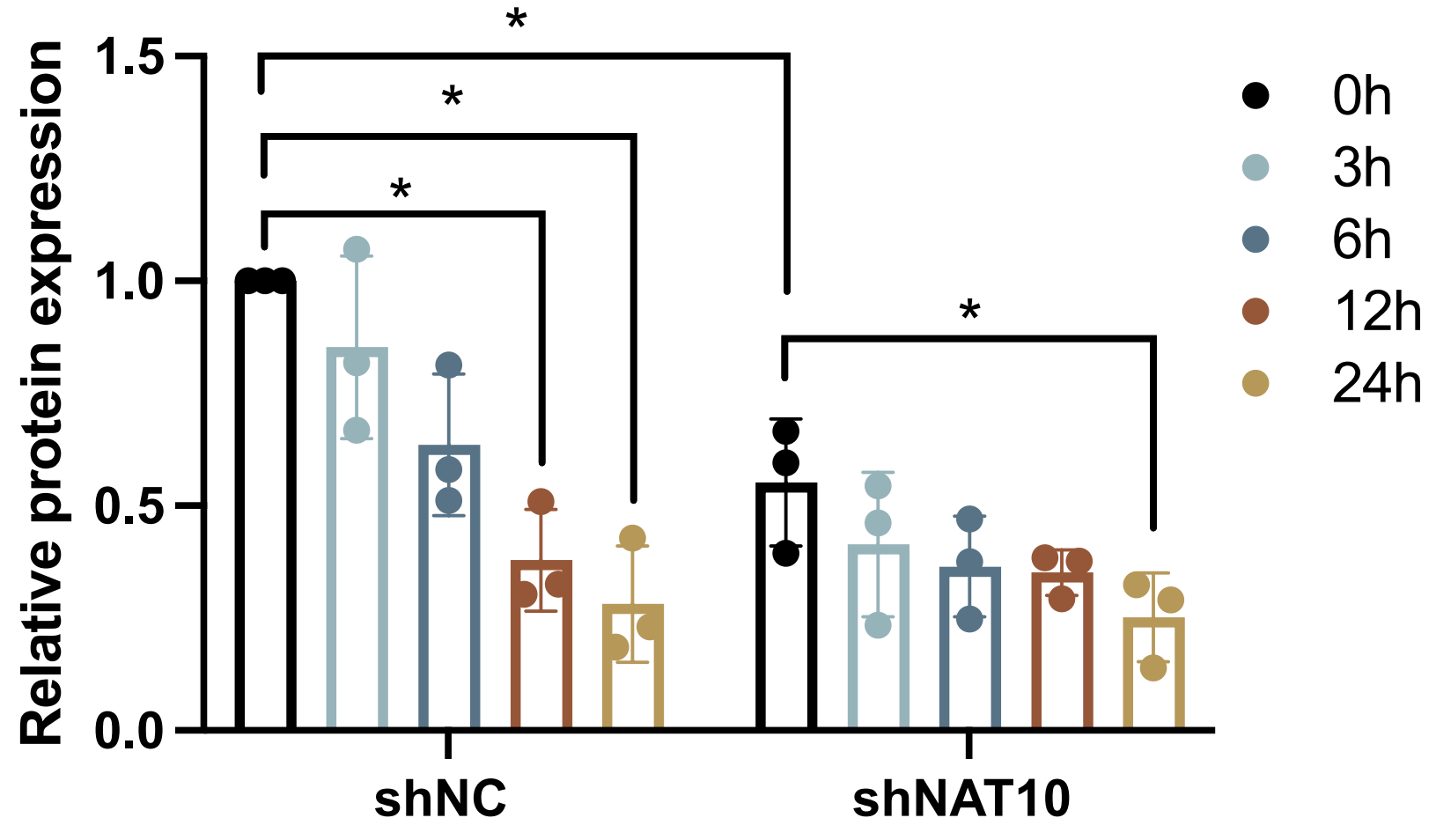

# Figure 6B – 8305C

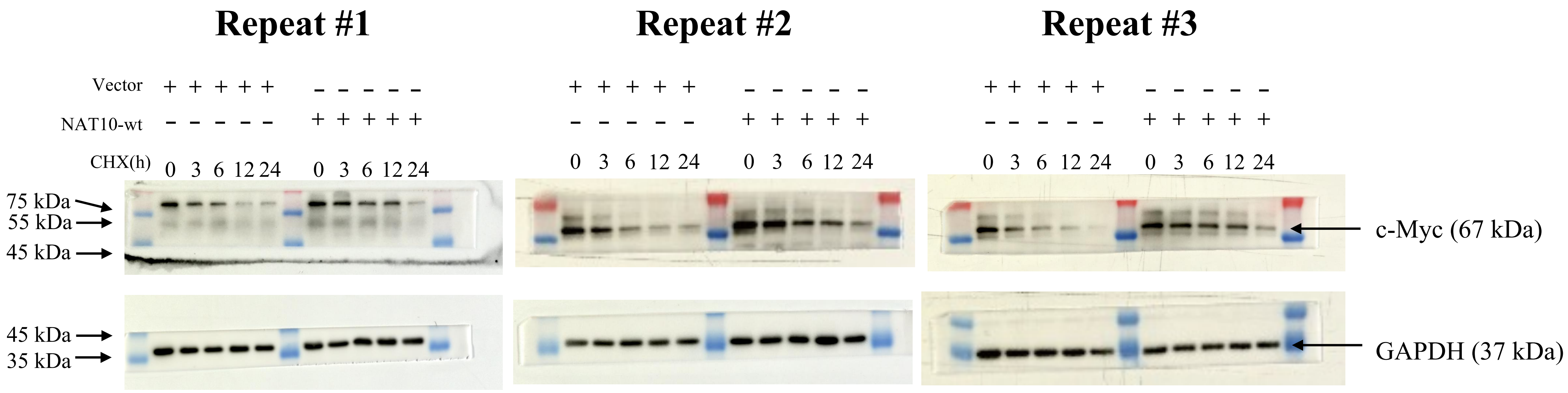


Following transfer, the membrane was cut into strips and probed separately with anti-GAPDH and anti-c-Myc antibodies.

# Figure 6B – 8305C

**Western Blot Band Densitometry Measurement**

| Sample | | Repeat #1 | | Repeat #2 | | Repeat #3 | |
|---|---|---|---|---|---|---|---|
| | | c-Myc | GAPDH | c-Myc | GAPDH | c-Myc | GAPDH |
| shNC | 0h | 13824 | 13360 | 26201 | 9244 | 22063 | 16673 |
| | 3h | 9764 | 10232 | 19890 | 9502 | 13009 | 13890 |
| | 6h | 9211 | 9092 | 13062 | 11178 | 9555 | 13026 |
| | 12h | 7026 | 10420 | 9172 | 9800 | 6602 | 12981 |
| | 24h | 6777 | 9462 | 10093 | 10395 | 4738 | 10073 |
| NAT10-wt | 0h | 18610 | 10870 | 49961 | 12784 | 26028 | 12069 |
| | 3h | 14797 | 9585 | 30825 | 12705 | 21351 | 10340 |
| | 6h | 12313 | 10668 | 21979 | 12929 | 18006 | 10074 |
| | 12h | 12376 | 11062 | 18116 | 16929 | 15384 | 10741 |
| | 24h | 6675 | 10505 | 14304 | 12669 | 8306 | 11079 |

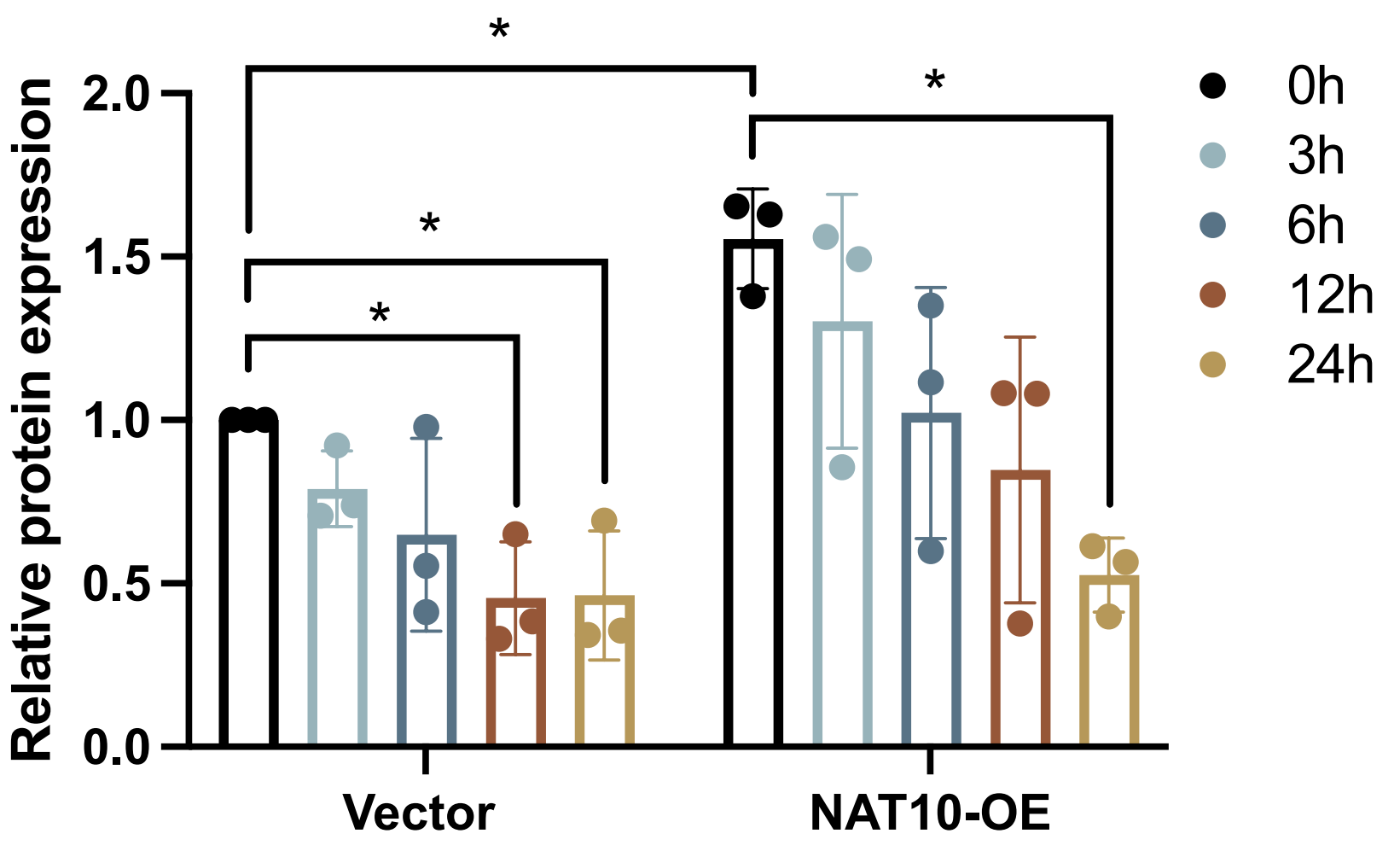

# Figure 6C – 8305C

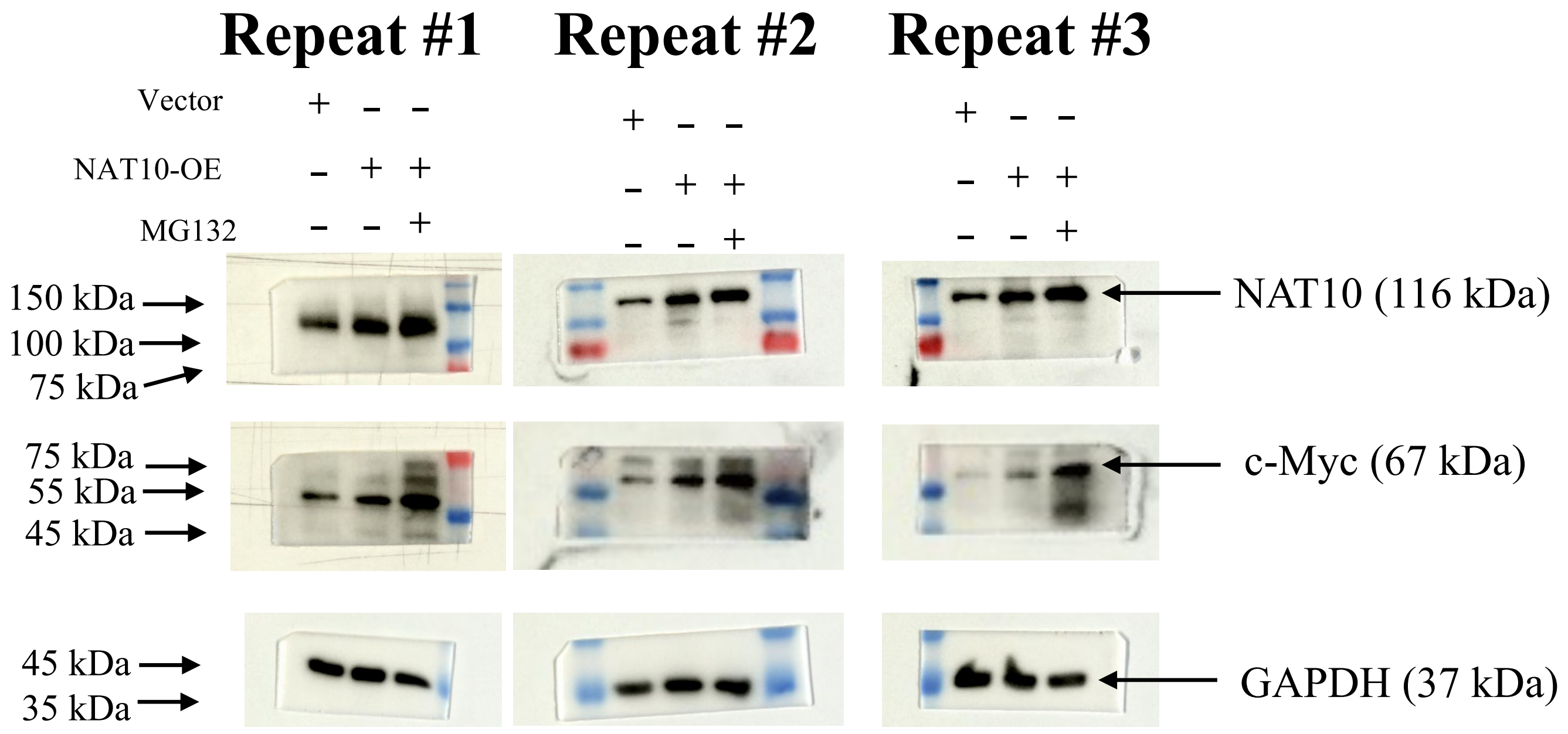


Following transfer, the membrane was cut into strips and probed separately with anti-GAPDH, anti-NAT10 and anti-c-Myc antibodies.

**Western Blot Band Densitometry Measurement**

| Sample | Repeat #1 | | | Repeat #2 | | | Repeat #3 | | |
|---|---|---|---|---|---|---|---|---|---|
| | NAT10 | c-Myc | GAPDH | NAT10 | c-Myc | GAPDH | NAT10 | c-Myc | GAPDH |
| Vector | 17237 | 16859 | 15349 | 6889 | 10492 | 12934 | 9520 | 6530 | 19782 |
| NAT10-OE | 22548 | 25596 | 15496 | 14291 | 20667 | 16740 | 14021 | 10515 | 17918 |
| NAT10-OE + MG132 | 25822 | 43759 | 12625 | 15240 | 32191 | 16323 | 16178 | 27388 | 14719 |

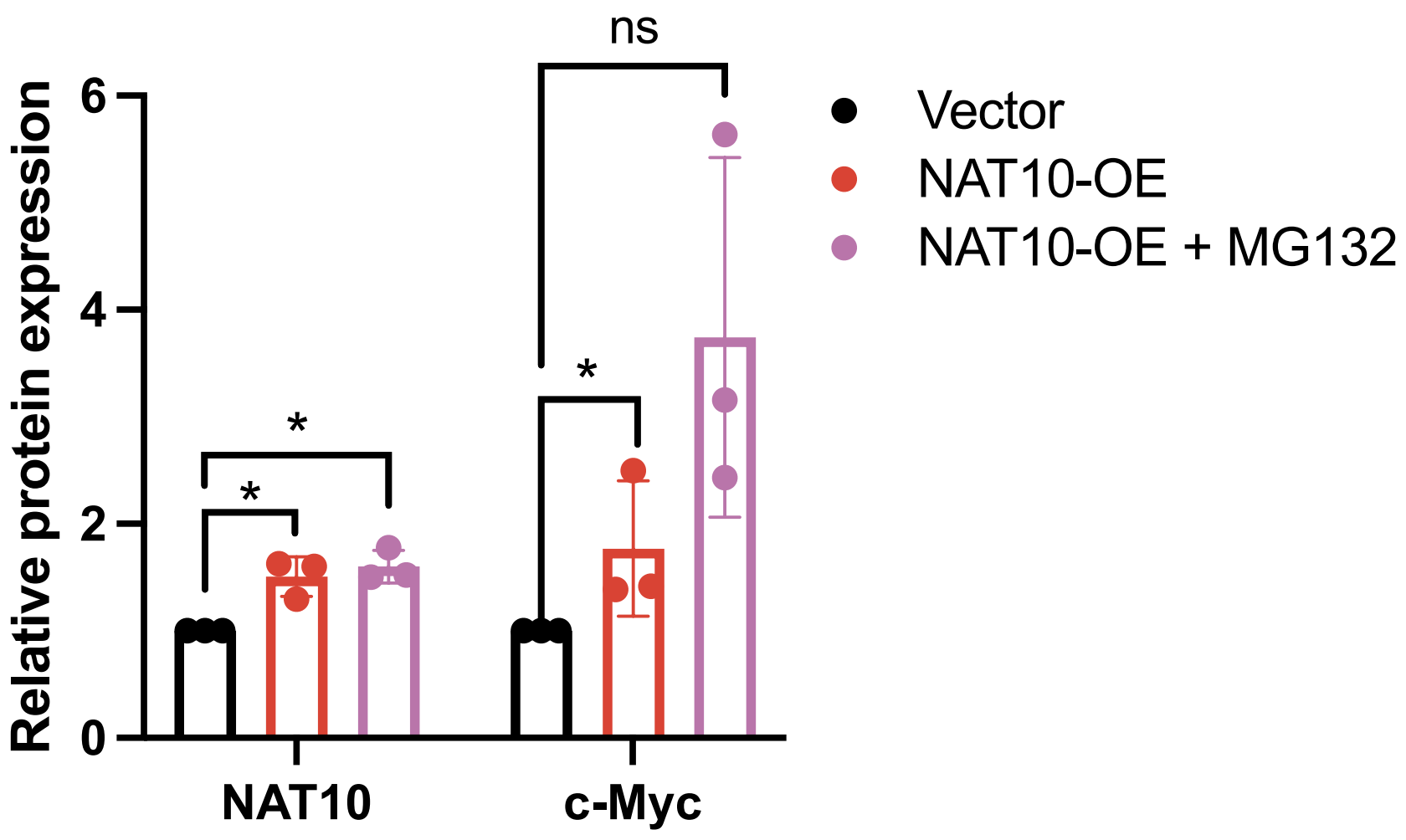

# Figure 6D – KHM-5M

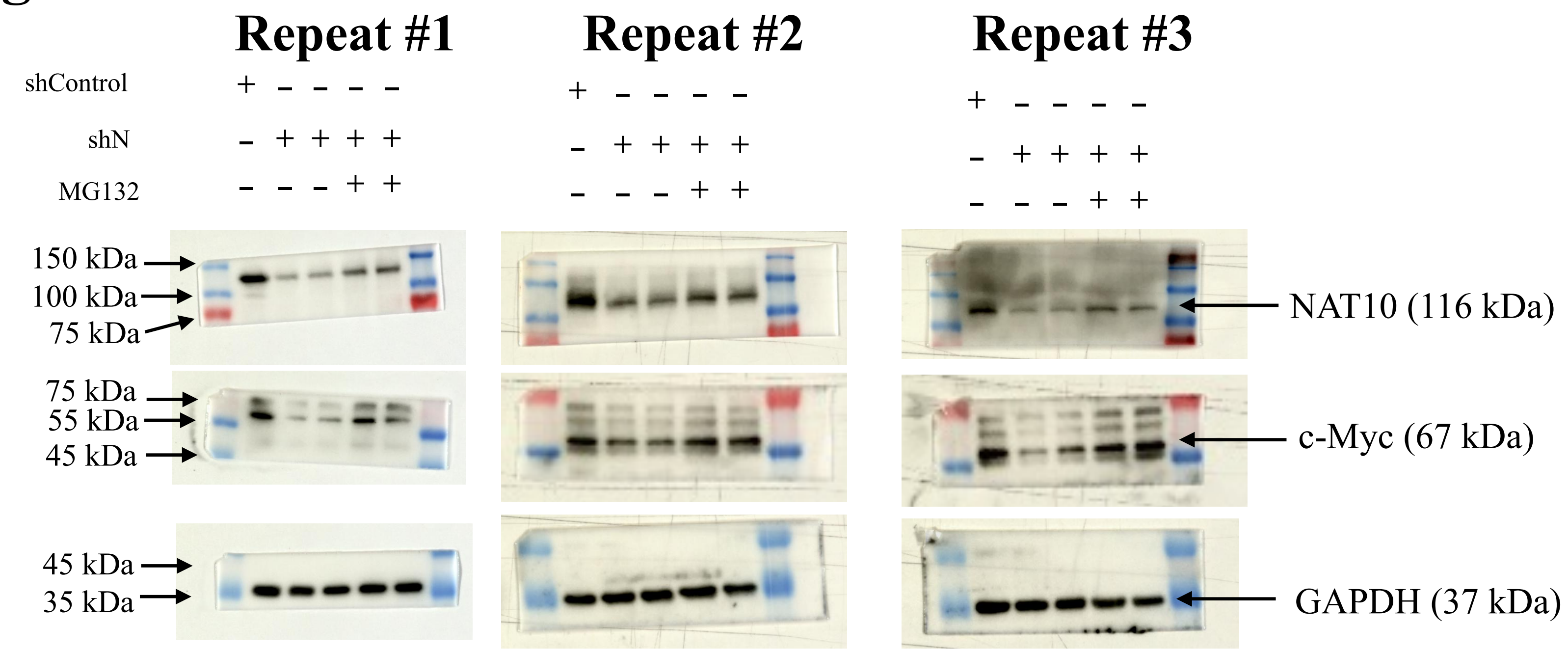


Following transfer, the membrane was cut into strips and probed separately with anti-GAPDH, anti-NAT10 and anti-c-Myc antibodies.

**Western Blot Band Densitometry Measurement**

| Sample | Repeat #1 | | | Repeat #2 | | | Repeat #3 | | |
|---|---|---|---|---|---|---|---|---|---|
| | NAT10 | c-Myc | GAPDH | NAT10 | c-Myc | GAPDH | NAT10 | c-Myc | GAPDH |
| shControl | 10347 | 14408 | 11975 | 24853 | 28013 | 13830 | 14723 | 28001 | 18293 |
| shN-2 | 3331 | 4072 | 10032 | 14274 | 16761 | 15108 | 8599 | 9951 | 15833 |
| shN-4 | 3672 | 5186 | 10512 | 13839 | 14547 | 15438 | 8010 | 14129 | 15067 |
| shN-2 + CHX | 6750 | 11444 | 11794 | 16171 | 24346 | 16323 | 11088 | 27299 | 13680 |
| shN-4 + CHX | 7471 | 10082 | 12511 | 14997 | 23968 | 13382 | 8902 | 31387 | 12554 |

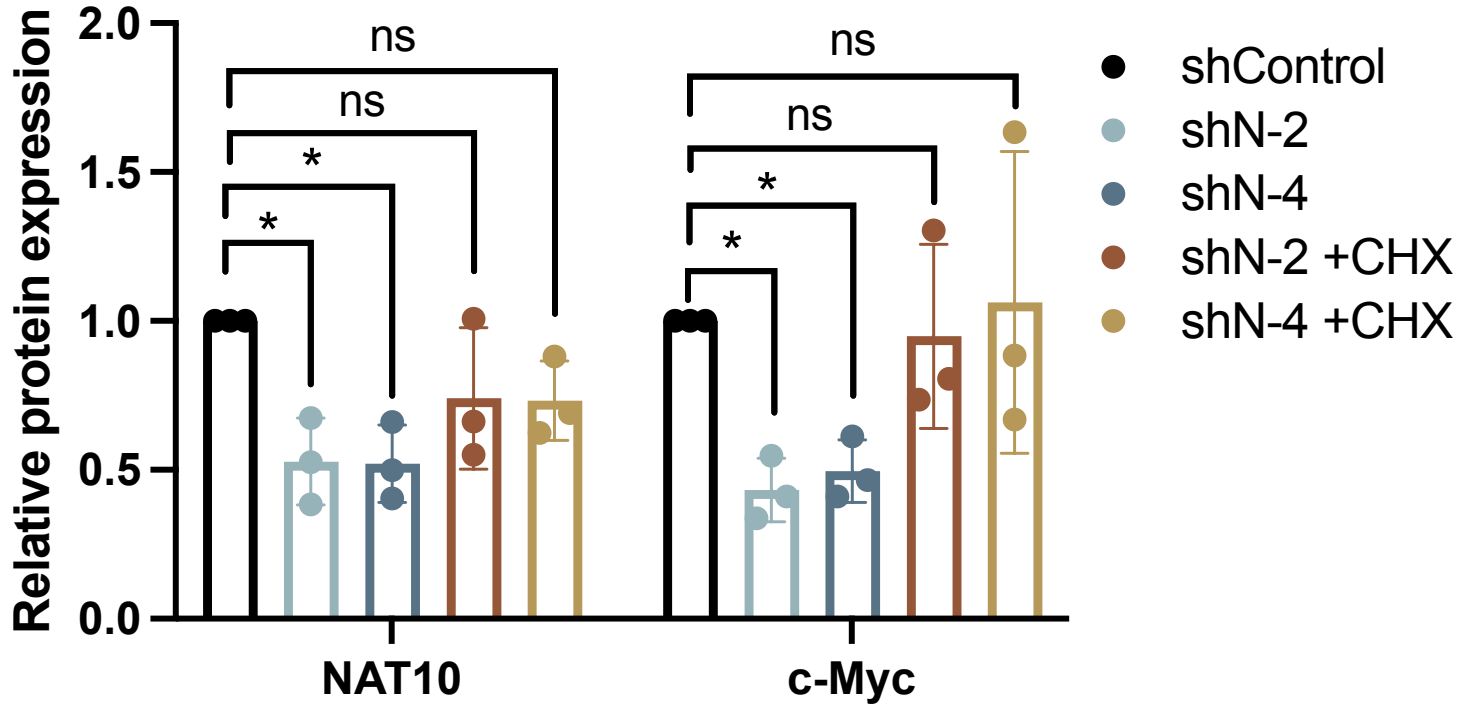

# Figure 6D – FRO

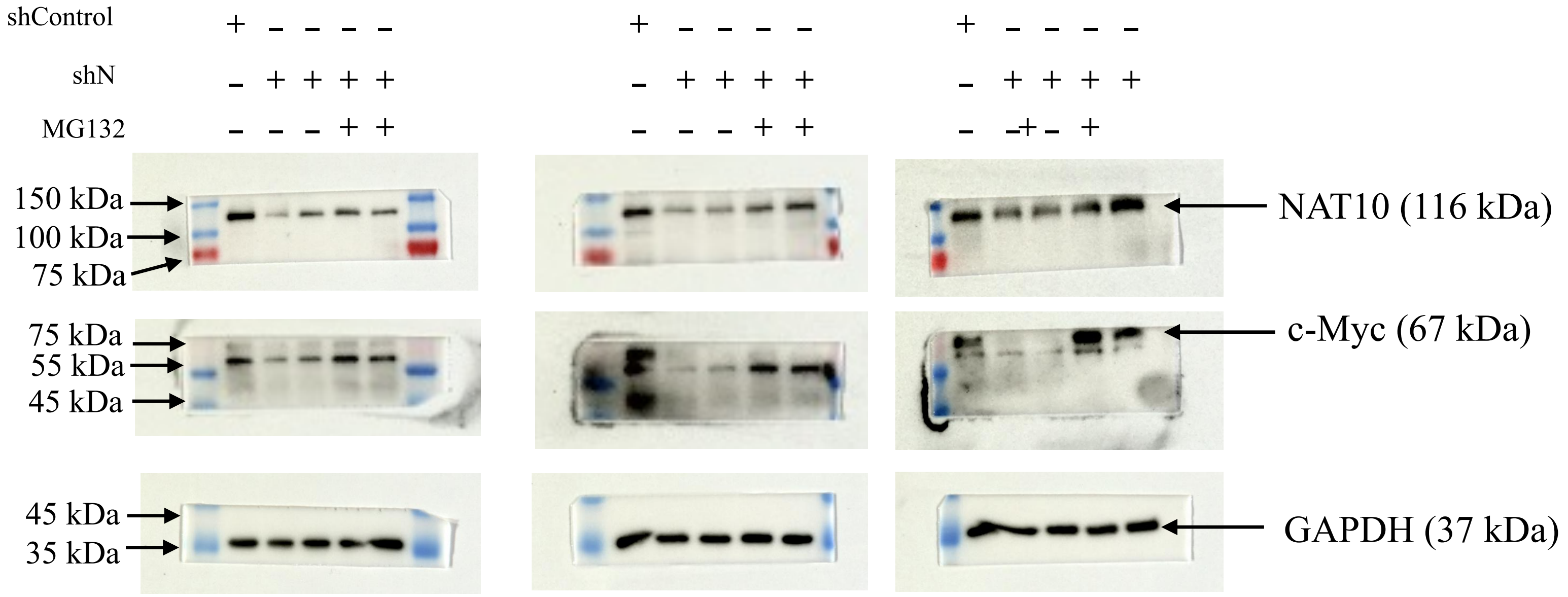


Following transfer, the membrane was cut into strips and probed separately with anti-GAPDH, anti-NAT10 and anti-c-Myc antibodies.

**Western Blot Band Densitometry Measurement**

| Sample | Repeat #1 | | | Repeat #2 | | | Repeat #3 | | |
|---|---|---|---|---|---|---|---|---|---|
| | NAT10 | c-Myc | GAPDH | NAT10 | c-Myc | GAPDH | NAT10 | c-Myc | GAPDH |
| shControl | 8821 | 16214 | 10443 | 10122 | 23863 | 14418 | 13305 | 17709 | 13424 |
| shN-2 | 2350 | 8662 | 8952 | 4931 | 10815 | 11767 | 6482 | 6852 | 10420 |
| shN-3 | 3932 | 10269 | 10914 | 4992 | 8941 | 11291 | 6461 | 5843 | 11079 |
| shN-2 + CHX | 4805 | 15938 | 10124 | 6058 | 19844 | 11444 | 9414 | 20720 | 10677 |
| shN-3 + CHX | 4091 | 15185 | 12905 | 8555 | 19954 | 11351 | 14747 | 17933 | 12693 |

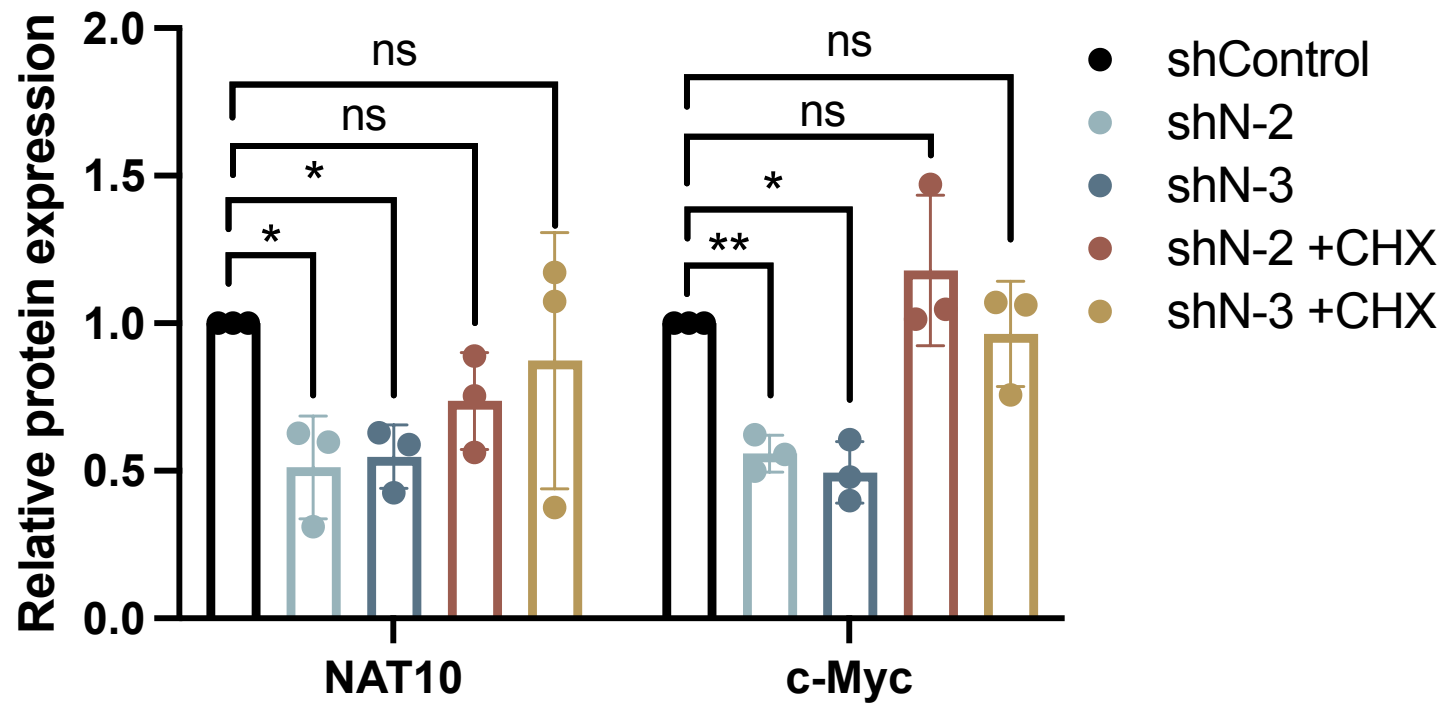

# Figure 6E

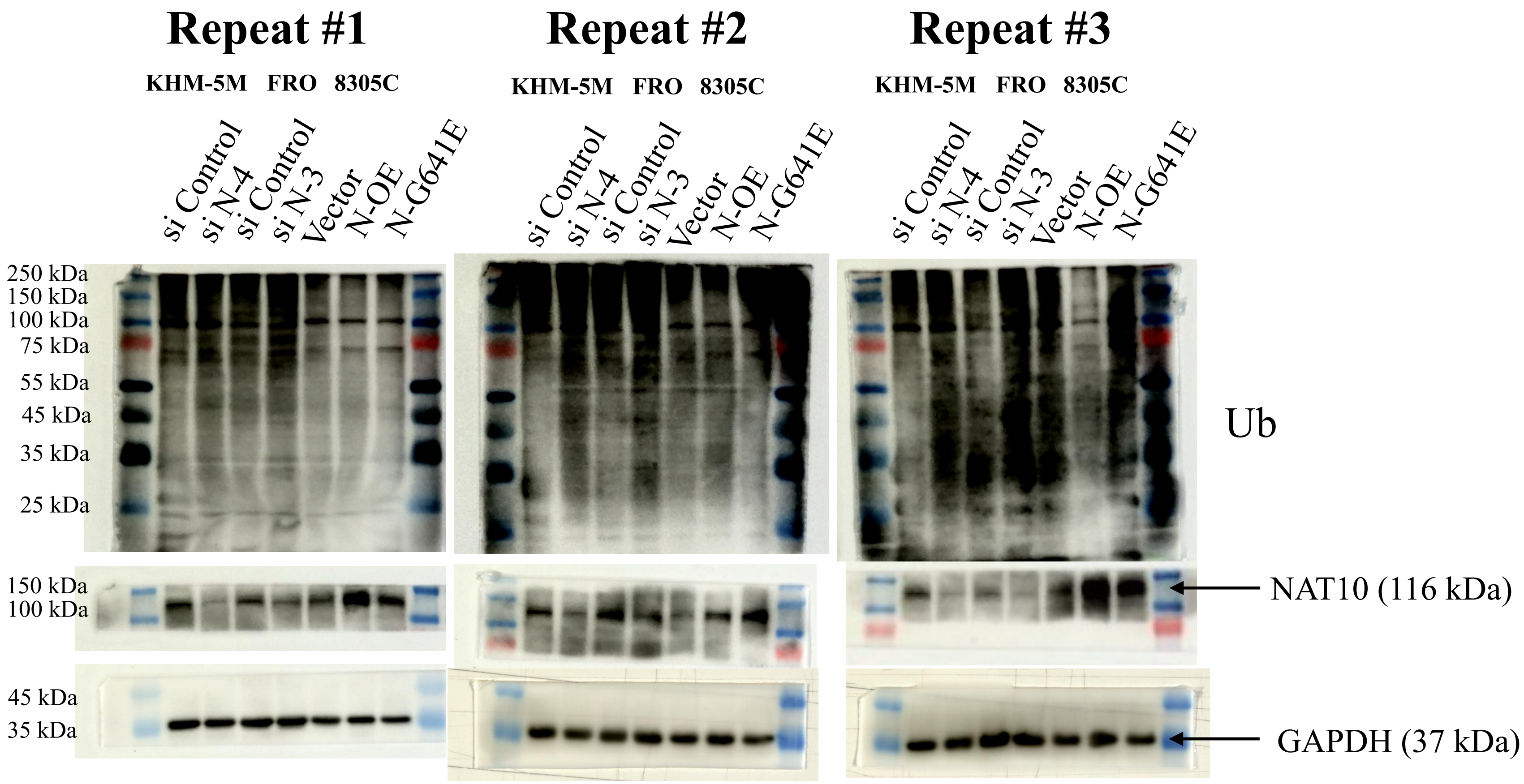


The membrane was entirely transfected and probed with anti-Ub antibody. After detected, the membrane was washed, cut, and subsequently probed with anti-NAT10 and anti-GAPDH antibodies.

**Western Blot Band Densitometry Measurement**

| Sample | | Repeat #1 | | | Repeat #2 | | | Repeat #3 | | |
|---|---|---|---|---|---|---|---|---|---|---|
| | | Ub | NAT10 | GAPDH | Ub | NAT10 | GAPDH | Ub | NAT10 | GAPDH |
| KHM-5M | si Control | 116302 | 28601 | 15951 | 114779 | 32456 | 19102 | 121926 | 20990 | 20645 |
| | si N-4 | 129444 | 20253 | 13999 | 162707 | 22587 | 17071 | 187691 | 14361 | 21480 |
| FRO | si Control | 122487 | 26498 | 14255 | 121876 | 39096 | 17991 | 159470 | 18468 | 25328 |
| | si N-3 | 142572 | 23280 | 14509 | 159021 | 36473 | 18790 | 207272 | 14355 | 24807 |
| 8305C | Vector | 74905 | 26310 | 12247 | 111027 | 23864 | 16804 | 189272 | 29648 | 20047 |
| | N-OE | 64174 | 34247 | 12167 | 79414 | 32291 | 17477 | 113946 | 42083 | 21516 |
| | N-G641E | 83813 | 37540 | 10711 | 114163 | 35954 | 15352 | 203320 | 39054 | 18603 |

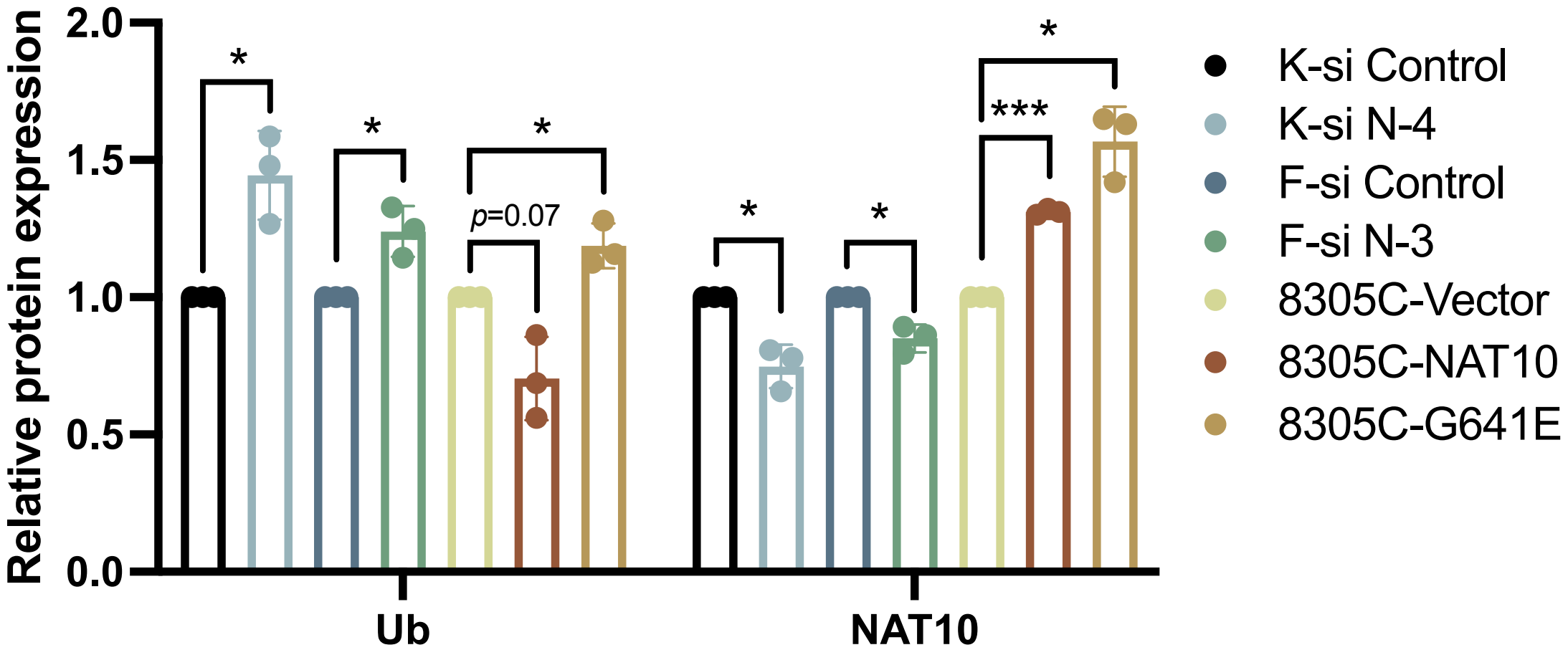

# Figure 6I – KHM-5M

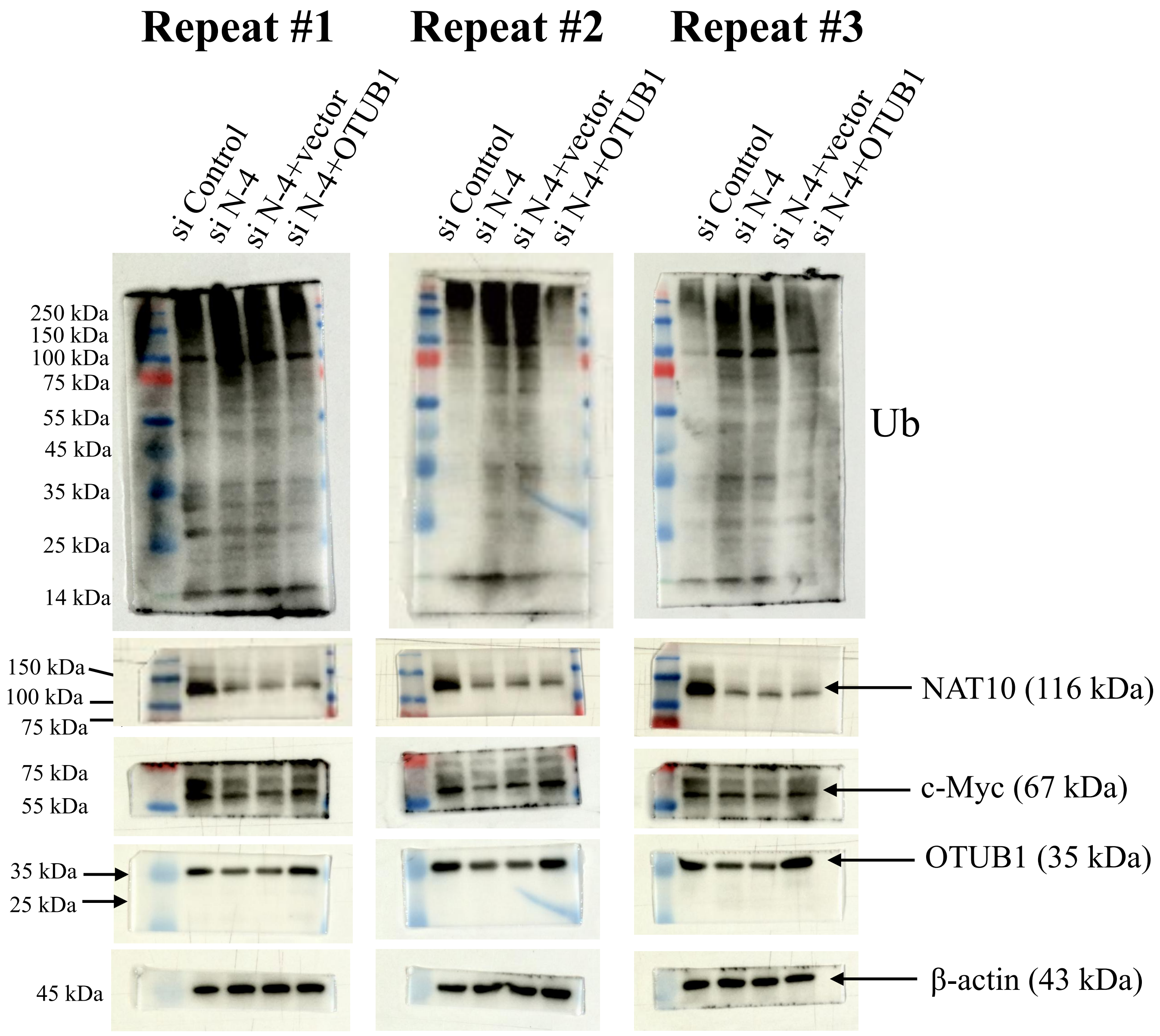


The membrane was entirely transfected and probed with anti-Ub antibody. After detected, the membrane was washed, cut, and subsequently probed with NAT10, c-Myc, OTUB1 and GAPDH antibodies.

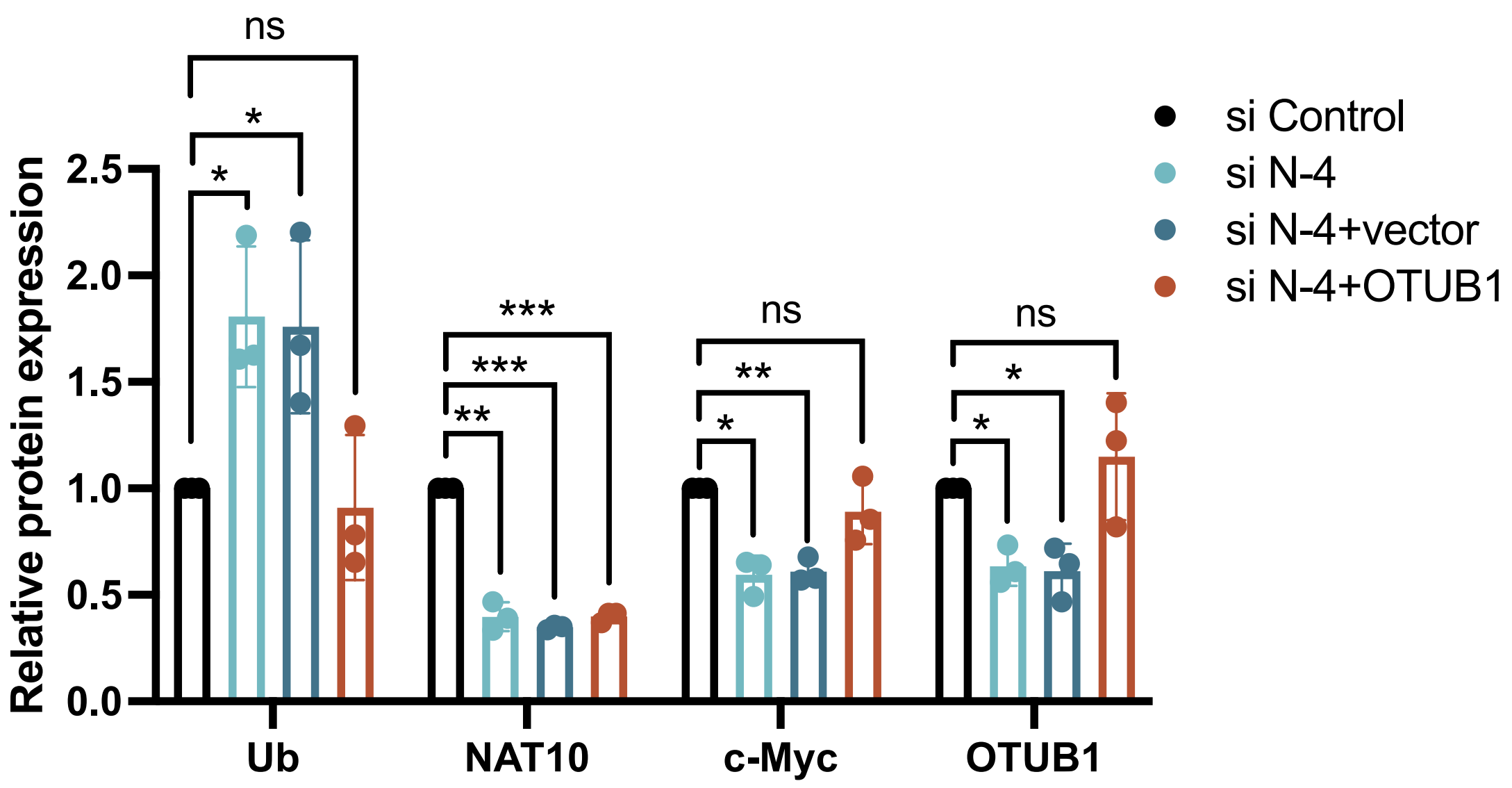

# Figure 6I – FRO

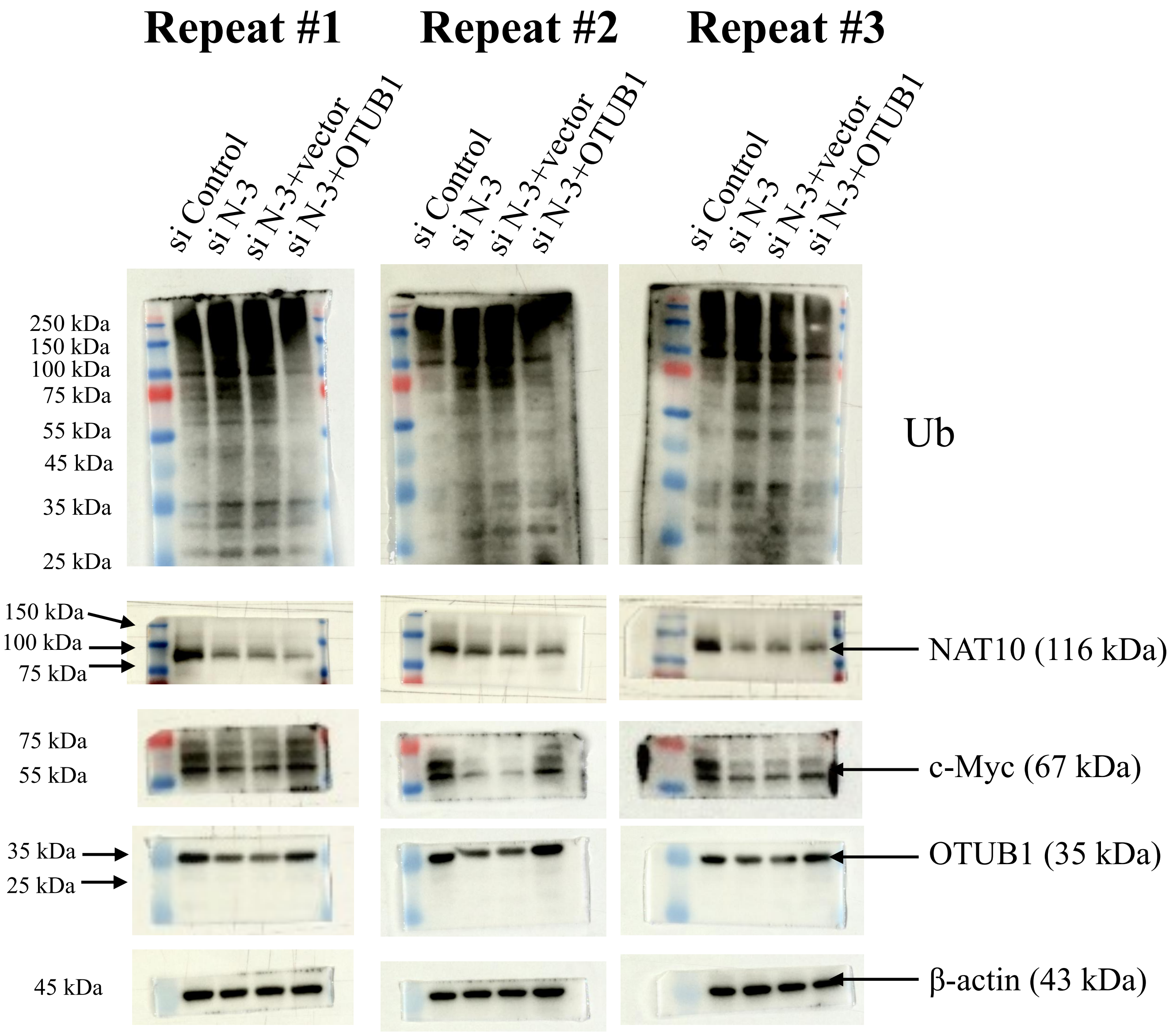


The membrane was entirely transfected and probed with anti-Ub antibody. After detected, the membrane was washed, cut, and subsequently probed with NAT10, c-Myc, OTUB1 and GAPDH antibodies.

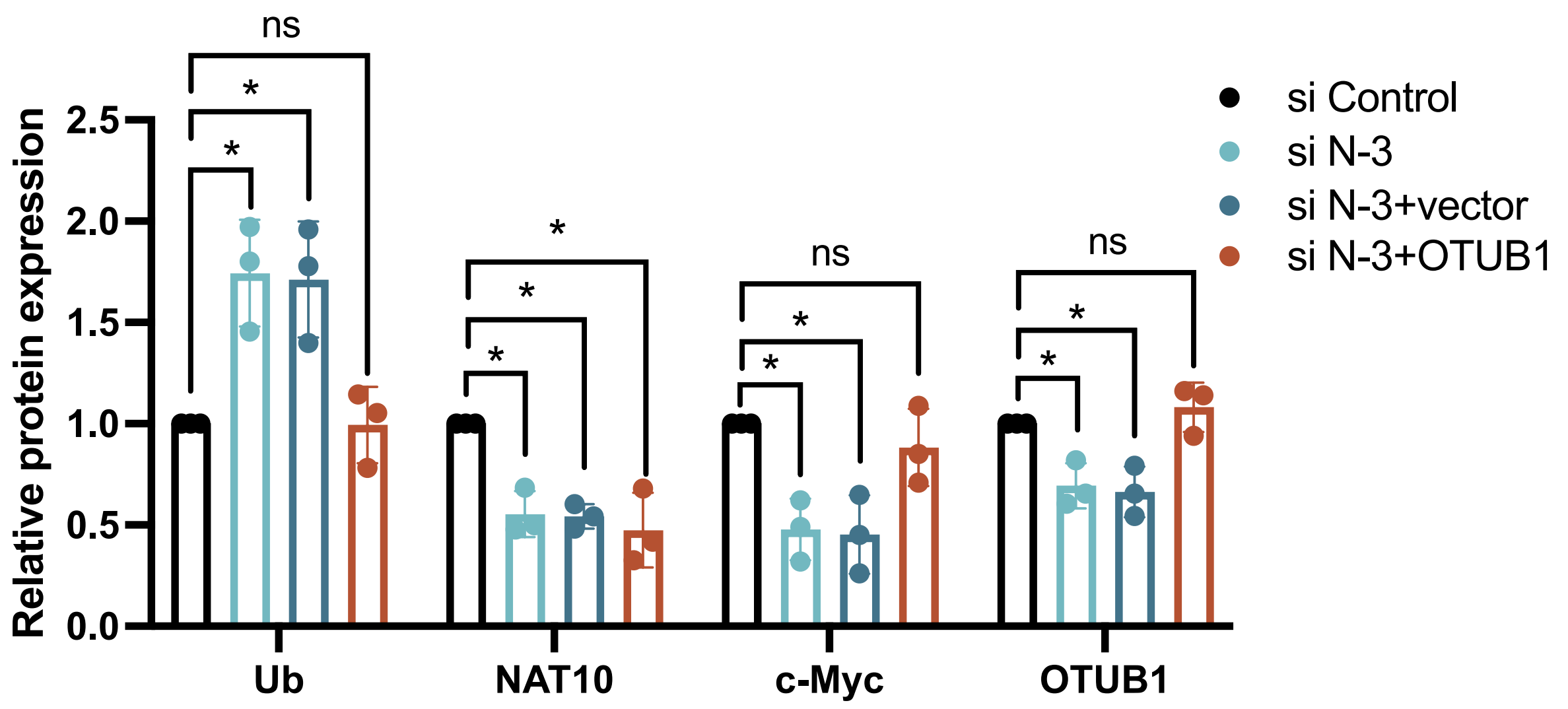

# Figure 6I – KHM-5M

**Western Blot Band Densitometry Measurement**

| Sample | Repeat #1 | | | | | Repeat #2 | | | | | Repeat #3 | | | | |
|---|---|---|---|---|---|---|---|---|---|---|---|---|---|---|---|
| | Ub | NAT10 | c-Myc | OTUB1 | β-actin | Ub | NAT10 | c-Myc | OTUB1 | β-actin | Ub | NAT10 | c-Myc | OTUB1 | β-actin |
| si Control | 98948 | 29577 | 32597 | 9175 | 12832 | 81021 | 22907 | 28963 | 14281 | 12743 | 54292 | 30317 | 34529 | 12761 | 13721 |
| si N-4 | 162744 | 14185 | 21775 | 6895 | 13135 | 157218 | 10653 | 17010 | 9516 | 15205 | 138141 | 11796 | 25745 | 9045 | 15958 |
| si N-4 +vector | 147452 | 11057 | 19728 | 7015 | 13625 | 176208 | 10642 | 21815 | 8697 | 16585 | 121885 | 10392 | 23847 | 8418 | 13984 |
| si N-4 +OTUB1 | 82598 | 13077 | 29766 | 11997 | 13697 | 69635 | 11174 | 28929 | 15429 | 16787 | 64892 | 11539 | 33663 | 16514 | 12658 |

# Figure 6I – FRO

**Western Blot Band Densitometry Measurement**

| Sample | Repeat #1 | | | | | Repeat #2 | | | | | Repeat #3 | | | | |
|---|---|---|---|---|---|---|---|---|---|---|---|---|---|---|---|
| | Ub | NAT10 | c-Myc | OTUB1 | β-actin | Ub | NAT10 | c-Myc | OTUB1 | β-actin | Ub | NAT10 | c-Myc | OTUB1 | β-actin |
| si Control | 132594 | 26997 | 42885 | 13898 | 15435 | 89365 | 24701 | 29637 | 13617 | 12199 | 127605 | 20040 | 27035 | 10024 | 13457 |
| si N-3 | 210948 | 11887 | 23565 | 8420 | 13634 | 179845 | 17270 | 9462 | 8931 | 12447 | 185745 | 10697 | 13281 | 8217 | 15025 |
| si N-3 +vector | 203245 | 11230 | 23987 | 7566 | 13304 | 184426 | 14129 | 7745 | 8918 | 12844 | 178487 | 11866 | 12189 | 7948 | 13219 |
| si N-3 +OTUB1 | 133327 | 8390 | 44491 | 13081 | 14723 | 119988 | 12077 | 25245 | 15532 | 14299 | 99741 | 13150 | 19180 | 11650 | 12975 |

# Figure S1E– 8305C

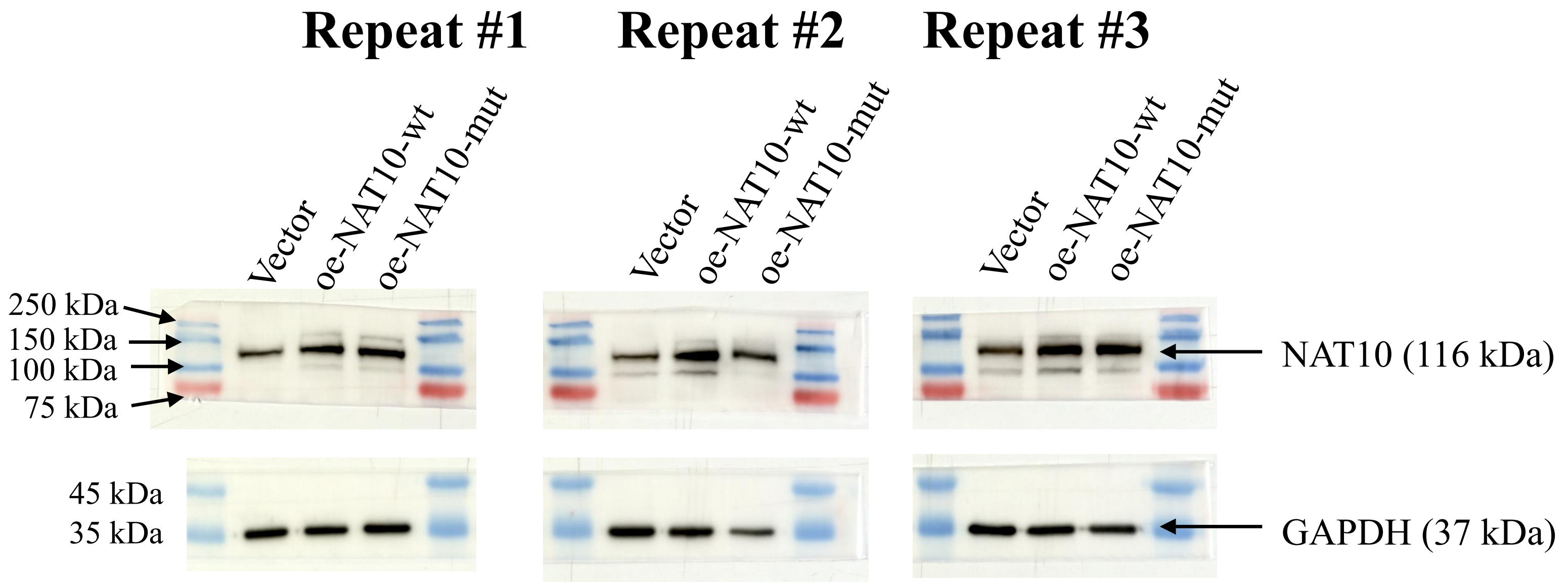


Following transfer, the membrane was cut into strips and probed separately with anti-GAPDH and anti-NAT10 antibodies.

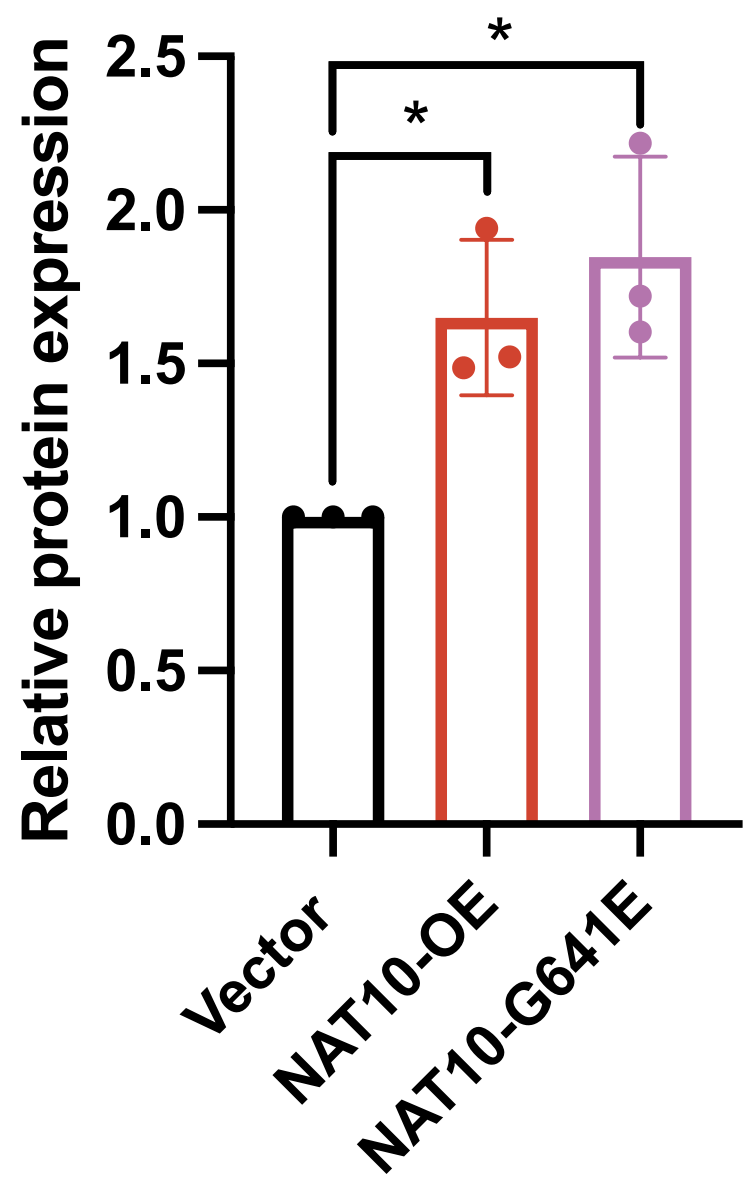


**Western Blot Band Densitometry Measurement**

| Sample | Repeat #1 | | Repeat #2 | | Repeat #3 | |
|---|---|---|---|---|---|---|
| | NAT10 | GAPDH | NAT10 | GAPDH | NAT10 | GAPDH |
| Vector | 11200 | 16988 | 12058 | 20268 | 16089 | 20135 |
| NAT10-OE | 16169 | 16504 | 20357 | 17631 | 22424 | 18452 |
| NAT10-G641E | 20138 | 19053 | 14745 | 11177 | 22075 | 16069 |

# Figure S3B

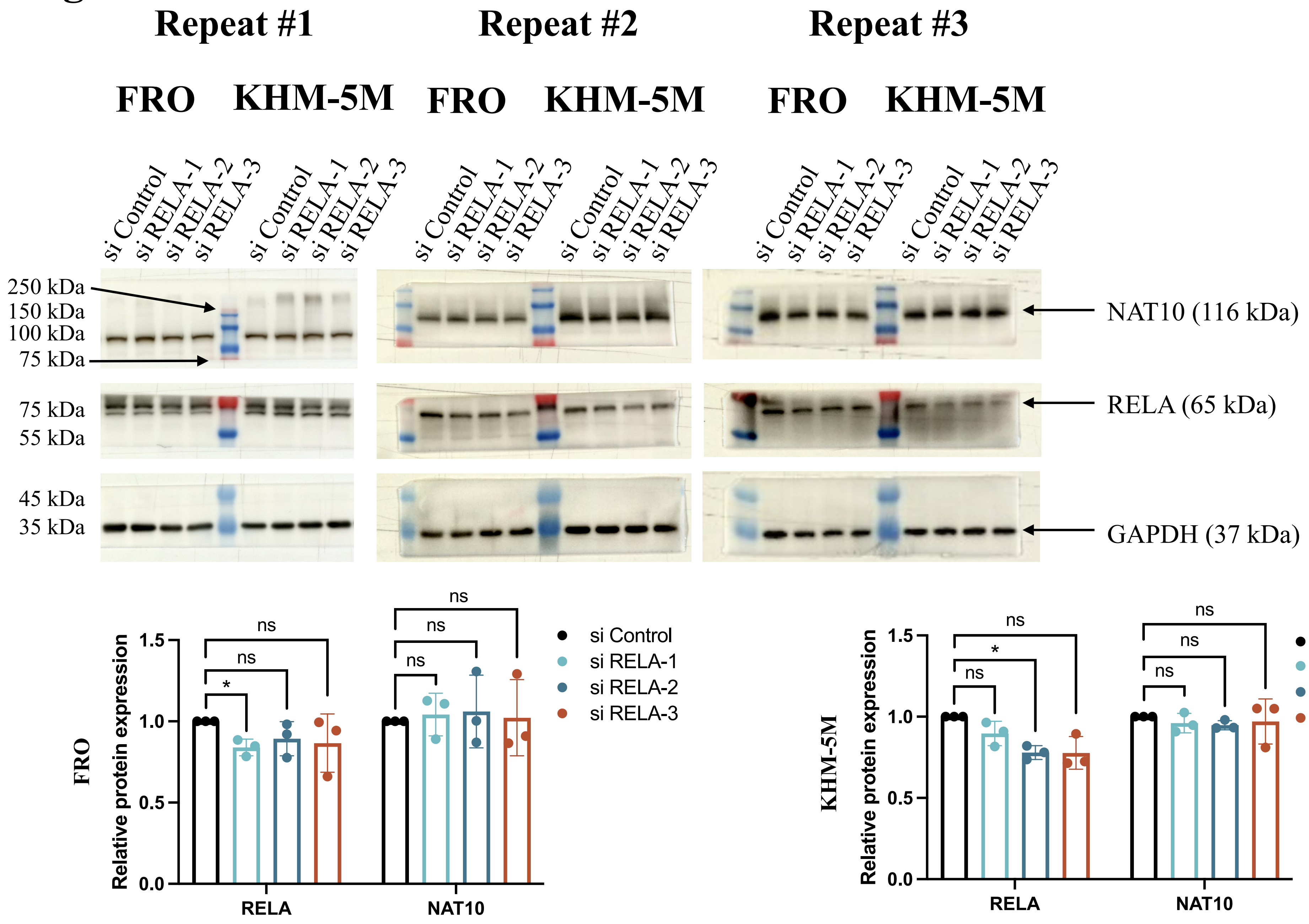


Following transfer, the membrane was cut into strips and probed separately with anti-GAPDH, anti-RELA and anti-NAT10 antibodies.

# Figure S3B – FRO

**Western Blot Band Densitometry Measurement**

| Sample | Repeat #1 | | | Repeat #2 | | | Repeat #3 | | |
|---|---|---|---|---|---|---|---|---|---|
| | NAT10 | RELA | GAPDH | NAT10 | RELA | GAPDH | NAT10 | RELA | GAPDH |
| si Control | 7227 | 19352 | 13557 | 12431 | 12000 | 12961 | 20809 | 15166 | 12113 |
| si RELA-1 | 7989 | 15142 | 13525 | 13119 | 9528 | 12133 | 16601 | 12025 | 10851 |
| si RELA-2 | 7118 | 14318 | 10214 | 12926 | 9660 | 13427 | 15961 | 12296 | 10669 |
| si RELA-3 | 7181 | 14793 | 10426 | 12184 | 8546 | 13962 | 14998 | 11916 | 10090 |

# Figure S3B – KHM-5M

**Western Blot Band Densitometry Measurement**

| Sample | Repeat #1 | | | Repeat #2 | | | Repeat #3 | | |
|---|---|---|---|---|---|---|---|---|---|
| | NAT10 | RELA | GAPDH | NAT10 | RELA | GAPDH | NAT10 | RELA | GAPDH |
| si Control | 7356 | 16655 | 10108 | 19511 | 10341 | 15844 | 17444 | 13354 | 11224 |
| si RELA-1 | 8083 | 17220 | 10833 | 16767 | 8893 | 14993 | 16333 | 10771 | 11082 |
| si RELA-2 | 7920 | 15048 | 11700 | 16700 | 6987 | 14537 | 17112 | 10973 | 11234 |
| si RELA-3 | 7111 | 14188 | 12048 | 18772 | 8470 | 14520 | 16867 | 8894 | 10327 |

# Figure S3E

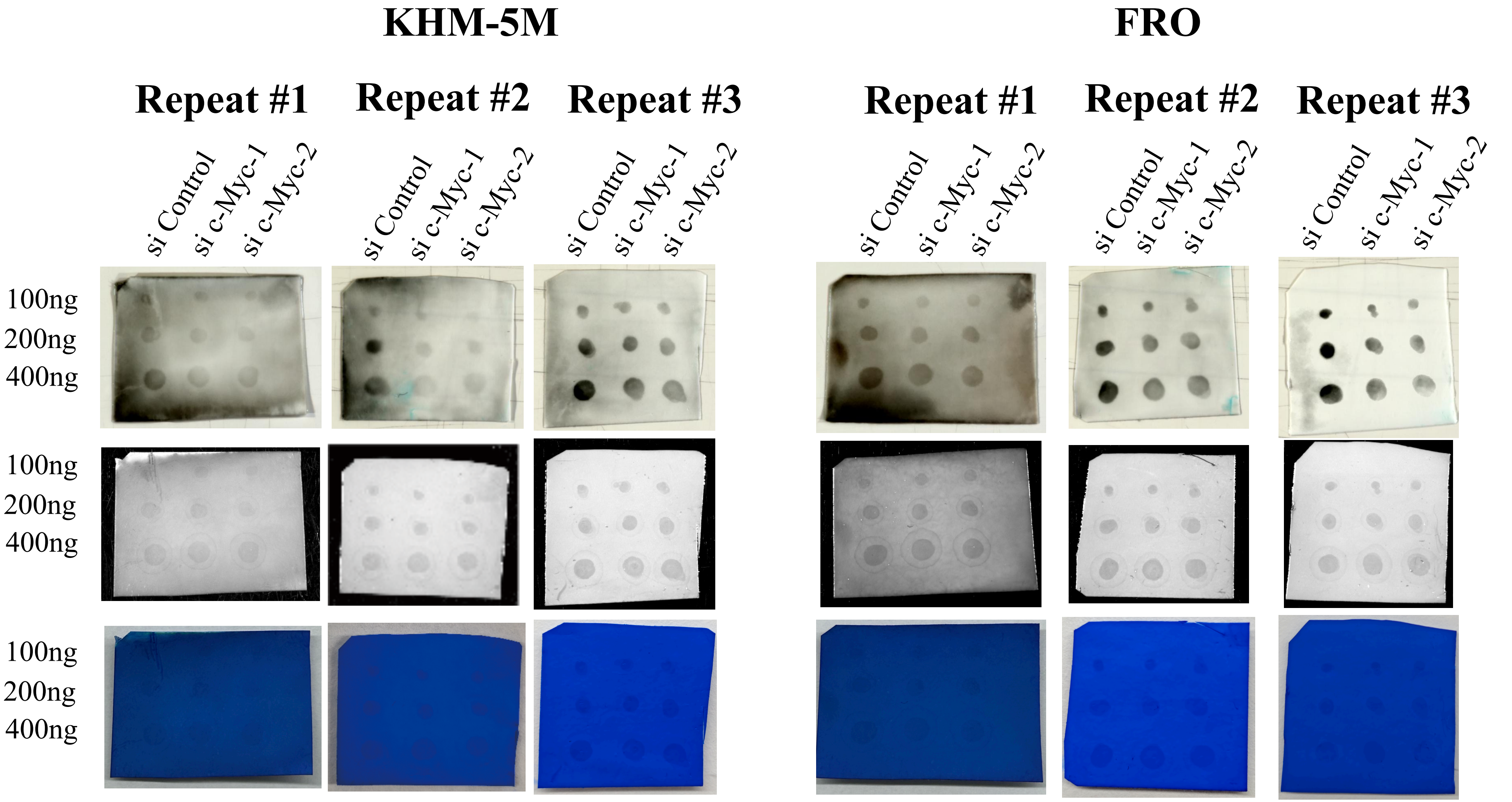


The entire membrane was probed with an antibody against N4-acetylcytidine.

# Figure S3E

## KHM-5M

**Dot Blot Band Densitometry Measurement**

| Sample | Repeat #1 | Repeat #2 | Repeat #3 |
|---|---|---|---|
| | $ac^4c$ | $ac^4c$ | $ac^4c$ |
| si Control | 25132 | 27581 | 27961 |
| si c-Myc-1 | 11232 | 7038 | 14240 |
| si c-Myc-2 | 12228 | 5347 | 10049 |

## FRO

**Dot Blot Band Densitometry Measurement**

| Sample | Repeat #1 | Repeat #2 | Repeat #3 |
|---|---|---|---|
| | $ac^4c$ | $ac^4c$ | $ac^4c$ |
| si Control | 36807 | 19504 | 17223 |
| si c-Myc-1 | 24701 | 9987 | 7911 |
| si c-Myc-2 | 21263 | 10984 | 5692 |

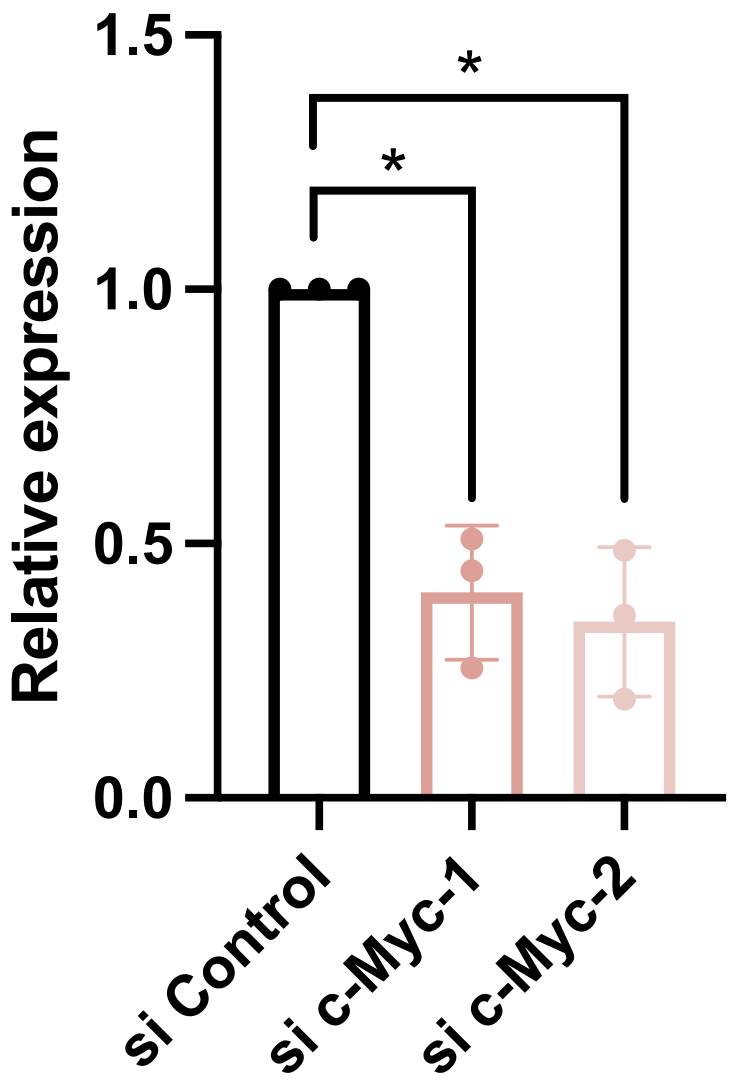


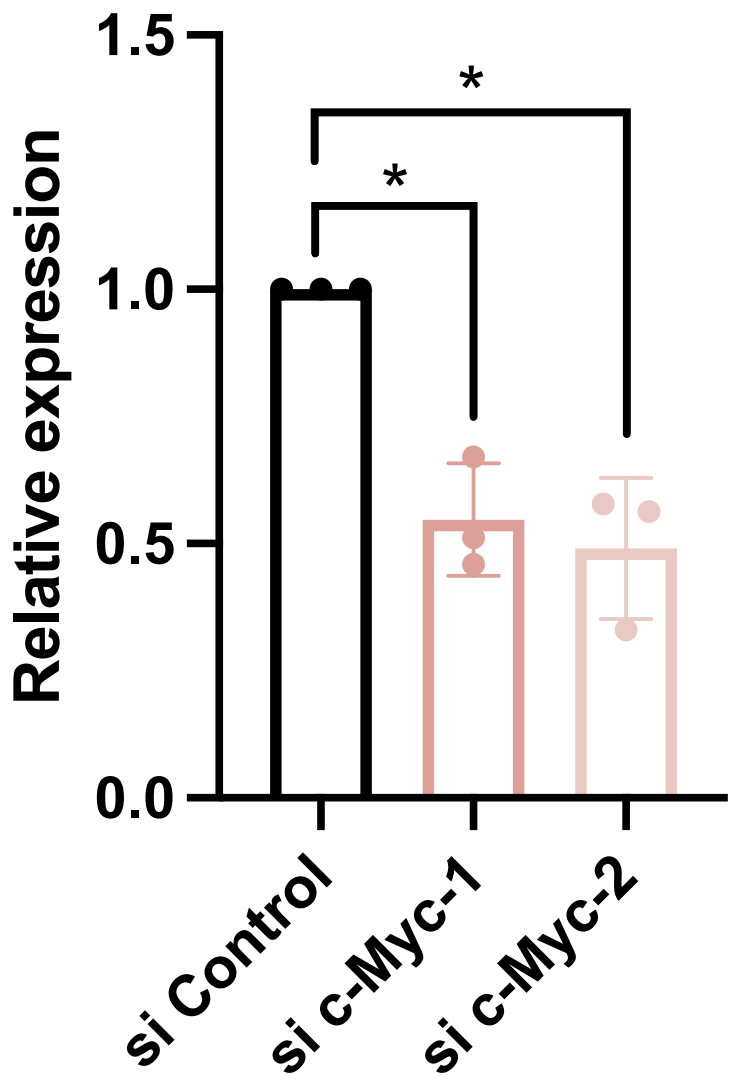

# Figure S4E KHM-5M

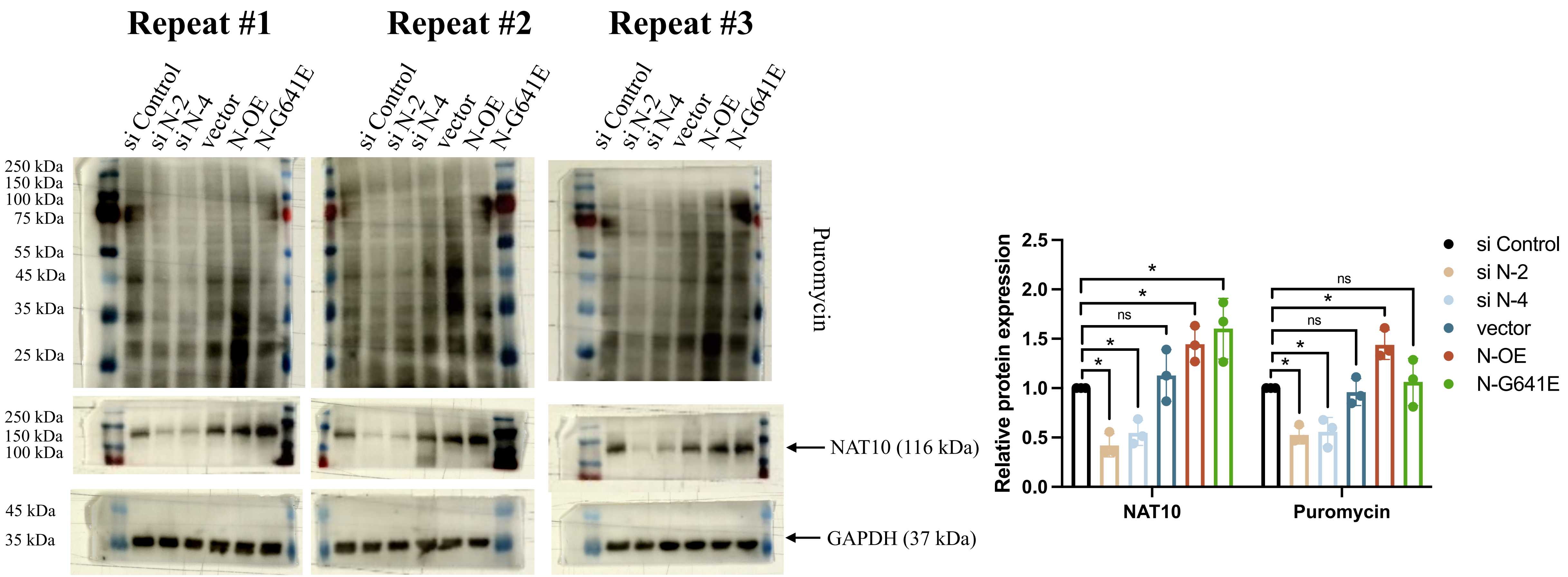

**Western Blot Band Densitometry Measurement**

| Sample | Repeat #1 | | | Repeat #2 | | | Repeat #3 | | |
|---|---|---|---|---|---|---|---|---|---|
| | Puromycin | NAT10 | GAPDH | Puromycin | NAT10 | GAPDH | Puromycin | NAT10 | GAPDH |
| si Control | 130151 | 10415 | 24631 | 116802 | 13079 | 20948 | 71643 | 16268 | 19233 |
| si N-2 | 57946 | 5391 | 22887 | 67303 | 4283 | 19181 | 31772 | 5359 | 18242 |
| si N-4 | 42663 | 5899 | 20294 | 77939 | 5652 | 20629 | 46372 | 9031 | 20773 |
| vector | 97598 | 12825 | 21799 | 109535 | 14989 | 21322 | 88003 | 15590 | 21263 |
| N-OE | 158009 | 15519 | 22494 | 160241 | 18651 | 20841 | 122078 | 21871 | 20370 |
| N-G641E | 104800 | 19291 | 24427 | 122457 | 21057 | 20144 | 107078 | 23900 | 22335 |

# Figure S4E

## FRO

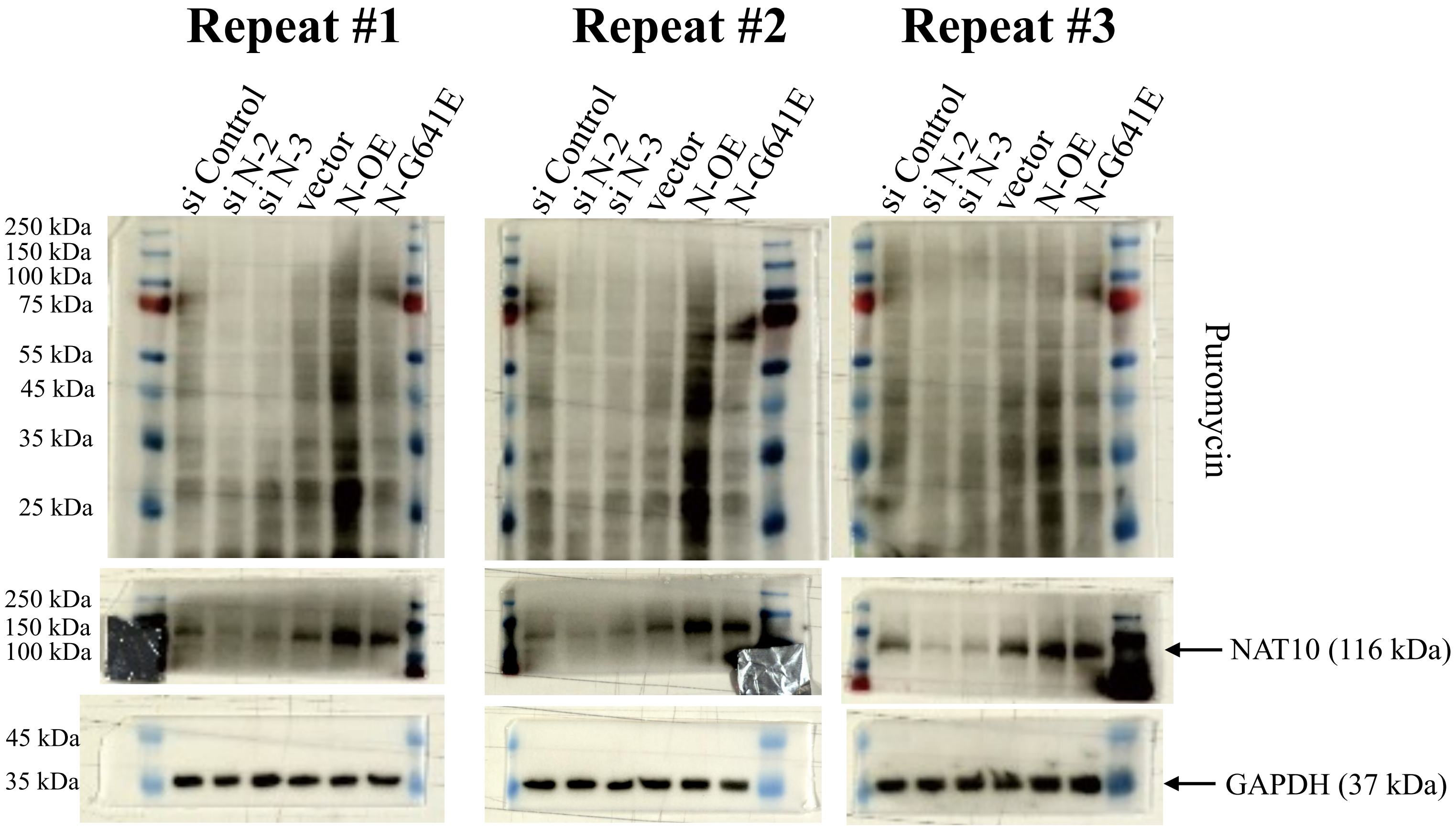


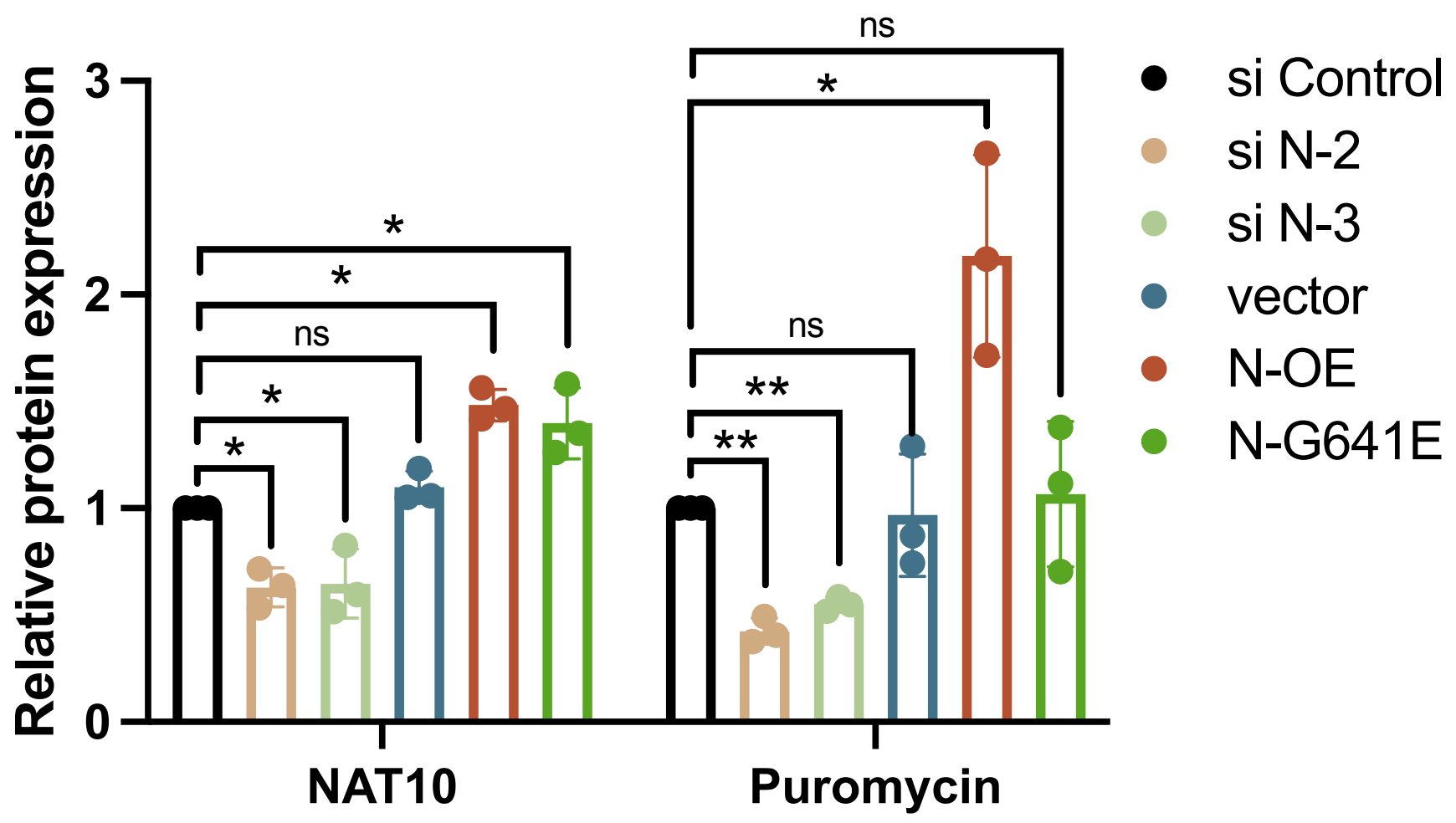

**Western Blot Band Densitometry Measurement**

| Sample | Repeat #1 | | | Repeat #2 | | | Repeat #3 | | |
|---|---|---|---|---|---|---|---|---|---|
| | Puromycin | NAT10 | GAPDH | Puromycin | NAT10 | GAPDH | Puromycin | NAT10 | GAPDH |
| si Control | 71560 | 20820 | 14044 | 96140 | 19314 | 14732 | 83198 | 15207 | 19422 |
| si N-2 | 24481 | 12586 | 11855 | 35356 | 12062 | 14401 | 40985 | 8110 | 19403 |
| si N-3 | 40187 | 11615 | 15168 | 46519 | 13168 | 12145 | 48041 | 9553 | 20423 |
| vector | 88293 | 21091 | 13424 | 68964 | 22113 | 14222 | 77614 | 17133 | 20815 |
| N-OE | 170201 | 27276 | 12547 | 180509 | 26189 | 12774 | 163452 | 24730 | 22248 |
| N-G641E | 94158 | 25046 | 13397 | 77513 | 22067 | 10640 | 72155 | 25320 | 23941 |

# Figure S5C

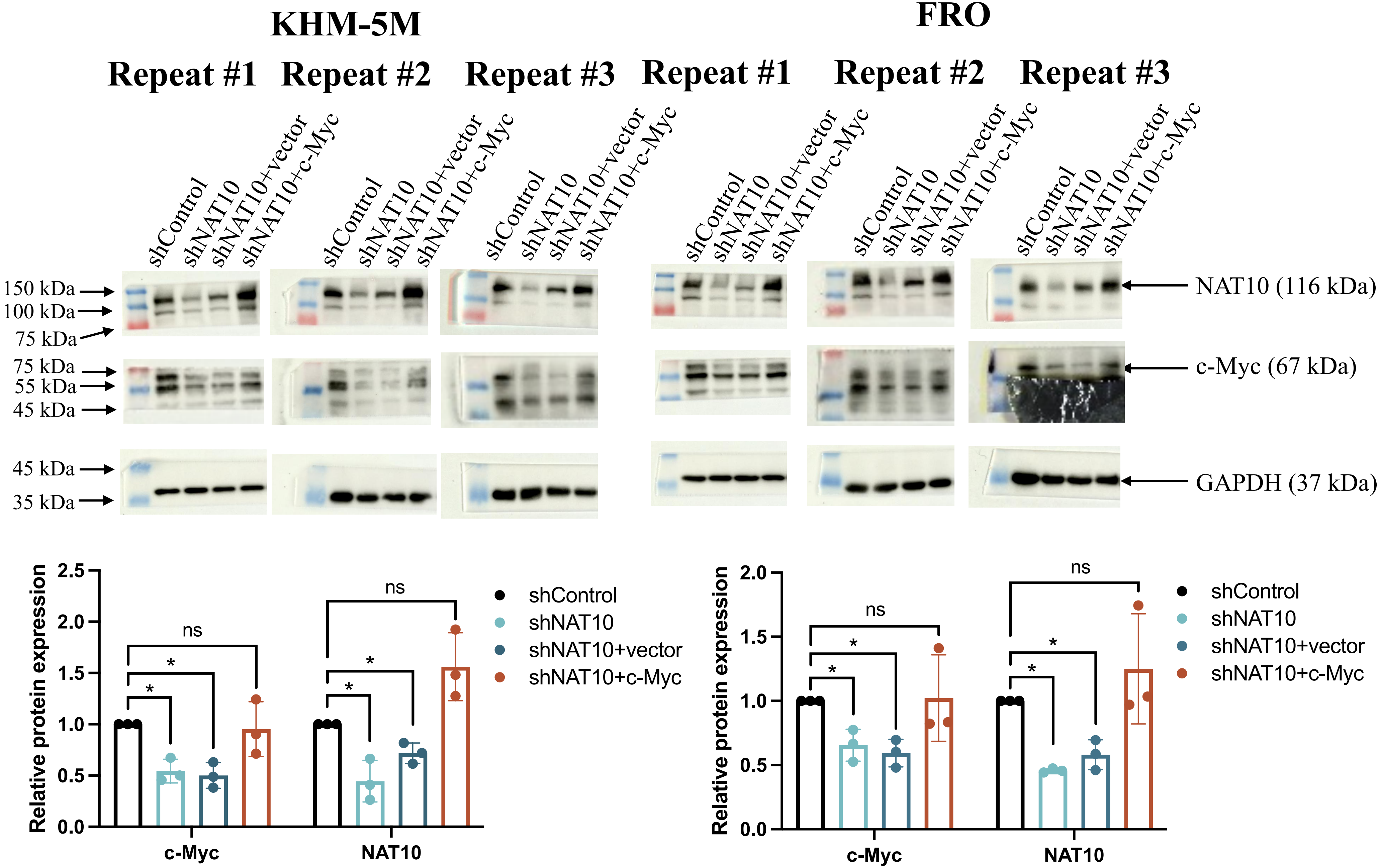


Following transfer, the membrane was cut into strips and probed separately with anti-GAPDH, anti-c-Myc and anti-NAT10 antibodies.

# Figure S5C – KHM-5M

**Western Blot Band Densitometry Measurement**

| Sample | Repeat #1 | | | Repeat #2 | | | Repeat #3 | | |
|---|---|---|---|---|---|---|---|---|---|
| | NAT10 | c-Myc | GAPDH | NAT10 | c-Myc | GAPDH | NAT10 | c-Myc | GAPDH |
| shControl | 11445 | 27212 | 11690 | 19420 | 19386 | 20158 | 16602 | 37216 | 19991 |
| shNAT10 | 4990 | 13269 | 12482 | 10260 | 7717 | 16041 | 4280 | 24560 | 19556 |
| shNAT10+vector | 7480 | 12053 | 10610 | 12353 | 5760 | 15693 | 8221 | 18855 | 16056 |
| shNAT10+c-Myc | 14757 | 19590 | 11807 | 29017 | 13581 | 15654 | 16782 | 31510 | 13629 |

# Figure S5C – FRO

**Western Blot Band Densitometry Measurement**

| Sample | Repeat #1 | | | Repeat #2 | | | Repeat #3 | | |
|---|---|---|---|---|---|---|---|---|---|
| | NAT10 | c-Myc | GAPDH | NAT10 | c-Myc | GAPDH | NAT10 | c-Myc | GAPDH |
| shControl | 20366 | 21705 | 11695 | 24067 | 37770 | 15354 | 13555 | 13661 | 29505 |
| shNAT10 | 9974 | 12668 | 12967 | 11557 | 25494 | 15569 | 4765 | 8162 | 22782 |
| shNAT10+vector | 11210 | 12470 | 13963 | 15032 | 24210 | 16329 | 6899 | 6978 | 21655 |
| shNAT10+c-Myc | 23894 | 20238 | 13264 | 24786 | 33406 | 16278 | 16885 | 13764 | 21074 |

# Figure S5K

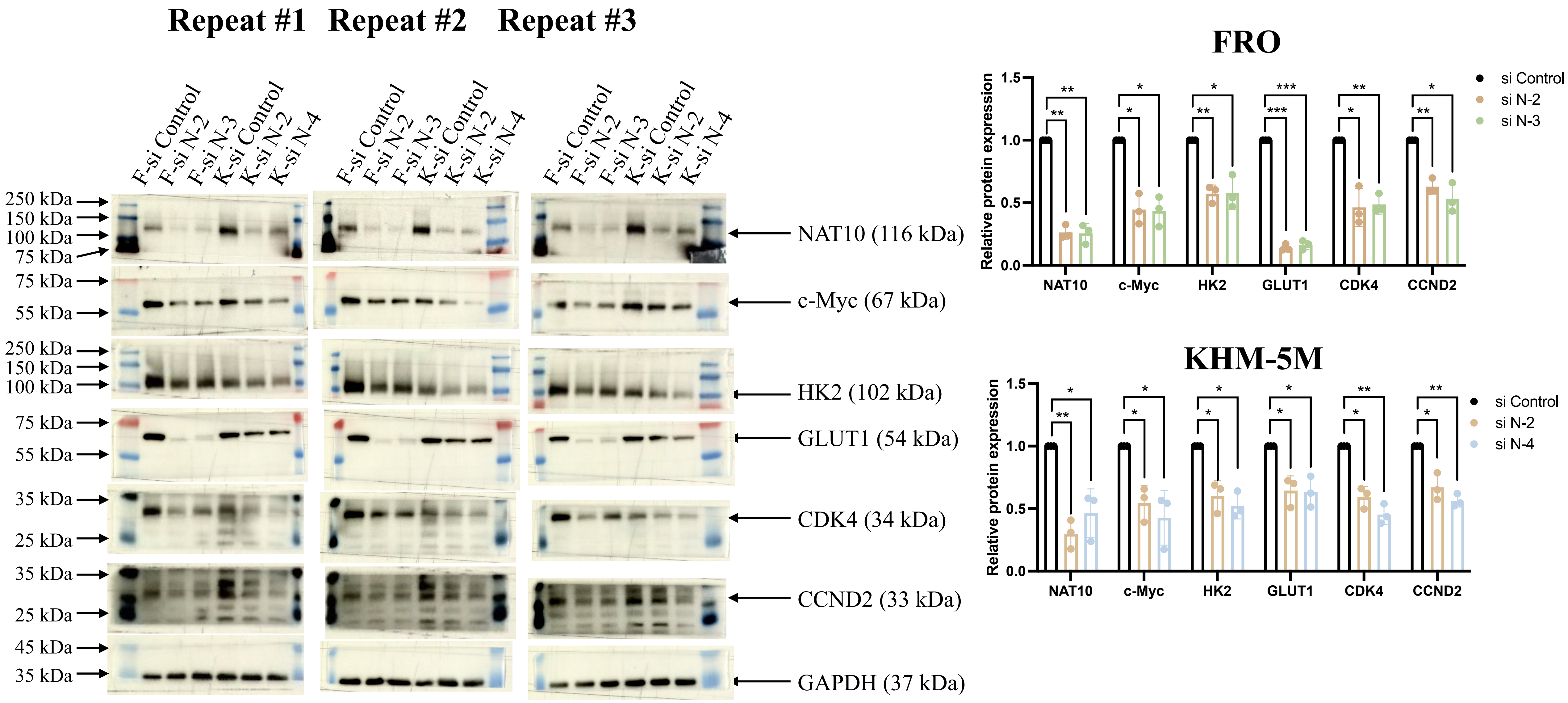
Repeat #1
Repeat #2
Repeat #3
F-si Control
F-si N-2
F-si N-3
K-si Control
K-si N-2
K-si N-4
250 kDa
150 kDa
100 kDa
75 kDa
55 kDa
35 kDa
25 kDa
45 kDa
NAT10 (116 kDa)
c-Myc (67 kDa)
HK2 (102 kDa)
GLUT1 (54 kDa)
CDK4 (34 kDa)
CCND2 (33 kDa)
GAPDH (37 kDa)
FRO
Relative protein expression
si Control
si N-2
si N-3
NAT10
c-Myc
HK2
GLUT1
CDK4
CCND2
KHM-5M
si N-4

**Western Blot Band Densitometry Measurement**

## Repeat #1

| Sample | NAT10 | c-Myc | HK2 | GLUT1 | CDK4 | CCND2 | GAPDH |
|---|---|---|---|---|---|---|---|
| F-si Control | 7095 | 13118 | 25178 | 14915 | 17229 | 23487 | 9424 |
| F-si N-2 | 1887 | 5110 | 17846 | 2225 | 8306 | 16320 | 11077 |
| F-si N-3 | 2862 | 5850 | 19644 | 2692 | 11118 | 17037 | 13634 |
| K-si Control | 14443 | 10452 | 15665 | 12696 | 16896 | 29337 | 12964 |
| K-si N-2 | 4231 | 5813 | 10181 | 8418 | 9949 | 18049 | 12248 |
| K-si N-3 | 6921 | 4663 | 8249 | 7913 | 5335 | 12615 | 10646 |

## Repeat #2

| NAT10 | c-Myc | HK2 | GLUT1 | CDK4 | CCND2 | GAPDH |
|---|---|---|---|---|---|---|
| 11695 | 13295 | 24730 | 13827 | 20678 | 18756 | 13625 |
| 2666 | 7323 | 14720 | 1649 | 12539 | 12533 | 13032 |
| 2000 | 7397 | 18279 | 2370 | 12145 | 12639 | 13893 |
| 13845 | 7834 | 13884 | 11496 | 13604 | 21228 | 9746 |
| 3350 | 4166 | 8686 | 7931 | 9183 | 16514 | 13224 |
| 4022 | 1664 | 8169 | 8743 | 7220 | 14128 | 11883 |

## Repeat #3

| Sample | NAT10 | c-Myc | HK2 | GLUT1 | CDK4 | CCND2 | GAPDH |
|---|---|---|---|---|---|---|---|
| F-si Control | 7913 | 7704 | 19707 | 10044 | 15858 | 21542 | 7751 |
| F-si N-2 | 3122 | 4004 | 11795 | 2089 | 6621 | 15568 | 9375 |
| F-si N-3 | 3898 | 5413 | 14335 | 3029 | 10746 | 14451 | 11981 |
| K-si Control | 16375 | 12911 | 13257 | 10979 | 9240 | 21254 | 13368 |
| K-si N-2 | 6636 | 8427 | 8665 | 7881 | 6054 | 16605 | 13279 |
| K-si N-3 | 9028 | 7088 | 5715 | 5477 | 4846 | 12806 | 12988 |
