# Supplementary material for: The NAT10/c-Myc positive feedback loop orchestrates tRNA ac4C modification and OTUB1-mediated protein stabilization to drive anaplastic thyroid carcinoma progression
Source: Cell Mol Biol Lett. 2026 Jun 12;31:139. doi: 10.1186/s11658-026-00977-x (PMC13523248; doi:10.1186/s11658-026-00977-x)
Supplement: Supplementary file 2 [file 11658_2026_977_MOESM10_ESM.docx]

| **Supplemental Table 2 Antibodies and chemicals** | | | | | |
| --- | --- | --- | --- | --- | --- |
| **REAGENT or RESOURCE** | **SOURCE** | **IDENTIFIER** | **Source** | **Calculated MW** | **Observed MW** |
| **Antibodies** | | | | |  |
| NAT10 | Proteintech | Cat No. 13365-1-AP | Rabbit | 116 kDa | 116 kDa |
| c-MYC | Proteintech | Cat No. 67447-1-Ig | Mouse | 49 kDa | 55-60 kDa |
| N4-acetylcytidine | ABclonal | Cat No.: A18806 | Rabbit |  |  |
| Puromycin | Proteintech | Cat No.: A23031 | Rabbit |  |  |
| RELA | Proteintech | Cat No. 10745-1-AP | Rabbit | 65 kDa | 65 kDa |
| OTUB1 | Proteintech | Cat No. 68489-1-Ig | Mouse | 31 kDa and 35 kDa | 31-35 kDa |
| ubiquitin | Proteintech | Cat No. 10201-2-AP | Rabbit |  |  |
| GAPDH | Proteintech | Cat No. 60004-1-Ig | Mouse | 37 kDa | 37 kDa |
| EMC8 | Proteintech | Cat No. 19889-1-AP | Rabbit | 24 kDa | 24 kDa |
| TPI1 | Proteintech | Cat No. 10713-1-AP | Rabbit | 27 kDa | 27 kDa |
| TTYH3 | Proteintech | Cat No. 24707-1-AP | Rabbit | 58 kDa | 50 kDa |
| Beta Actin | Proteintech | Cat No. 66009-1-Ig | Mouse | 42 kDa | 42 kDa |
| CDK4 | AB clonal | A11136 | Rabbit | 34 kDa | 34 kDa |
| Cyclin D2 | AB clonal | A1773 | Rabbit | 33 kDa | 33 kDa |
| GLUT1/SLC2A1 | AB clonal | A11208 | Rabbit | 54 kDa | 54 kDa |
| HK2 | AB clonal | A22319 | Rabbit | 102 kDa | 102 kDa |
| Alexa Fluor® 596-conjugated secondary antibodies | Cell signalling | Cat No. 8889 |  |  |  |
| HRP-conjugated Goat Anti-Rabbit IgG | Proteintech | Cat No. SA00001-2 |  |  |  |
| HRP conjugated Goat Anti-Mouse IgG | Proteintech | Cat No. SA00001-1 |  |  |  |
| **Chemicals** | | | | | |
| Remodelin | MedChemExpress | Cat No. HY-16706 |  |  |  |
| Doxorubicin hydrochloride | MedChemExpress | Cat No. HY-15142 |  |  |  |
