# Supplementary material for: The NAT10/c-Myc positive feedback loop orchestrates tRNA ac4C modification and OTUB1-mediated protein stabilization to drive anaplastic thyroid carcinoma progression
Source: Cell Mol Biol Lett. 2026 Jun 12;31:139. doi: 10.1186/s11658-026-00977-x (PMC13523248; doi:10.1186/s11658-026-00977-x)
Supplement: Supplementary file 3 [file 11658_2026_977_MOESM9_ESM.docx]

| **Supplemental Table 1 Primers, shRNAs, siRNAs and overexpression** | | |
| --- | --- | --- |
| **Primers** | | |
| **NCBI gene symbol/Name** | **Forward (5'→3')** | **Reverse (3'→5')** |
| **NAT10** | ATAGCAGCCACAAACATTCGC | ACACACATGCCGAAGGTATTG |
| **GAPDH** | GGAGTCCACTGGCGTCTT | GAGTCCTTCCACGATACCAAA |
| **c-Myc** | GTCAAGAGGCGAACACACAAC | TTGGACGGACAGGATGTATGC |
| **RELA** | CGCATCCAGACCAACAACAACC | GCAGAGCCGCACAGCATTC |
| **TPI1** | CCCAGGAAGTACACGAGAAG | CAGTCACAGAGCCTCCATAAA |
| **TTYH3** | TGCTGAATGGCACGGAGGTGAA | CTCCACGCCGTCATAGCAGAAG |
| **EMC8** | AGGTGGCTCTCACCCTGATT | TGGACTGGCATCCTTTACTCG |
| **U6** | CTCGCTTCGGCAGCACA | AACGCTTCACGAATTTGCGT |
| **CDK4** | CATGTAGACCAGGACCTAAGGACA | GATCGTTTCGGCTGGCAA |
| **CCND2** | TTTGCCATGTACCCACCGTC | AGGGCATCACAAGTGAGCG |
| **HK2** | GAGCCACCACTCACCCTACT | CCAGGCATTCGGCAATGTG |
| **GLUT1** | TCTGGCATCAACGCTGTCTTC | CGATACCGGAGCCAATGGT |
| **CHIP-NAT10-1** | AACAACATATATGGACCAAGAGA | AAGACCTGGAGAAATCAGCAA |
| **CHIP-NAT10-2** | GCTTACGTCATTACTCAGCGACA | CGTCCCTCTCAGGAACTGGTA |
| **tRNAs** | | |
|  | **Stem-loop reverse transcription primer** | **Forward primer** |
| **Ser-AGA-1-1** | GTCGTATCCAGTGCAGGGTCCGAGGTATTCGCACTGGATACGACCGTAGT | GTTAAGGCGATGGACTAGAAATCCA |
| **Ser-CGA-4-1** | GTCGTATCCAGTGCAGGGTCCGAGGTATTCGCACTGGATACGACCGTCAC | TGGACTCGAAATCCAATGGGG |
| **Ser-UGA-1-1** | GTCGTATCCAGTGCAGGGTCCGAGGTATTCGCACTGGATACGACCGCAGC | AGTGGTTAAGGCGTTGGACT |
| **Ser-UGA-2-1** | GTCGTATCCAGTGCAGGGTCCGAGGTATTCGCACTGGATACGACCGTAGT | GATGGACTTGAAATCCATTGGGGT |
| **Ser-UGA-4-1** | GTCGTATCCAGTGCAGGGTCCGAGGTATTCGCACTGGATACGACCGTAGC | AGGCGATGGACTTGAAATCC |
| **Leu-CAA-4-1** | GTCGTATCCAGTGCAGGGTCCGAGGTATTCGCACTGGATACGACTGTCAG | CCAGACTCAAGGTAAGCACCTT |
| **Leu-CAA-6-1** | GTCGTATCCAGTGCAGGGTCCGAGGTATTCGCACTGGATACGACTGTCAA | CTGCGTTCAAATCGCACCC |
| **Universal reverse primer** | AGTGCAGGGTCCGAGGTATT | |
| **shRNA** | | |
| **Name** | **Squence (5'-3')** | |
| **NAT10-sh1** | ATAGTCACTGTGTACAATTGC | |
| **NAT10-sh2** | AACAGGAACAAATCCAGCTCG | |
| **NAT10-sh3** | GCGGCAAAGATCTCTCTTTGT | |
| **NAT10-sh4** | GCTGCTGCAGATGTACTATGA | |
| **siRNA** | | |
| **RELA si1 sense** | GGAUUGAGGAGAAACGUAAtt | |
| **RELA si1 anti-sense** | UUACGUUUCUCCUCAAUCCtt | |
| **RELA si2 sense** | GAGUCAGAUCAGCUCCUAAtt | |
| **RELA si2 anti-sense** | UUAGGAGCUGAUCUGACUCtt | |
| **RELA si3 sense** | GCUUCUAUGAGGCUGAGCUtt | |
| **RELA si3 anti-sense** | AGCUCAGCCUCAUAGAAGCtt | |
| **c-MYC si1 sense** | CGACGAGACCUUCAUCAAAtt | |
| **c-MYC si1 anti-sense** | UUUGAUGAAGGUCUCGUCGtt | |
| **c-MYC si2 sense** | CCAAGGUAGUUAUCCUUAAtt | |
| **c-MYC si2 anti-sense** | UUAAGGAUAACUACCUUGGtt | |
| **c-MYC si3 sense** | GGAAGAAAUCGAUGUUGUUtt | |
| **c-MYC si3 anti-sense** | AACAACAUCGAUUUCUUCCtt | |
| **NAT10 si2 sense** | AACAGGAACAAAUCCAGCUCG | |
| **NAT10 si2 anti-sense** | CGAGCUGGAUUUGUUCCUGUU | |
| **NAT10 si3 sense** | GCGGCAAAGAUCUCUCUUUGU | |
| **NAT10 si3 anti-sense** | ACAAAGAGAGAUCUUUGCCGC | |
| **NAT10 si4 sense** | GCUGCUGCAGAUGUACUAUGA | |
| **NAT10 si4 anti-sense** | UCAUAGUACAUCUGCAGCAGC | |
| **Overexpressions** | | |
| **Wild-type NAT10** | atgcatcggaaaaaggtggataaccgaatccggattctcattgagaatggagtagctgagcggcaaagatctctctttgttgtagttggggatcgaggaaaagatcaggtggtaatacttcatcacatgttatccaaagcaactgtgaaggctcggccttcagtgctgtggtgttataagaaagagctggggtttagcagtcaccggaagaaaagaatgcgacagctgcagaagaaaataaagaatggaacactgaacataaagcaggacgacccctttgaactcttcatagcagccacaaacattcgctactgctactacaacgagacccacaagatcctgggcaataccttcggcatgtgtgtgctgcaggattttgaagccttaactccaaacttgctggccaggactgtagaaacagtggaaggtggtgggctagtggtcatcctcctacggaccatgaactcactcaagcaattgtacacagtgactatggatgtgcattccaggtacagaactgaggcccatcaggatgtggtgggaagatttaatgaaaggtttattctgtctctggcctcttgtaagaagtgtctcgtcattgatgaccagctcaacatcctgcccatctcctcccacgttgccaccatggaggccctgcctccccagactccggatgagagtcttggtccttctgatctggagctgagggagttgaaggagagcttgcaggacacccagcctgtgggtgtgttggtggactgctgtaagactctagaccaggccaaagctgtcttgaaatttatcgagggcatctctgaaaagaccctgaggagtactgttgcactcacagctgctcgaggacggggaaaatctgcagccctgggattggcgattgctggggcggtggcatttgggtactccaatatctttgttacctccccaagccctgataacctccatactctgtttgaatttgtatttaaaggatttgatgctctgcaatatcaggaacatctggattatgagattatccagtctctaaatcctgaatttaacaaagcagtgatcagagtgaatgtatttcgagaacacaggcagactattcagtatatacatcctgcagatgctgtgaagctgggccaggctgaactagttgtgattgatgaagctgccgccatccccctccccttggtgaagagcctacttggcccctaccttgttttcatggcatccaccatcaatggctatgagggcactggccggtcactgtccctcaagctaattcagcagctccgtcaacagagcgcccagagccaggtcagcaccactgctgagaataagaccacgacgacagccagattggcatcagcgcggacactgtatgaggtttccctccaggagtcaatccgatacgcccctggggatgcagtggagaagtggctgaatgacttgctgtgcctggattgcctcaacatcactcggatagtctcaggctgccccttgcctgaagcttgtgaactgtactatgttaatagagataccctcttttgctaccacaaggcctctgaagttttcctccaacggcttatggccctctacgtggcttctcactacaagaactctcccaatgatctccagatgctctccgatgcacctgctcaccatctcttctgccttctgcctccTgtgccccccacccagaatgcccttccagaagtgcttgctgttatccaggtgtgccttgaaggggagatttctcgccagtccatcttgaacagtctgtctcgaggcaagaaggcttcaggggacctgattccatggacagtgtcagaacagttccaagatccagactttggtggtctgtctggtggaagggtcgttcgcattgctgttcacccagattatcaagggatgggctatggcagccgtgctctgcagctgctgcagatgtactatgaaggcaggtttccttgtctggaggaaaaggtccttgagacaccacaggaaattcacaccgtaagcagcgaggctgtcagcttgttggaagaggtcatcactccccggaaggacctgcctcctttactcctcaaattgaatgagaggcctgccgaacgcctggattacctgggtgtttcctatggcttgacccccaggctcctcaagttctggaaacgagctggatttgttcctgtttatctgagacagaccccgaatgacctgaccggagagcactcgtgcatcatgctgaagacgctcactgatgaggatgaggctgaccagggaggctggcttgcagccttctggaaagatttccgacggcggttcctagccttgctctcctaccagttcagtaccttctctccttccctggctctgaacatcattcagaacaggaacatggggaagccagcccagcctgccctgagccgggaggagctggaagcactcttcctcccctatgacctgaagcggctggagatgtattcacggaatatggtggactatcacctcatcatggacatgatcccggccatctctcgcatctatttcctgaaccagctgggggacctggccctgtctgcggctcagtcggctcttctcttggggattggcctgcagcataagtctgtggaccagctggaaaaggagattgagctgccctcgggccagttgatgggacttttcaaccggatcatccgcaaagttgtgaagctatttaatgaagttcaggaaaaggccattgaggagcagatggtggcagcgaaggatgtggtcatggagcccacgatgaagaccctcagtgacgacctagatgaagcagcaaaggaatttcaggagaaacacaagaaggaagtagggaagctgaagagcatggacctctctgaatacataatccgtggggacgatgaagagtggaatgaagttttgaacaaagctgggccgaacgcctcgatcatcagcctgaaaagtgacaagaaaaggaagttagaggccaaacaagaacccaaacagagcaagaagttgaagaacagagagacaaagaacaaaaaagatatgaaactgaagcggaagaaa | |
| **NAT10-G641E** | atgcatcggaaaaaggtggataaccgaatccggattctcattgagaatggagtagctgagcggcaaagatctctctttgttgtagttggggatcgaggaaaagatcaggtggtaatacttcatcacatgttatccaaagcaactgtgaaggctcggccttcagtgctgtggtgttataagaaagagctggggtttagcagtcaccggaagaaaagaatgcgacagctgcagaagaaaataaagaatggaacactgaacataaagcaggacgacccctttgaactcttcatagcagccacaaacattcgctactgctactacaacgagacccacaagatcctgggcaataccttcggcatgtgtgtgctgcaggattttgaagccttaactccaaacttgctggccaggactgtagaaacagtggaaggtggtgggctagtggtcatcctcctacggaccatgaactcactcaagcaattgtacacagtgactatggatgtgcattccaggtacagaactgaggcccatcaggatgtggtgggaagatttaatgaaaggtttattctgtctctggcctcttgtaagaagtgtctcgtcattgatgaccagctcaacatcctgcccatctcctcccacgttgccaccatggaggccctgcctccccagactccggatgagagtcttggtccttctgatctggagctgagggagttgaaggagagcttgcaggacacccagcctgtgggtgtgttggtggactgctgtaagactctagaccaggccaaagctgtcttgaaatttatcgagggcatctctgaaaagaccctgaggagtactgttgcactcacagctgctcgaggacggggaaaatctgcagccctgggattggcgattgctggggcggtggcatttgggtactccaatatctttgttacctccccaagccctgataacctccatactctgtttgaatttgtatttaaaggatttgatgctctgcaatatcaggaacatctggattatgagattatccagtctctaaatcctgaatttaacaaagcagtgatcagagtgaatgtatttcgagaacacaggcagactattcagtatatacatcctgcagatgctgtgaagctgggccaggctgaactagttgtgattgatgaagctgccgccatccccctccccttggtgaagagcctacttggcccctaccttgttttcatggcatccaccatcaatggctatgagggcactggccggtcactgtccctcaagctaattcagcagctccgtcaacagagcgcccagagccaggtcagcaccactgctgagaataagaccacgacgacagccagattggcatcagcgcggacactgtatgaggtttccctccaggagtcaatccgatacgcccctggggatgcagtggagaagtggctgaatgacttgctgtgcctggattgcctcaacatcactcggatagtctcaggctgccccttgcctgaagcttgtgaactgtactatgttaatagagataccctcttttgctaccacaaggcctctgaagttttcctccaacggcttatggccctctacgtggcttctcactacaagaactctcccaatgatctccagatgctctccgatgcacctgctcaccatctcttctgccttctgcctccTgtgccccccacccagaatgcccttccagaagtgcttgctgttatccaggtgtgccttgaaggggagatttctcgccagtccatcttgaacagtctgtctcgaggcaagaaggcttcaggggacctgattccatggacagtgtcagaacagttccaagatccagactttggtggtctgtctggtggaagggtcgttcgcattgctgttcacccagattatcaagggatgggctatgAAagccgtgctctgcagctgctgcagatgtactatgaaggcaggtttccttgtctggaggaaaaggtccttgagacaccacaggaaattcacaccgtaagcagcgaggctgtcagcttgttggaagaggtcatcactccccggaaggacctgcctcctttactcctcaaattgaatgagaggcctgccgaacgcctggattacctgggtgtttcctatggcttgacccccaggctcctcaagttctggaaacgagctggatttgttcctgtttatctgagacagaccccgaatgacctgaccggagagcactcgtgcatcatgctgaagacgctcactgatgaggatgaggctgaccagggaggctggcttgcagccttctggaaagatttccgacggcggttcctagccttgctctcctaccagttcagtaccttctctccttccctggctctgaacatcattcagaacaggaacatggggaagccagcccagcctgccctgagccgggaggagctggaagcactcttcctcccctatgacctgaagcggctggagatgtattcacggaatatggtggactatcacctcatcatggacatgatcccggccatctctcgcatctatttcctgaaccagctgggggacctggccctgtctgcggctcagtcggctcttctcttggggattggcctgcagcataagtctgtggaccagctggaaaaggagattgagctgccctcgggccagttgatgggacttttcaaccggatcatccgcaaagttgtgaagctatttaatgaagttcaggaaaaggccattgaggagcagatggtggcagcgaaggatgtggtcatggagcccacgatgaagaccctcagtgacgacctagatgaagcagcaaaggaatttcaggagaaacacaagaaggaagtagggaagctgaagagcatggacctctctgaatacataatccgtggggacgatgaagagtggaatgaagttttgaacaaagctgggccgaacgcctcgatcatcagcctgaaaagtgacaagaaaaggaagttagaggccaaacaagaacccaaacagagcaagaagttgaagaacagagagacaaagaacaaaaaagatatgaaactgaagcggaagaaa | |
| **oe-OTUB1** | atggcggcggaggaacctcagcagcagaagcaggagccgctgggcagcgactccgaaggtgttaactgtctggcctatgatgaagccatcatggctcagcaggaccgaattcagcaagagattgctgtgcagaaccctctggtgtcagagcggctggagctctcggtcctatacaaggagtatgctgaagatgacaacatctatcaacagaagatcaaggacctccacaaaaagtactcgtacatccgcaagaccaggcctgacggcaactgtttctatcgggctttcggattctcccacttggaggcactgctggatgacagcaaggagttgcagcggttcaaggctgtgtctgccaagagcaaggaagacctggtgtcccagggcttcactgaattcacaattgaggatttccacaacacgttcatggacctgattgagcaggtggagaagcagacctctgtcgccgacctgctggcctccttcaatgaccagagcacctccgactaccttgtggtctacctgcggctgctcacctcgggctacctgcagcgcgagagcaagttcttcgagcacttcatcgagggtggacggactgtcaaggagttctgccagcaggaggtggagcccatgtgcaaggagagcgaccacatccacatcattgcgctggcccaggccctcagcgtgtccatccaggtggagtacatggaccgcggcgagggcggcaccaccaatccgcacatcttccctgagggctccgagcccaaggtctaccttctctaccggcctggacactacgatatcctctacaaa | |
